# Immunomodulatory Effects in a Phase II Study of Lenalidomide Combined with Cetuximab in Refractory *KRAS*-Mutant Metastatic Colorectal Cancer Patients

**DOI:** 10.1371/journal.pone.0080437

**Published:** 2013-11-11

**Authors:** Anita K. Gandhi, Tao Shi, Mingyu Li, Ulf Jungnelius, Alfredo Romano, Josep Tabernero, Salvatore Siena, Peter H. Schafer, Rajesh Chopra

**Affiliations:** 1 Department of Translational Development, Celgene Corporation, Summit, New Jersey, United States of America; 2 Department of Scientific Information Systems, Celgene Corporation, San Diego, California, United States of America; 3 Department of Biostatistics and Programming, Celgene Corporation, Summit, New Jersey, United States of America; 4 Clinical Research and Development, Celgene, Summit, New Jersey, United States of America; 5 Vall d'Hebron University Hospital and Vall d'Hebron Institute of Oncology, Universitat Autònoma de Barcelona, Barcelona, Spain; 6 Ospedale Niguarda Ca' Granda, Milan, Italy; Karolinska Institutet, Sweden

## Abstract

This study assessed the immunomodulatory effects in previously treated *KRAS*-mutant metastatic colorectal cancer patients participating in a phase II multicenter, open-label clinical trial receiving lenalidomide alone or lenalidomide plus cetuximab. The main findings show the T cell immunostimulatory properties of lenalidomide as the drug induced a decrease in the percentage CD45RA^+^ naïve T cells 3-fold while increasing the percentage HLA-DR^+^ activated T helper cells and percentage total CD45RO^+^ CD8^+^ memory T cytotoxic cells, 2.6- and 2.1-fold respectively (p<0.0001). In addition, lenalidomide decreased the percentage of circulating CD19^+^ B cells 2.6-fold (p<0.0001). Lenalidomide increased a modest, yet significant, 1.4-fold change in the percentage of circulating natural killer cells. Our findings indicate that lenalidomide significantly activates T cells, suggestive of an immunotherapeutic role for this drug in settings of maintenance therapy and tumor immunity. Furthermore, reported for the first time is the effect of lenalidomide in combination with cetuximab on T cell function, including increases in circulating naïve and central memory T cells. In summary, lenalidomide and cetuximab have significant effects on circulating immune cells in patients with colorectal carcinoma.

**Trial Registration:**

ClinicalTrials.gov NCT01032291

## Introduction

The immunomodulatory drug lenalidomide is an orally active agent with significant activity in a range of hematologic disorders including myelodysplastic syndromes (MDS), multiple myeloma (MM), and non-Hodgkin's lymphoma (NHL).

Lenalidomide has received US Food and Drug Administration approval for the treatment of MM in patients who have received at least one prior therapy, and for the treatment of transfusion-dependent anemia in patients with International Prognostic Scoring System-defined Low- or Intermediate-1-risk MDS with a del(5q) cytogenetic abnormality, with or without additional cytogenetic abnormalities. The molecular target of lenalidomide is the protein cereblon (CRBN). CRBN is a ubiquitously expressed protein and member of the cullin ring ligase 4 (CRL4) E3 ubiquitin ligase complex, and was identified as the primary teratogenic target of thalidomide [1]. CRBN mediates both the antiproliferative activities of lenalidomide in myeloma cells and T cell activation [2], [3]. The immunomodulatory effects of lenalidomide range across several cell types including both lymphoid and myeloid lineages. In particular, *in vitro* data indicate lenalidomide has activity in T cells, T regulatory cells (Tregs), B cells, monocytes, natural killer (NK) T cells, and NK cells.

In anti-CD3 stimulated T cells, lenalidomide stimulates T cell proliferation, and the production of interleukin (IL)-2, IL-12, and interferon gamma [4], [5]. In addition, lenalidomide has been shown to inhibit Tregs proliferation and suppressor function *in vitro* [6]. In advanced solid tumor patients treated with lenalidomide, naïve CD4^+^ helper and CD8^+^ cytotoxic T cells decreased, whereas memory CD4^+^ helper memory T cells and CD8^+^ cytotoxic memory cells increased [7]. Previous studies of the immunomodulatory activity of lenalidomide have been undertaken in hematological malignancies where immunological parameters may be confounded by disease. However, there are limited data describing the immunomodulatory function of lenalidomide in solid tumors.

In a clinical trial aimed to assess the efficacy and safety of combination treatment with lenalidomide and cetuximab in *KRAS* (v-Ki-ras2 Kirsten rat sarcoma viral oncogene homolog)-mutant metastatic colorectal cancer patients, we assessed 25 different subpopulations of CD45^+^ immune cells (T cells, B cells, and NK cells). This was a phase II multicenter, open-label trial comprising a safety lead-in phase (phase IIa) to determine the maximum tolerated dose, and a randomized proof of concept phase (phase IIb) to determine the response rate of lenalidomide plus cetuximab combination therapy. Phase IIa treatment comprised oral lenalidomide (starting dose 25 mg/day) and intravenous cetuximab (400 mg/m^2^ followed by weekly 250 mg/m^2^) in 28-day cycles. In phase IIb patients were randomized to either the phase IIa treatment schedule of lenalidomide plus cetuximab combination therapy or lenalidomide 25 mg/day monotherapy. The combination of lenalidomide and cetuximab appeared to be well tolerated but did not have clinically meaningful activity in *KRAS*-mutant metastatic colorectal cancer patients (12).

## Materials and Methods

### Patients, materials, and methods

This phase II, multicenter, open-label trial was conducted in accordance with the ethical principles of the Declaration of Helsinki and Good Clinical Practice, and according to the International Conference on Harmonisation of Technical Requirements for Registration of Pharmaceuticals for Human Use. The study protocol, the proposed informed consent form, and other information to subjects, were approved by the Comitato Etico-Scientifico, Ospedale Niguarda Ca' Granda, Milan, Italy and properly constituted Institutional Review Boards/Independent Ethics Committees of all participating institutions (Medical Ethics Commission of the UZ KULeuven, Leuven, Belgium; Clinical Research Ethics Committee of the University Hospital of Vall d'Hebron, Barcelona, Spain; Scientific Ethics Committee of the Ospedale Niguarda Ca' Granda, Milan, Italy; Ethics Committee Azienda Ospedaliero-Universitaria San Martino, Genova, Italy; Ethics Committee Azienda Ospedaliero-Universitaria Ospedali Riuniti Umberto I - G.M. Lancisi - G. Salesi di Ancona, Torrette, Italy; Regional Ethics Review Board Stockholm, Karolinska Institute, Sweden; and Flinders Clinical Research Ethics Committee, Southern Australia, Australia). The study was registered at Clinicaltrials.gov with identifier NCT01032291. The protocol for this trial and supporting CONSORT checklist are available as supporting information; see Checklist S1 and Protocol S1.

Written informed consent was obtained from all participants involved in the study. The study consisted of a safety lead-in phase (phase IIa), in which the primary objective was to determine the maximum tolerated dose of lenalidomide in combination with cetuximab in subjects with *KRAS*-mutant mCRC, and a proof of concept (POC) phase (phase IIb), in which the primary objective was to determine the response rate in these subjects per the Response Evaluation Criteria in Solid Tumors (RECIST) version 1.1. A total of 48 of 50 subjects who received drug were evaluated for immune flow cytometric analysis.

### Sample collection

Blood was collected at baseline (cycle 1 day 1 [C1D1]), cycle 2 day 1 (C2D1) and cycle 3 day 1 (C3D1) in either 5 ml Lavender/Black Cyto-Chex® BCT glass tubes (Streck Innovations, Omaha, NE, USA) or 2.6 ml yellow top acid citrate dextrose tubes depending upon where the sample was received and maintained at ambient temperature until flow cytometry was performed at ICON Central Laboratories (Farmingdale, NY, USA) within 72 hours of sample collection. Stability analyses were conducted during assay validation and supports up to 72 hours processing time from sample collection to analysis. Antibodies (anti-CD3, CD4, CD8, CD16, CD19, CD25, CD45, CD45RA, CD45RO, CD62L, CD107a, CD127, granzyme B, and HLA-DR; BD Biosciences, San Jose, CA, USA) and 100 µl of patient whole blood were transferred to 12×75 mm test tubes (BD Falcon™; BD Biosciences) followed by gentle vortexing and 15 min incubation at room temperature. To this, BD Pharm Lyse™ lysing solution (BD Biosciences) was added for 10 min. The tube was centrifuged, washed in PBA (Dulbecco's PBS/0.2%) and the pellet was resuspended in PBA prior to analysis of B, T, and NK cell subsets on a FACSCanto™ II flow cytometer (BD Biosciences). For intracellular protein staining, an additional permeabilization step was added after the first addition of PBA with fixation-permeabilization working solution (eBioscience, Inc., San Diego, CA, USA), followed by two washes in permeabilization buffer (eBioscience, Inc.), incubation with intracellular antibodies, and another permeabilization buffer wash. Appropriate compensation controls were included during each analysis. Each cell subset population was reported as an absolute value per 1 mm^3^ of blood or as a percentage of CD45^+^ cells.

### Statistical analyses

To assess the changes in different immune cell populations at C2D1 and C3D1 versus baseline, each of the 50 flow cytometry measurements (i.e. one absolute cell count and one percentage measurement for each of the 25 subpopulations of CD45+ immune cells) were first log2 transformed and a linear model was then fitted with cycle number (i.e. C1D1, C2D1, and C3D1) as an 3-level independent variable and patient as a blocking variable using the R software package LIMMA [8]. Moderated t-statistics and the corresponding p values for the two contrasts of interested: C2D1 vs. C1D1 and C3D1 vs. C1D1 were computed using the empirical Bayes method implemented in LIMMA. This empirical Bayes step was carried out separately for absolute cell counts and percentage measurements due to their very different scales and variations. For each of the two contrasts, p-values were later combined for absolute cell counts and percentage measurements and were adjusted for multiple comparisons according to the Benjamini-Hochberg method [9]. Adjusted p-values ≤ 0.05 were considered statistically significant, which is equivalent to controlling the false discovery rate at 5%. We carried out above mentioned analyses separately in three different patient populations, i.e. 20 patients from the lenalidomide arm, 28 patients from the lenalidomide plus cetuximab arm, or 48 patients from the two arms combined (main analyses). We also carried out the analyses in the three populations but with the 19 patients who were on a concomitant medication which coded to an anatomic therapeutic chemical category denoting a systemic or topical corticosteroid removed (sensitivity analyses, results not shown). All statistical analyses were carried out using R software [10].

## Results

**Figure 1:**
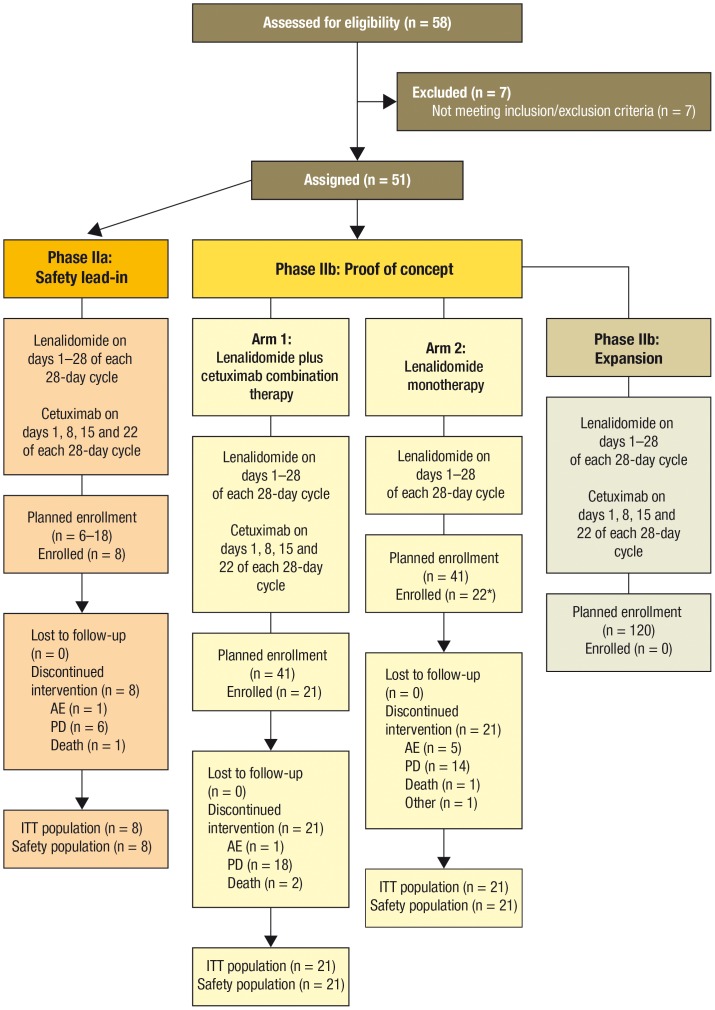
Study design and enrollment in patient groups. Study was terminated before the expansion part of phase IIb. *One patient was randomized to the lenalidomide monotherapy group but discontinued before taking any study drug and was therefore excluded from the analyses. AE, adverse event; ITT, intention to treat; PD, progressive disease.

A total of 48 of 50 subjects who received study drug were evaluable for peripheral blood immune cell analysis. The study schema is shown in Figure 1 and the patient baseline characteristics are summarized in Table 1. There were 20 subjects in the lenalidomide arm and 28 in the lenalidomide plus cetuximab arm. The biomarker and correlative analyses performed and the number of subjects included in each analysis is listed in Table S1. The absolute number and percentage of 25 different subsets of T, B, and NK cells were analyzed in these 48 patients and the cell phenotypes are listed in Table 2. Cell populations with at least one p-value ≤ 0.05 in the comparisons of C2D1 and C3D1 versus C1D1 in the lenalidomide monotherapy arm, the lenalidomide plus cetuximab arm, and across all patients are shown in Tables 3, 4, and 5, respectively.

**Table 1:** Patient baseline characteristics.

		Lenalidomide (n = 21)	Lenalidomide plus cetuximab (n = 29)
Median age, years (range)		54 (38–75)	58 (31–70)
Sex	Male	12 (57.1%)	16 (55.2%)
	Female	9 (42.9%)	13 (44.8%)
ECOG PS score	0	10 (47.6%)	20 (69.0%)
	1	10 (47.6%)	9 (31.0%)
	2	1 (4.8%)	0
Concomitant steroids		7 (33%)	12 (41%)
Previous lines of systemic anticancer therapy	2	5 (23.8%)	9 (31.0%)
	3	4 (19.0%)	6 (20.7%)
	>3	12 (57.1%)	14 (48.2%)

ECOG PS, Eastern Cooperative Oncology Group performance status.

**Table 2:** Cell surface marker phenotype of each immune cell subset assessed by immunoflow cytometry.

Subset	Cell type (phenotype)
WBC	Lymphocytes (CD45)
Lymphocytes	Total T cells (CD45^+^ CD3^+^)
	T helper cells (CD45^+^ CD3^+^ CD4^+^ CD8^−^)
	T cytotoxic cells (CD45^+^ CD3^+^ CD4^−^ CD8^+^)
	NK cells (CD45^+^ CD3^−^ CD16^+^56^+^)
	B cells (CD45^+^ CD3^−^ CD19^+^)
	Total naïve T helper cells (CD45^+^ CD3^+^ CD4^+^ CD8^−^ CD45RA^+^ CD45RO^−^)
	Total memory T helper cells (CD45^+^ CD3^+^ CD4^+^ CD8^−^ CD45RA^−^ CD45RO^+^)
	Total naïve T cytotoxic cells (CD45^+^ CD3^+^ CD4^−^ CD8^+^ CD45RA^+^ CD45RO^−^)
	Total memory T cytotoxic cells (CD45^+^ CD3^+^ CD4^−^ CD8^+^ CD45RA^−^ CD45RO^+^)
T helper	Activated T helper cells (CD45^+^ CD3^+^ CD4^+^ CD8^−^ HLADR^+^)
	Effector memory T helper cells (CD45^+^ CD3^+^ CD4^+^ CD8^−^ CD45RA^−^ 62L^−^)
	Effector T helper cells (CD45^+^ CD3^+^ CD4^+^ CD8^−^ CD45RA^+^ 62L^−^)
	Central memory T helper cells (CD45^+^ CD3^+^ CD4^+^ CD8^−^ CD45RA^−^ 62L^+^)
	Naïve T helper cells (CD45^+^ CD3^+^ CD4^+^ CD8^−^ CD45RA^+^ 62L^+^)
	CD40L^+^ T helper cells (CD3^+^ CD4^+^ CD154^+^)
	T regulatory cells (CD3^+^ CD4^+^ FOX P3^+^ CD127^−^ CD25^++^)
T cytotoxic	Activated T cytotoxic cells (CD45^+^ CD3^+^ CD4^−^ CD8^+^ HLADR^+^)
	Effector memory T cytotoxic cells (CD45^+^ CD3^+^ CD4^−^ CD8^+^ CD45RA^−^ 62L^−^)
	Effector T cytotoxic cells (CD45^+^ CD3^+^ CD4^−^ CD8^+^ CD45RA^+^ 62L^−^)
	Central memory T cytotoxic cells (CD45^+^ CD3^+^ CD4^−^ CD8^+^ CD45RA^−^ 62L^+^)
	Naïve T cytotoxic cells (CD45^+^ CD3^+^ CD4^−^ CD8^+^ CD45RA^+^ 62L^+^)
NK cells	CD107a^+^ NK cells (CD45^+^ CD3^−^ CD16^+^ CD56^+^ CD107a^+^)
	Granzyme B^+^ NK cells (CD45^+^ CD3^−^ CD16^+^ CD56^+^ Granzyme B^+^)
	NKG2D^+^ NK cells (CD45^+^ CD3^−^ CD16^+^ CD56^+^ CD314^+^)

NK, natural killer; WBC, white blood cells.

**Table 3:** Significantly regulated cell populations in the lenalidomide monotherapy arm.

		C2D1 versus C1D1		C3D1 versus C1D1	
		FDR adjusted p-value	FC	FDR adjusted p-value	FC
T cells	Abs total naïve T helper cells	<0.0001	−4.02	0.0743	−2.33
	% Total naïve T helper cells	<0.0001	−3	0.0094	−2.13
	Abs activated T helper cells	0.0135	1.78	0.644	1.28
	% Activated T helper cells	<0.0001	2.65	0.0231	1.81
	Abs activated T cytotoxic cells	0.0226	1.58	0.436	1.42
	% Activated T cytotoxic cells	<0.0001	1.91	0.0094	1.75
	Abs total naïve T cytotoxic cells	0.0008	−2.16	0.227	−1.64
	% Total naïve T cytotoxic cells	0.0001	−1.6	0.0511	−1.49
	Abs effector T helper cells	0.0025	−3.17	0.0094	−6.85
	% Effector T helper cells	0.0181	−2.32	0.0147	−5.5
	Abs effector T cytotoxic cells	0.0099	−1.56	0.129	−1.64
	% Effector T cytotoxic cells	0.0005	−1.31	0.0263	−1.34
	Abs total memory T cytotoxic cells	0.0242	1.51	0.162	1.66
	% Total memory T cytotoxic cells	0.0002	2.08	0.0545	1.87
	Abs effector memory T cytotoxic cells	0.035	1.71	0.812	1.19
	% Effector memory T cytotoxic cells	0.0013	2.08	0.38	1.48
	% T cytotoxic cells	0.017	1.13	0.812	1.04
	Abs T regulatory cells*	0.0047	2.28	0.0094	5.61
	% T regulatory cells*	0.0018	4.4	0.0094	12.1
	Abs T helper cells*	0.0199	−1.49	0.812	−1.1
	Abs total T cells*	0.0318	−1.38	0.812	−1.08
B cells	Abs B cells	0.0001	−3.41	0.191	−1.93
	% B cells	<0.0001	−2.59	0.116	−1.78
NK cells	% NK cells*	0.0318	1.4	1	−1.01
Lymphocytes	Abs lymphocytes	0.0378	−1.36	0.812	−1.1

* Cell populations that are unique to the lenalidomide monotherapy arm only.

Abs, absolute; C1D1, cycle 1 day 1; C2D1, cycle 2 day 1; C3D1, cycle 3 day 1; FC, fold change; NK, natural killer.

**Table 4:** Significantly regulated cell populations in the lenalidomide plus cetuximab arm.

		C2D1 versus C1D1		C3D1 versus C1D1	
		FDR adjusted p-value	FC	FDR adjusted p-value	FC
T cells	Abs total naïve T helper cells	<0.0001	−2.42	0.0002	−3.13
	% Total naïve T helper cells	<0.0001	−2.6	0.0003	−2.71
	Abs activated T helper cells	<0.0001	2.15	0.0526	1.45
	% Activated T helper cells	<0.0001	2.33	<0.0001	2.16
	Abs activated T cytotoxic cells	0.0046	1.83	0.306	1.34
	% Activated T cytotoxic cells	0.0001	1.77	0.0081	1.7
	Abs total naïve T cytotoxic cells	0.0352	−1.34	0.0166	−1.56
	% Total naïve T cytotoxic cells	<0.0001	−1.43	0.0029	−1.34
	Abs effector T helper cells	0.246	−1.7	0.0047	−5.15
	% Effector T helper cells	0.267	−1.59	0.0129	−3.64
	Abs effector T cytotoxic cells	0.4	−1.2	0.0166	−1.87
	% Effector T cytotoxic cells	0.0196	−1.25	0.0043	−1.48
	Abs total memory T cytotoxic cells	<0.0001	2.32	0.0404	1.51
	% Total memory T cytotoxic cells	<0.0001	2.22	0.0029	1.79
	Abs effector memory T cytotoxic cells	0.0046	1.71	0.782	1.08
	% Effector memory T cytotoxic cells	<0.0001	1.65	0.0265	1.37
	% T cytotoxic cells	0.0289	1.15	0.159	1.13
	Abs central memory T cytotoxic cells*	0.321	1.65	0.0047	6.17
	% Central memory T cytotoxic cells*	0.416	2.79	0.0112	62.8
	Abs effector memory T helper cells*	0.678	1.11	0.0166	−2.08
	Abs total memory T helper cells*	0.0211	1.35	0.794	−1.05
	% Total memory T helper cells*	0.0096	1.27	0.346	1.12
	Abs central memory T helper cells*	0.329	1.95	0.0149	8.21
	% Central memory T helper cells*	0.416	2.29	0.0175	20.3
	Abs naïve T cytotoxic cells*	0.678	1.29	0.0166	6.13
	% Naïve T cytotoxic cells*	0.64	1.58	0.0325	11.9
	Abs naïve T helper cells*	0.722	1.28	0.0325	6.86
B cells	Abs B cells	0.0054	−2.01	0.0005	−3.6
	% B cells	0.0018	−2.17	0.0011	−3.16
NK cells	% Granzyme B^+^ NK cells*	0.114	1.15	0.0374	1.25
Lymphocytes	% Lymphocytes	0.469	1.11	0.0337	1.45

* Cell populations that are unique to the lenalidomide plus cetuximab arm only.

Abs, absolute; C1D1, cycle 1 day 1; C2D1, cycle 2 day 1; C3D1, cycle 3 day 1; FC, fold change; NK, natural killer.

**Table 5:** Significantly regulated cell populations in all subjects.

		C2D1 versus C1D1		C3D1 versus C1D1	
		FDR adjusted p-value	FC	FDR adjusted p-value	FC
T cells	Abs total naïve T helper cells	<0.0001	−3.03	<0.0001	−2.94
	% Total naïve T helper cells	<0.0001	−2.77	<0.0001	−2.53
	Abs activated T helper cells	<0.0001	1.98	0.0567	1.38
	% Activated T helper cells	<0.0001	2.47	<0.0001	2.06
	Abs activated T cytotoxic cells	0.0001	1.72	0.132	1.35
	% Activated T cytotoxic cells	<0.0001	1.83	<0.0001	1.72
	Abs T regulatory cells	0.0105	1.88	0.0027	3.03
	% T regulatory cells	0.0098	3.41	0.0162	5.21
	Abs total naïve T cytotoxic cells	<0.0001	−1.66	0.0038	−1.63
	% Total naïve T cytotoxic cells	<0.0001	−1.5	<0.0001	−1.39
	Abs effector T helper cells	0.0021	−2.23	<0.0001	−5.84
	% Effector T helper cells	0.0097	−1.87	0.0001	−4.24
	Abs effector T cytotoxic cells	0.0173	−1.34	0.0015	−1.82
	% Effector T cytotoxic cells	<0.0001	−1.27	<0.0001	−1.44
	Abs total memory T cytotoxic cells	<0.0001	1.92	0.0148	1.52
	% Total memory T cytotoxic cells	<0.0001	2.16	<0.0001	1.81
	Abs effector memory T cytotoxic cells	0.0001	1.71	0.622	1.11
	% Effector memory T cytotoxic cells	<0.0001	1.83	0.0148	1.42
	% T cytotoxic cells	0.0005	1.15	0.0979	1.1
	Abs central memory T cytotoxic cells	0.144	1.55	0.0003	4.46
	% Central memory T cytotoxic cells	0.253	2.5	0.0037	25.8
	Abs effector memory T helper cells	0.724	−1.07	0.0017	−2.1
	% Effector memory T helper cells	0.182	1.15	0.0163	−1.43
	% Total memory T helper cells	0.0005	1.24	0.272	1.1
	Abs central memory T helper cells	0.179	1.8	0.0038	5.75
	% Central memory T helper cells	0.144	2.6	0.0037	14
	Abs naïve T cytotoxic cells	0.724	1.16	0.0052	4.32
	% Naïve T cytotoxic cells	0.531	1.49	0.0163	7.63
	Abs naïve T helper cells	0.956	−1.02	0.038	3.87
	% Naïve T helper cells	0.843	−1.14	0.0429	6.21
B cells	Abs B cells	<0.0001	−2.54	<0.0001	−3.05
	% B cells	<0.0001	−2.35	<0.0001	−2.66
NK cells	% Granzyme B^+^ NK cells	0.179	1.09	0.0284	1.21
Lymphocytes	% Lymphocytes	0.918	−1.01	0.0291	1.33

Abs, absolute; C1D1, cycle 1 day 1; C2D1, cycle 2 day 1; C3D1, cycle 3 day 1; FC, fold change; NK, natural killer.

**Figure 2:**
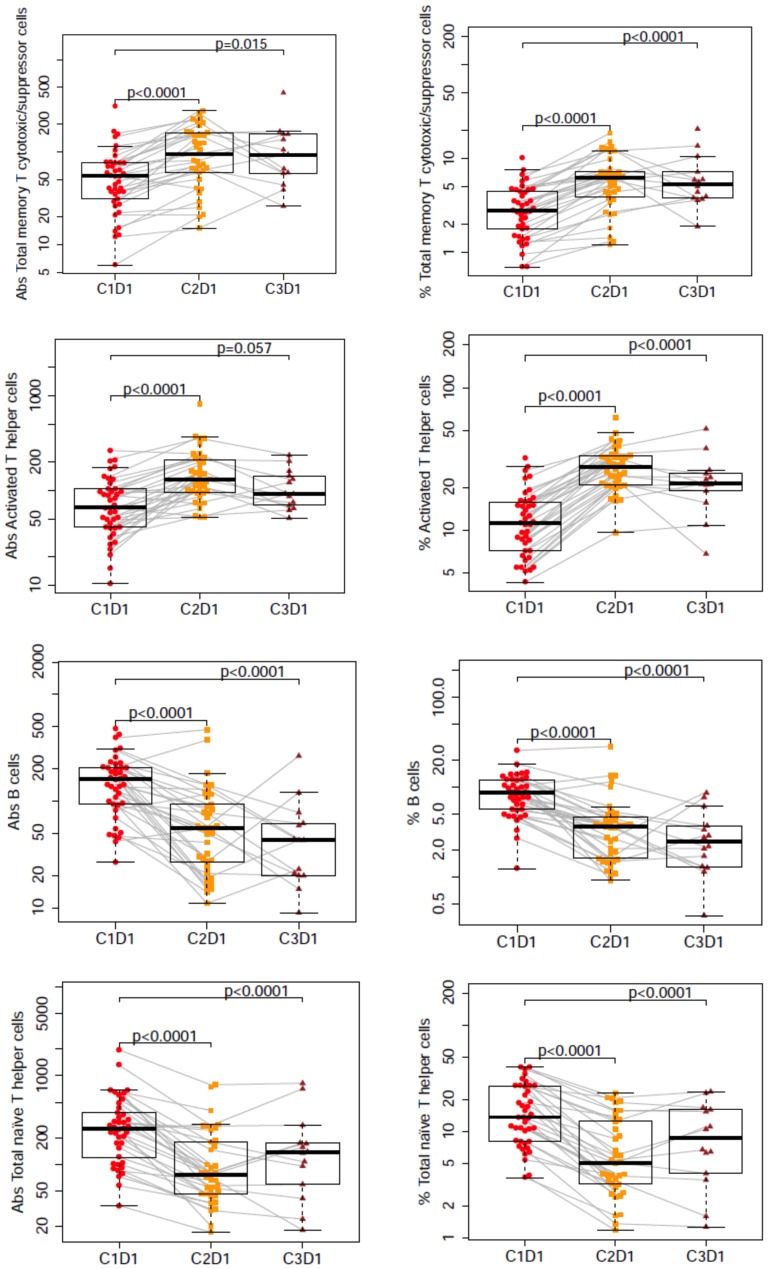
Significant changes in number in four immune cell subsets. Significant (p ≤ 0.0001) changes in percentage and absolute number in four immune cell subsets from cycle 1 day 1 (C1D1) to cycle 2 day 1 (C2D1) or cycle 3 day 1 (C3D1) in all subjects. The upper edge of the box denotes the 75th percentile whereas the lower edge denotes the 25th percentile. The line inside each box is the median. The lines extend to the maximum and minimum values excluding outliers. The gray lines denote individual subject data. Abs, absolute.

The major findings include four immune cell populations with highly significant (p ≤ 0.0001) changes from C1D1 (baseline) to C2D1 and to C3D1 in both the absolute number and the percentage of cells measured in both treatment arms. The changes in these populations are shown in Figure 2 and include an increase in total memory T cytotoxic cells and activated T helper cells, and a decrease in B cells and total naïve T helper cells. The changes in the remaining cell subsets across all subjects are shown in Figure S1.

### Immunomodulatory effects in subjects receiving lenalidomide only

In the lenalidomide only arm (n = 20), 12 T cell populations, total NK cells, total B cells, and total lymphocyte cell populations (either percentage or absolute count) were significantly changed (p≤0.05) in either C2D1 or C3D1 versus C1D1, or both. These T cell populations, starting with the most significant, include total naïve T helper cells, activated T helper cells, activated T cytotoxic cells, T regulatory cells, total naïve T cytotoxic cells, effector T helper cells, effector T cytotoxic cells, total memory T cytotoxic cells, effector memory T cytotoxic cells, T helper cells, T cytotoxic cells, and total T cells. Absolute and percentage B cells decreased 1.78- to 3.41-fold. The percentage of NK cells significantly increased at C2D1 by 1.4-fold across subjects taking lenalidomide only. The absolute lymphocytes significantly decreased 1.36-fold across subjects taking lenalidomide only (Table 3). Of note, contrary to *in vitro* data showing lenalidomide inhibits Tregs expansion [11], lenalidomide significantly increased the percentage of Tregs by 4- to 12-fold.

### Immunomodulatory effects in subjects receiving lenalidomide plus cetuximab

In the lenalidomide plus cetuximab arm (n = 28), 15 T cell populations, 1 NK cell population, total B cells, and total lymphocyte cell populations (either percentage or absolute count) were significantly changed (p ≤ 0.05) in either C2D1 or C3D1 versus C1D1, or both. These T cell populations, starting with the most significant, include activated T helper cells, total memory T cytotoxic cells, total naïve T helper cells, total naïve T cytotoxic cells, effector memory T cytotoxic cells, activated T cytotoxic cells, effector T cytotoxic cells, central memory T cytotoxic cells, effector T helper cells, effector memory T helper cells, total memory T helper cells, central memory T helper cells, naïve T cytotoxic cells, T cytotoxic cells, and naïve T helper cells. Absolute and percentage B cells decreased 2.01- to 3.6-fold. The percentage of granzyme B^+^ NK cells significantly increased at C2D1 by 1.15-fold and at C3D1 by 1.25-fold in subjects taking lenalidomide plus cetuximab. The percentage of lymphocytes significantly increased 1.11- to 1.45-fold in subjects taking lenalidomide plus cetuximab (Table 4). Of these, the following seven subpopulations were significantly modulated in the lenalidomide plus cetuximab arm, but not in the lenalidomide arm only: central memory T cytotoxic cells, effector memory T helper cells, total memory T helper cells, central memory T helper cells, naïve T cytotoxic cells, naïve T helper cells, and granzyme B^+^ NK cells. Of note, addition of cetuximab to lenalidomide did not result in an increase in Tregs as was observed by lenalidomide alone.

### Immunomodulatory effects in all subjects

Across all 48 subjects, 16 T cell populations, 1 NK cell populations, total B cells, and total lymphocytes (either percentage or absolute count) were significantly modulated (p ≤ 0.05) in either C2D1 or C3D1 versus C1D1, or both. These T cell populations, starting with the most significant, include activated T helper cells, total naïve T helper cells, total memory T cytotoxic cells, total naïve T cytotoxic cells, activated T cytotoxic cells, effector memory T cytotoxic cells, effector T helper cells, effector T cytotoxic cells, central memory T cytotoxic cells, effector memory T helper cells, T regulatory cells, central memory T helper cells, naïve T cytotoxic cells, total memory T helper cells, T cytotoxic cells, and naïve T helper cells. Absolute and percentage B cells decreased between 2.35- and 3.05-fold. The percentage of granzyme B^+^ NK cells significantly increased 1.21-fold at C3D1, whereas the percentage of lymphocytes increased 1.33-fold at C3D1 across all subjects (Table 5).

### Effects of concomitant immunosuppressive agents

Overall, 19 of the 48 patients were identified as being on either daily or intermittent, concomitant systemic or topical corticosteroids. Of these 19 patients on concomitant corticosteroids for various periods of time, 5 received dexamethasone (ranging from 8 mg daily to 10 mg weekly), 10 received prednisone (ranging from 25 mg twice-daily to 4 mg daily), 5 received betamethasone (ranging from 4 mg daily to 4 mg as needed), and 5 received ultrapotent topical corticosteroids. To rule out any possible effect of these immunosuppressive corticosteroid therapies on the expression of any of the cell populations, a sensitivity analysis was performed excluding the patients on concomitant immunosuppressive medications and the analysis was reperformed. In the sensitivity analyses, the same four cell populations consistently showed significant (p ≤ 0.0001) changes from C1D1 (baseline) to C2D1 or C3D1 in both measurements assessed (absolute number and percentage) signifying these effects are not due to steroid usage. In fact, patients on immunosuppressive concomitant medications had similar magnitude of immune effects (T cell activation and B cell inhibition), and a greater number of immune cell subsets modulated compared with patients not on concomitant therapies. In addition, an analysis of immune changes in concomitant steroid users versus nonusers was performed and the results were comparable (data not shown).

### Correlations of immunomodulatory effects with clinical response

As reported by Siena et al. [12], 8 patients were enrolled into the phase IIa maximum tolerated dose portion of the study and 43 patients were enrolled into the phase IIb POC portion. Best response was stable disease (SD) in 9 patients and study enrollment was terminated prematurely due to lack of efficacy in any of the treatment arms and failure to achieve the planned response objective. There were no significant (p ≤ 0.05) correlations between changes from baseline (C1D1) to C2D1 or C3D1 of any immune cell population in overall survival (OS) regardless of treatment arm (data not shown).

## Discussion

Lenalidomide is approved for use in both MM and MDS, and has reported clinical activity in chronic lymphocytic leukemia (CLL) and NHL [13], [14]. Much of the activity of lenalidomide in these hematological malignancies has been attributed to cell autonomous killing effects. In a clinical trial reported by Siena et al (12) aimed to assess the efficacy and safety of combination treatment with lenalidomide and cetuximab in *KRAS* (v-Ki-ras2 Kirsten rat sarcoma viral oncogene homolog)-mutant metastatic colorectal cancer patients, we assessed 25 different subpopulations of CD45^+^ immune cells (T cells, B cells, and NK cells) in this patient population that does not have confounding hematological abnormalities. The major findings are significant (p ≤ 0.0001) changes from baseline to C2D1 and to C3D1 in the following four lymphocyte populations: B cells (decreased), activated T helper cells (increased), total memory T cytotoxic cells (increased), and total naïve T helper cells (decreased). One potential clinical relevance of these findings implicate lenalidomide is an activator of T cells which may play a role in its anti-tumor activity. In addition, the downregulation of B cell counts may be linked to its activity in B cell malignancies. These effects were detected in both treatment arms. As all subjects were treated with lenalidomide, these changes may be ascribed to the pharmacodynamic effects of lenalidomide. The correlative analyses showed that changes in the cell populations (absolute number or percentage) tested did not correlate with treatment arm, response per RECIST version 1.1 criteria, or OS. The clinical responses from this study are reported in detail by Siena et al. [12].

Furthermore, lenalidomide plus cetuximab had remarkable effects (up to 60-fold increases) in central memory and naïve T cells suggestive that cetuximab itself may play a role in T cell activation and function. Similar effects were reported by Botta et al. [15] in a cetuximab-based polychemotherapy regimen in patients with advanced mCRC. This is the first study, to our knowledge, to report a comprehensive profile of the circulating immune cell subsets in subjects receiving lenalidomide and to show evidence of the T cell activating function of lenalidomide plus cetuximab.

Patients with mCRC administered with the B cell depleting agent rituximab have shown both regression of metastases and achievement of SD [16]. In our study, although the majority of patients treated with lenalidomide showed significant decreases in B cells, this pharmacodynamic effect did not correlate with OS. Similarly, a related analog pomalidomide, has been reported to reduce CD19^+^ peripheral blood B cells in patients with MM receiving alternate day dosing of 1–10 mg [17].

Preclinical and clinical data indicate that lenalidomide activates T cells. Bartlett et al. [7] showed that in patients with advanced solid tumors, treatment with lenalidomide for 4–5 weeks decreased the percentages of naïve helper and cytotoxic T cells, and increased the percentages of memory helper and cytotoxic cells—both of these findings were confirmed in the present study. Pomalidomide has also been reported to increase peripheral blood CD3^+^ and CD8^+^ T cells in patients with MM [17].

*In vitro*, lenalidomide inhibits Tregs; however, in this study an upward trend (p<0.05) in both absolute number and percentage of circulating Tregs in peripheral blood was observed at both C2D1 and C3D1 in subjects taking lenalidomide alone. Similarly, the level of circulating Tregs was increased in patients with MM within 2 weeks of receiving lenalidomide [18]. These patients with MM showed a significant increase in HLA-DR^+^ T cells (both CD4^+^ and CD8^+^ subsets). The increase in CD4^+^FOX-P3^+^ regulatory T cells was observed after 9 cycles (9 months) of lenalidomide maintenance therapy. Therefore, the increase in regulatory T cells may be a late response to increased effector T cell numbers and activity.

The activation of NK cells by lenalidomide has been shown *in vitro* by both increased NK cell cytokine production as well as in functional studies. In a peripheral blood mononuclear cell/tumor cell co-culture model, T cell co-stimulation leads to enhanced NK cell-mediated lysis of various tumor cells, including prostate and ovarian cancer cells [19]. Lenalidomide enhances NK cell and monocyte-mediated antibody dependent cellular cytotoxicity (ADCC) of rituximab against a variety of hematological cell lines *in vitro*, including NHL and B-CLL [20], as well as enhancing NK cell mediated lysis of cetuximab and trastuzumab coated colorectal and breast cancer cells, respectively [21]. In the present study, although no significant effects were observed in changes of peripheral NK cells, a non-significant increasing trend was observed in both absolute number and percentage of total NK cells and activated NK cells (granzyme B^+^ and NKD^+^) after 28-days of dosing versus baseline. These data do not preclude the possibility that lenalidomide increased NK cells and ADCC at the site of the tumor. Our data previously indicated that dexamethasone antagonizes the stimulatory capacity of lenalidomide on both NK and T cells *in vitro* [22]. The effect of dexamethasone on lenalidomide activation of NK cells in patients with MM indicates that although the number of circulating NK cells increases after lenalidomide plus dexamethasone treatment, the cytotoxic capacity of NK cells becomes impaired due to dexamethasone-induced suppression of IL-2 production from CD4^+^ T cells [23]. To control for possible pharmacodynamic effects of concomitant steroid use on changes from baseline on the populations of CD45^+^ cells analyzed in the present study, sensitivity analyses were performed to exclude data for subjects who had used a systemic corticosteroid or a potent topical corticosteroid during the study. The results of the sensitivity analyses were consistent with those of the main analysis, indicating that the inhibition of B cells is a true pharmacodynamic effect of lenalidomide and this effect is not confounded by intermittent concomitantly administered immunosuppressive therapies. The doses and frequency of dexamethasone taken in this study were insufficient to antagonize the T cell effects of lenalidomide, consistent with previous findings that low-dose dexamethasone allows for greater T cell activation by lenalidomide.

In conclusion, despite preclinical evidence, the present clinical data from Siena et al (12) suggest the modulating effect of lenalidomide is unable to overcome primary resistance of *KRAS*-mutant mCRC to EGFR targeted inhibition by cetuximab. Importantly, these data illustrate the immunomodulatory effects of lenalidomide which helps in understanding the drug's mechanism and potential applicability to other tumor types.

## Supporting Information

Figure S1 Changes in percentage or absolute number in the remaining 21 immune cell subsets from cycle 1 day 1 (C1D1) to cycle 2 day 1 (C2D1) or cycle 3 day 1 (C3D1) in all subjects. The upper edge of the box denotes the 75^th^ percentile whereas the lower edge denotes the 25^th^ percentile. The line inside each box is the median. The lines extend to the maximum and minimum values excluding outliers. The gray lines denote individual subject data. Abbreviation: Abs: absolute.
(DOC)

Table S1 Biomarker and correlative analyses performed and number of subjects included in each analysis. ^a^1 patient was enrolled but did not receive study drug. Abbreviation: Len: lenalidomide.
(DOC)

Checklist S1 CONSORT Checklist.
(DOC)

Protocol S1 Trial Protocol.
(PDF)
