# Supplementary figures and images for: Immunomodulatory Effects in a Phase II Study of Lenalidomide Combined with Cetuximab in Refractory KRAS-Mutant Metastatic Colorectal Cancer Patients
Source: PLoS One. 2013 Nov 11;8(11):e80437. doi: 10.1371/journal.pone.0080437 (PMC3823649; doi:10.1371/journal.pone.0080437)

**Figure S1.**


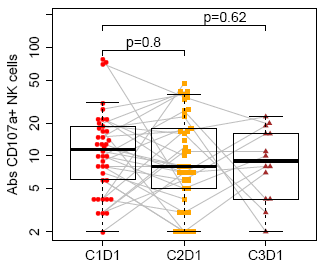

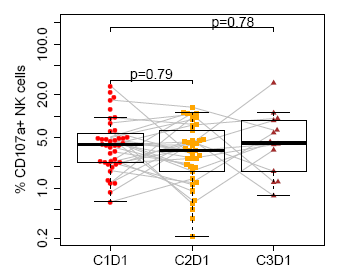


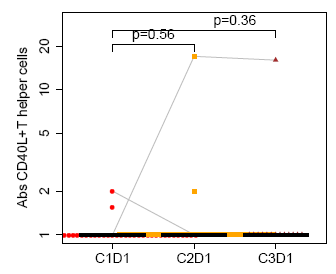

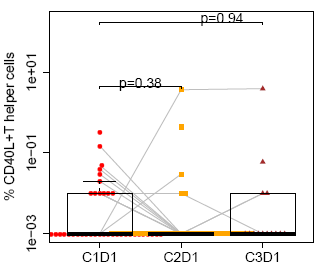


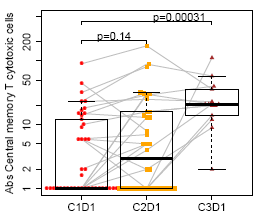

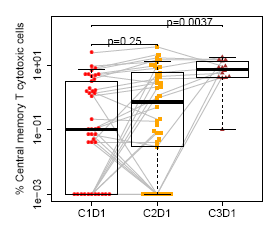


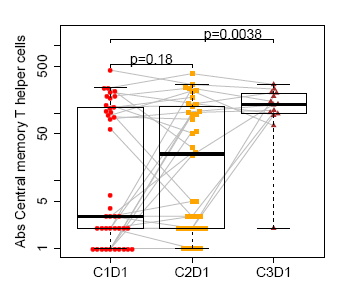

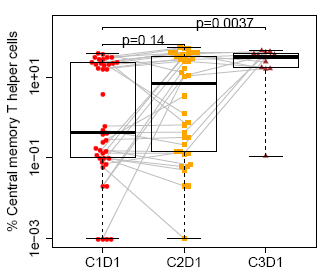


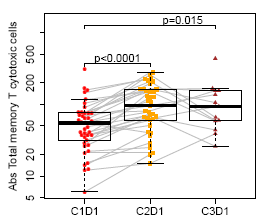

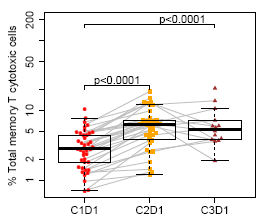


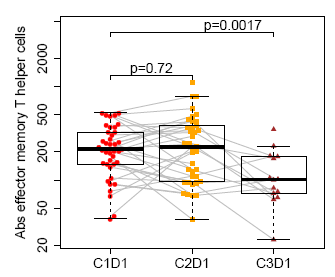

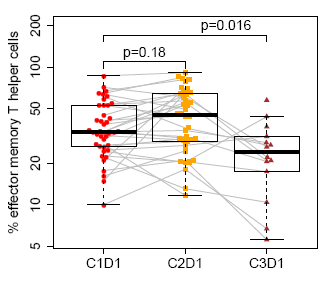


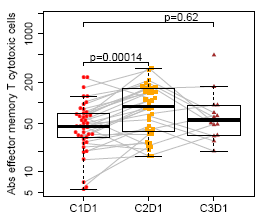

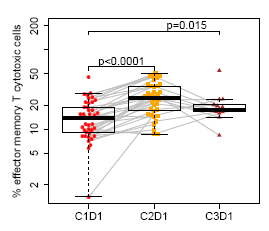


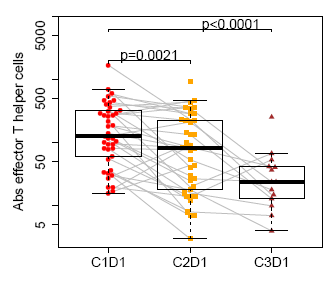

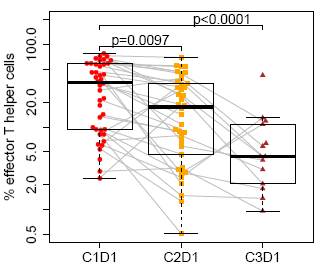


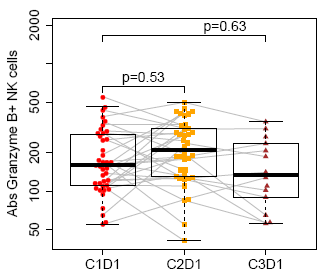

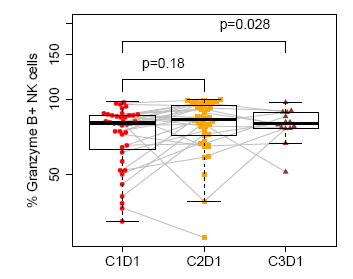


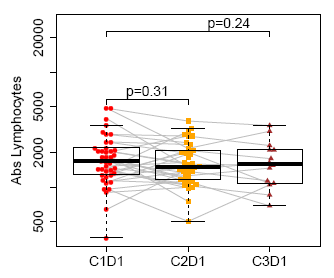

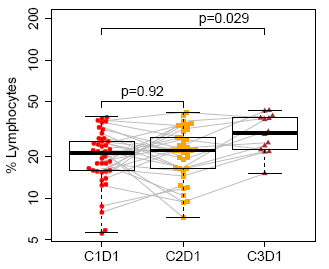


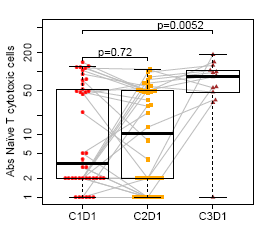

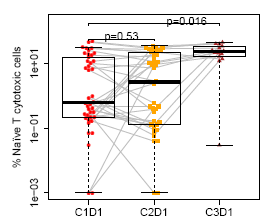


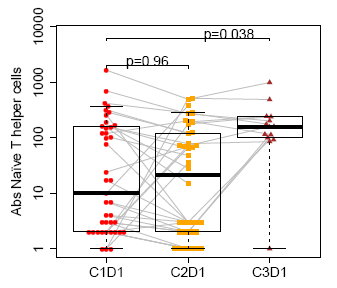

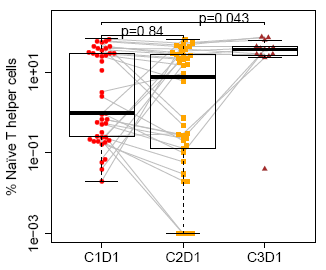


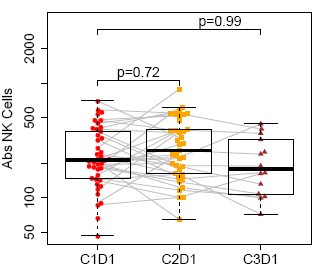

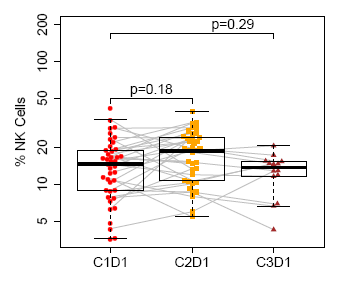


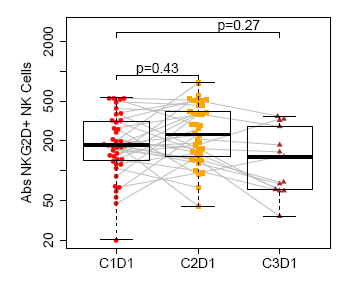

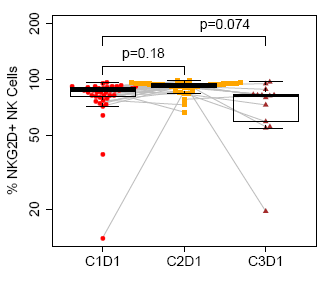


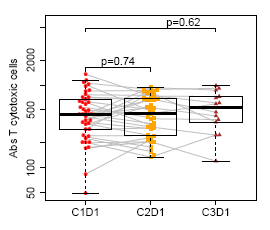

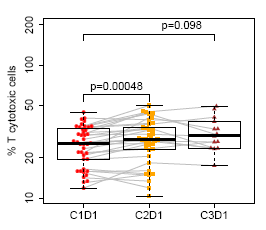


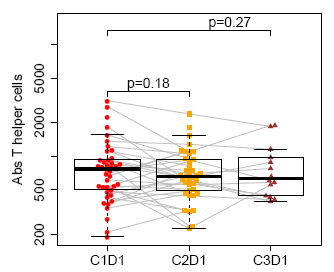

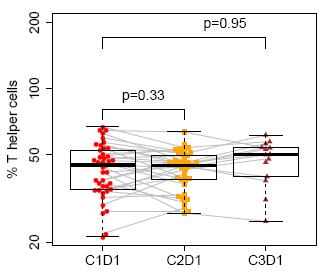


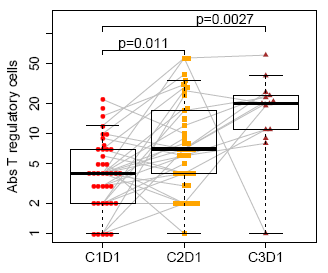

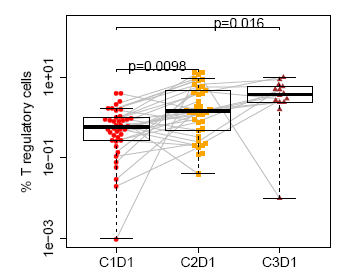


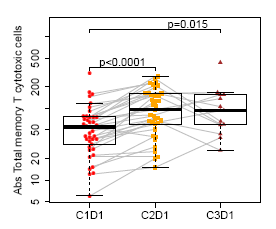

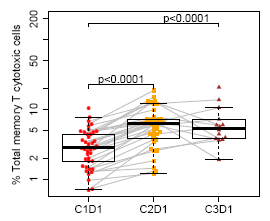


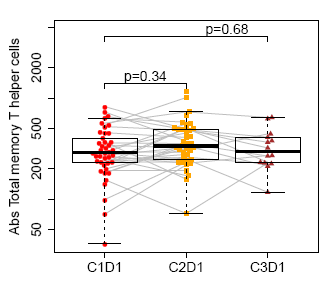

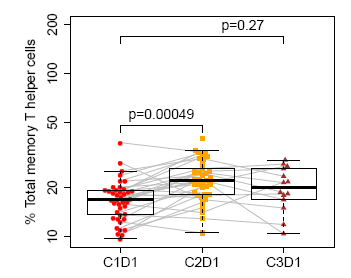


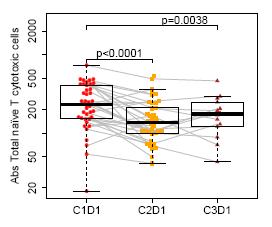

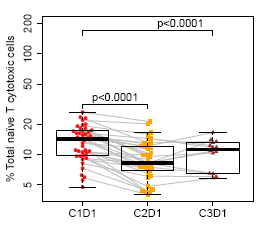


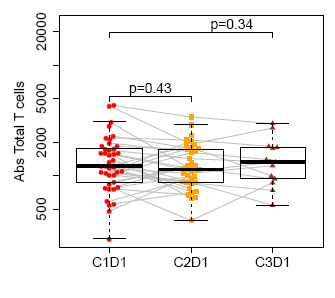

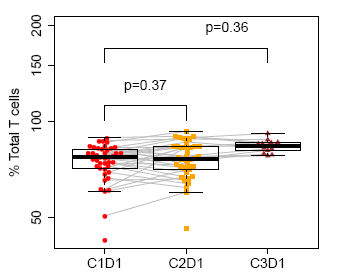

Supplement: Figure S1 — Changes in percentage or absolute number in the remaining 21 immune cell subsets from cycle 1 day 1 (C1D1) to cycle 2 day 1 (C2D1) or cycle 3 day 1 (C3D1) in all subjects. The upper edge of the box denotes the 75th percentile whereas the lower edge denotes the 25th percentile. The line inside each box is the median. The lines extend to the maximum and minimum values excluding outliers. The gray lines denote individual subject data. Abbreviation: Abs: absolute. (DOC) [file pone.0080437.s001.doc]
