# Supplementary material for: Immunomodulatory Effects in a Phase II Study of Lenalidomide Combined with Cetuximab in Refractory KRAS-Mutant Metastatic Colorectal Cancer Patients
Source: PLoS One. 2013 Nov 11;8(11):e80437. doi: 10.1371/journal.pone.0080437 (PMC3823649; doi:10.1371/journal.pone.0080437)
Supplement: Table S1 — Biomarker and correlative analyses performed and number of subjects included in each analysis. a1 patient was enrolled but did not receive study drug. Abbreviation: Len: lenalidomide. (DOC) [file pone.0080437.s002.doc]

**Table S1.**

|  | **Phase IIa** | **Phase IIb** | | | **Total (N = 50)** |
| --- | --- | --- | --- | --- | --- |
|  | **Len + cetuximab (n = 8)^a^** | **Len (n = 21)** | **Len + cetuximab (n = 21)** | |  |
| Both arms (main analysis) | 7 (87.5%) | 20 (95.2%) | 21 (100%) | 48 (96%) | |
| Both arms (sensitivity analysis) | 7 (87.5%) | 13 (61.9%) | 9 (42.9%) | 29 (58%) | |
| Len arm (main analysis) | 0 | 20 (95.2%) | 0 | 20 (40%) | |
| Len arm (sensitivity analysis) | 0 | 13 (61.9%) | 0 | 13 (26%) | |
| Len + cetuximab arm (main analysis) | 7 (87.5%) | 0 | 21 (100%) | 28 (56%) | |
| Len + cetuximab arm (sensitivity analysis) | 7 (87.5%) | 0 | 9 (42.9%) | 16 (32%) | |
