# Supplementary material for: Immunomodulatory Effects in a Phase II Study of Lenalidomide Combined with Cetuximab in Refractory KRAS-Mutant Metastatic Colorectal Cancer Patients
Source: PLoS One. 2013 Nov 11;8(11):e80437. doi: 10.1371/journal.pone.0080437 (PMC3823649; doi:10.1371/journal.pone.0080437)
Supplement: Protocol S1 — Trial Protocol. (PDF) [file pone.0080437.s004.pdf]

A PHASE 2, OPEN-LABEL STUDY TO ASSESS THE EFFICACY AND SAFETY OF LENALIDOMIDE IN COMBINATION WITH CETUXIMAB IN PRETREATED SUBJECTS WITH KRAS MUTANT METASTATIC COLORECTAL CANCER

**STUDY DRUG:**	Lenalidomide (CC-5013)
**PROTOCOL NUMBER:**	CC-5013-COLO-001
**DATE FINAL:**	29 June 2009
**EudraCT NUMBER:**	2009-012665-61

____

Signature of Celgene Corporation Therapeutic Area Head

Jarl Ulf Jungnelius

____

Printed Name of Celgene Corporation Therapeutic Area Head

Date Signed ___________________
dd mmm yyyy

CONFIDENTIAL

This protocol is provided to you as an Investigator, potential Investigator, or consultant for review by you, your staff, and ethics committee/institutional review board. The information contained in this document is regarded as confidential and, except to the extent necessary to obtain informed consent, may not be disclosed to another party unless such disclosure is required by law or regulations. Persons to whom the information is disclosed must be informed that the information is confidential and may not be further disclosed by them.

COORDINATING PRINCIPAL INVESTIGATOR SIGNATURE PAGE

[include if applicable]

Signature of Coordinating Principal Investigator **dd mmm yyyy**

Printed Name of Coordinating Principal Investigator and Title

Site Number________

By my signature, I agree to supervise and oversee the conduct of this study and to ensure its conduct is in compliance with the protocol, informed consent, IRB/EC procedures, instructions from Celgene Corporation representatives, the Declaration of Helsinki, ICH Good Clinical Practice guidelines, and the applicable parts of the United States Code of Federal Regulations and local regulations governing the conduct of clinical studies.

SITE PRINCIPAL INVESTIGATOR SIGNATURE PAGE

Signature of Site Principal Investigator **dd mmm yyyy**

Printed Name of Site Principal Investigator and Title

Site Number________

By my signature, I agree to personally supervise the conduct of this study at my study site and to ensure its conduct is in compliance with the protocol, informed consent, IRB/EC procedures, instructions from Celgene Corporation representatives, the Declaration of Helsinki, ICH Good Clinical Practice guidelines, and the applicable parts of the United States Code of Federal Regulations and local regulations governing the conduct of clinical studies.

1. STUDY CONTACT INFORMATION

Table 1: Celgene Corporation Emergency Contact Information

Role in Study	Name	Address and Telephone Number
Study Manager	Mark Awadalla or designee	86 Morris Avenue<br>Summit, NJ 07901<br>Phone: 908-673-9244<br>Fax: 908-673-2775<br>mawadalla@celgene.com
Responsible Clinical Research Physician	Mark Jones, M.D. or designee	86 Morris Avenue<br>Summit, NJ 07901<br>Phone: 908-673-9245<br>Fax: 908-673-2774<br>mmjones@celgene.com
Drug Safety Contact	Global Drug Safety<br>(North America)	86 Morris Avenue<br>Summit, NJ 07901<br>Phone: 908-673-9667<br>Fax: 908-673-9115<br>drugsafety@celgene.com
	International<br>(ex-North America)	Celgene International Sarl<br>Att: Drug Safety Department<br>Route de Perreux 1<br>2017 Boudry – CH<br>Phone line: +41 (0)32 729 8776<br>Fax line: +41 (0)32 729 8709<br>drugsafetyeurope@celgene.com
24-Hour emergency contact	Mark Jones, M.D. or designee	86 Morris Avenue<br>Summit, NJ 07901<br>Phone: 908-307-3582<br>Fax: 908-673-2774<br>mmjones@celgene.com

2. SYNOPSIS

Name of Sponsor/Company: Celgene Corporation

Name of Investigational Product: Lenalidomide (CC-5013)

Protocol Number: CC-5013-COLO-001

Protocol Title: A Phase 2, open-label study to assess the efficacy and safety of lenalidomide in combination with cetuximab in pretreated subjects with KRAS mutant metastatic colorectal cancer

Indication: Metastatic colorectal cancer (CRC)

**Study Duration**	**Phase of development**
Only subjects with documented KRAS mutant tumors will be included. Screening period for up to 28 days.<br>This Phase 2 study will be conducted in three parts; Phase 2a - Safety Lead-In, Phase 2b - Proof of Concept (POC), and Phase 2b - Expansion. All subjects enrolled will continue to receive study treatment until documented tumor progression, unacceptable toxicity, death, or treatment discontinuation for any other reason.<br>Study Follow-up period will consist of a follow-up visit 28 days after last dose of investigational product(s). Subjects will subsequently be contacted by telephone by the study sites for survival every 90 days until death or 5 years post-discontinuation.	Phase 2

Objectives

Primary

- Phase 2a
  - To determine the Maximal Tolerated Dose (MTD) of lenalidomide in combination with cetuximab in subjects with KRAS mutant metastatic CRC.
- Phase 2b
  - To determine the response rate in subjects with KRAS mutant metastatic CRC.

Secondary (all phases)

- To evaluate the safety and tolerability in subjects with KRAS mutant metastatic CRC.
- To assess the clinical efficacy in subjects with KRAS mutant metastatic CRC.

Exploratory (all phases)

- Analysis of biomarkers as measures for validation of clinical efficacy and toxicity.

Study Endpoints

Primary:

- Phase 2a
  - MTD
- Phase 2b
  - Response Rate according to Response Evaluation Criteria in Solid Tumors 1.1 (RECIST 1.1) (Eisenhauer, 2009).

Secondary (all phases)

- Progression Free Survival (PFS).
- Safety / Tolerability (type, frequency, severity, and relationship of adverse events to investigational products).
- Duration of Response.
- Disease Control Rate (DCR).
- Overall Survival (OS).

Exploratory (all phases)

- Assessment of biomarkers including, but not limited to:
  - Immuno flow cytometry panel for T cells and NK cells.
  - Cytokines/immune markers.
  - Growth factors.
  - Circulating tumor cells.
  - FCgR polymorphisms.
  - EGFR copy number.

Study Design:

This is a Phase 2, multicenter, open-label study of lenalidomide in combination with cetuximab in subjects with KRAS mutant metastatic CRC. The primary objective of the study is to determine the MTD in Phase 2a and response rate in Phase 2b. The secondary objectives are to evaluate the safety, tolerability, and efficacy.

Subjects will be screened within 28 days of study entry as outlined in Table 2. Laboratory testing will be performed by a central laboratory for this study.

Phase 2

Phase 2 study will be conducted in three parts:

1. Phase 2a – Safety Lead-In: up to approximately 18 subjects treated with lenalidomide in combination with cetuximab to establish the MTD of the combination.

2. Phase 2b – (POC): two-arms, up to approximately 82 subjects treated with lenalidomide in combination with cetuximab (41 subjects) or lenalidomide as a single agent (41 subjects).
3. Phase 2b – Expansion: approximately 120 subjects treated with lenalidomide in combination with cetuximab.

Phase 2a – Safety Lead-In

Initially 6 subjects will be enrolled and treated with a daily oral dose of lenalidomide at 25 mg administered on Days 1-28 of each 28-day cycle and intravenous (IV) infusions of cetuximab (400 mg/m^2 initial infusion only, then 250 mg/m^2 subsequently) administered on Days 1, 8, 15, and 22 of each 28-day cycle.

- If < 2 of the 6 subjects experience a Dose Limiting Toxicity (DLT), then the Phase 2b – POC part will start with lenalidomide at 25 mg.
- If ≥ 2 of the 6 subjects experience a DLT, then 6 additional subjects will be enrolled at lenalidomide 20 mg.
  - If < 2 of the additional 6 subjects experience a DLT, then the Phase 2b – POC part will start with lenalidomide at 20 mg.
  - If ≥ 2 of the additional 6 subjects experience a DLT, then 6 additional subjects will be enrolled at lenalidomide 15 mg.
    - If < 2 of the additional 6 subjects experience a DLT, then the Phase 2b – POC part will start with lenalidomide at 15 mg.
    - If ≥ 2 of the additional 6 subjects experience a DLT, then Celgene Corporation will discuss with the investigators and reassess the doses.

Cycles will be repeated every 28 days. All subjects will continue on investigational product (s) until documented tumor progression, unacceptable toxicity, death, or treatment discontinuation for any other reason.

Definition of Dose Limiting Toxicity for the Phase 2a – Safety Lead-In Part

DLT is defined as one or more of the following:

- Total of ≥ 7 days of lenalidomide and/or ≥ 1 dose of cetuximab missed during the first cycle due to drug-related toxicity as outlined below:
  - Any Grade 3 or 4 non-hematological toxicity (excluding alopecia), even after optimally treated with supportive measures.
    - A rash with suspected relationship to cetuximab, treated and resolved in accordance with the guidelines will not be considered a DLT.
  - Grade 4 neutropenia or febrile neutropenia.
  - Grade 4 thrombocytopenia.

Should any subject discontinue the study prior to completing the entire first cycle for reasons other than a DLT or if ≥ 7 days of lenalidomide and/or ≥ 1 dose of cetuximab were missed during the first cycle for reasons other than a DLT, then a replacement

subject will be added at that dose level.

The occurrence of one of the above drug-related toxicities will result in a clinical and/or laboratory assessment to be performed within 7 days following the initial finding to examine the subject for resolution of the toxicity. Lack of resolution of any of these toxicities according to the guidelines above will be considered a DLT. Subjects who experience a DLT may continue on treatment at the Investigator's discretion at a lower dose of lenalidomide in accordance with Table 6.

Adverse events will be graded using the National Cancer Institute Common Terminology Criteria for Adverse Events (NCI CTCAE) Version 4.0 (http://ctep.cancer.gov/protocolDevelopment/electronic_applications/docs/ctcaev4.pdf).

Following Cycle 1, all subjects will continue on treatment until documented tumor progression, unacceptable toxicity, death, or treatment discontinuation for any other reason.

Study visits will occur as outlined in Table 2, which provides the details of dosing by cycle day.

Phase 2b – Proof of Concept

Once dosing has been established in the Safety Lead-in part, POC will be established with up to approximately 82 additional subjects enrolled to receive oral lenalidomide in combination with cetuximab (at the MTD determined during the Safety Lead-in part of the study) or as a single agent. Subjects meeting all eligibility criteria will be enrolled into one of the following two treatment arms randomized 1:1 ratio:

- Arm 1 (up to 41 subjects): Daily oral lenalidomide (dose determined from Safety Lead-in part) on Days 1-28 plus IV infusions of cetuximab (400 mg/m^2 initial infusion, then 250 mg/m^2 subsequently) administered on Days 1, 8, 15, and 22 of each 28-day cycle.
- Arm 2 (up to 41 subjects): Daily oral lenalidomide 25 mg on Days 1-28 of each 28-day cycle.

Study treatment will continue until documented tumor progression, unacceptable toxicity, death, or treatment discontinuation for any other reason.

The study may proceed to Phase 2b – Expansion part if the response rate (per RECIST 1.1 criteria) from either arm in the Phase 2b – POC part is deemed significantly higher than 10%.

Phase 2b - Expansion

Subjects meeting all eligibility criteria will take oral lenalidomide (dose determined from Safety Lead-in part) on Days 1-28 plus IV infusions of cetuximab (400 mg/m^2 initial infusion, then 250 mg/m^2 subsequently) administered on Days 1, 8, 15, and 22 of each 28-day cycle. Celgene Corporation will reassess the feasibility of adding a single agent lenalidomide arm to the Phase 2b – Expansion part of the study depending on results from the Phase 2b – POC part.

Study treatment will continue until documented tumor progression, unacceptable toxicity,

death, or treatment discontinuation for any other reason.

Dose modifications will be determined as per Section 10.3 of the protocol.

Follow-Up Period

All study subjects who discontinue from the study for any reason other than withdrawal of consent, will enter the Follow-up period that includes one follow-up visit 28 days after last dose and telephone contacts that will occur to determine survival every 90 days until death or 5 years post-discontinuation.

Number of planned subjects

Up to approximately 220 total subjects (up to approximately 18 subjects in the Phase 2a – Safety Lead-in part, up to approximately 82 subjects in the Phase 2b – POC part, and approximately 120 subjects in Phase 2b – Expansion part) will be enrolled at multiple sites globally.

Study Population

Key Inclusion Criteria

Subjects must meet all of the following inclusion criteria to be eligible for enrollment into the study:

Disease Characteristics:

1. Histologically or cytologically confirmed metastatic colorectal adenocarcinoma.
2. At least one unidimensionally measurable lesion.
3. Documented KRAS mutant tumor (paraffin tumor blocks or approximately 25 unstained slides must be sent for KRAS mutant testing and status reported by central pathology lab prior to study enrollment).
4. Imaging confirmed (CT/MRI) disease progression within 42 days of previous treatment.
5. Disease progression on oxaliplatin- AND irinotecan-containing regimens in the metastatic setting, with at least one of these regimens containing bevacizumab.

ECOG:

6. Eastern Cooperative Oncology Group (ECOG) performance status of ≤ 1.

Pregnancy:

7. Women of childbearing potential (WCBP) must undergo pregnancy testing based on the frequency outlined in Section 21.3 or 21.4 and pregnancy results must be negative.
8. Unless practicing complete abstinence from heterosexual intercourse, sexually active WCBP must agree to use adequate contraceptive methods as specified in Section 21.3 or 21.4.

9. Males (including those who have had a vasectomy) must use barrier contraception (condom) when engaging in sexual activity with WCBP as specified in Section 21.3 or 21.4.
10. Males must agree not to donate semen or sperm during the duration specified in Section 21.3 or 21.4.
11. All subjects must:
    - Understand that the investigational product could have a potential teratogenic risk.
    - Agree to abstain from donating blood while taking investigational product and following discontinuation of investigational product (see Section 21.3 or 21.4).
    - Agree not to share study medication with another person.
    - Be counseled about pregnancy precautions and risks of fetal exposure (see Section 21.3 or 21.4).

General:

12. Understand and voluntarily sign an informed consent form (ICF).
13. Age ≥ 18 years at the time of signing the ICF.
14. Able to adhere to the study visit schedule and other protocol requirements.

Key Exclusion Criteria

Subjects must not have met any of the following exclusion criteria to be eligible for enrollment into the study.

Prior Treatment:

1. Use of chemotherapy, hormonal therapy, immunotherapy, or any other anticancer or experimental therapy ≤ 28 days prior to Cycle 1 Day 1.
2. Radiotherapy to ≥ 30% of the bone marrow.
3. Surgery ≤ 28 days prior to Cycle 1 Day 1 (minimally invasive procedures for the purpose of diagnosis or staging of the disease are permitted).
4. Prior therapy with cetuximab.
5. Prior therapy with panitumumab.
6. Prior therapy with pomalidomide (CC-4047), lenalidomide, or thalidomide.

Laboratory:

7. Absolute neutrophil count (ANC) < 1.5 x 10^9/L.
8. Platelet count < 100 x 10^9/L.
9. Creatinine Clearance < 50 mL/min (by Cockroft- Gault).
10. Bilirubin > 1.5 x Upper Limit Normal (ULN) (> 2.0 x ULN in the presence of

Gilbert's Syndrome).

11. Serum aspartate transaminase (AST) or alanine aminotransferase (ALT) > 3.0 x ULN (> 5 x ULN in the presence of liver metastases).
12. Hemoglobin < 9 g/dL.

Other Disease State:

13. Untreated, symptomatic brain metastases (brain imaging not required).
14. Venous thromboembolism ≤ 6 months prior to Cycle 1 Day 1.
15. Current congestive heart failure (New York Heart Association class II-IV).
16. Myocardial Infarction (MI) ≤ 12 months prior to Cycle 1 Day 1.
17. Uncontrolled hypertension.
18. Prior malignancies within 5 years, with the exception of treated basal cell/squamous cell carcinoma of the skin or "in-situ" carcinoma of the cervix or breast.

General:

19. Any condition (psychological, familial, sociological or geographical), including the presence of laboratory abnormalities, which would place the subject at unacceptable risk if he/she were to participate in the study, confound the ability to interpret data from the study, or not allow the subject to comply with the requirements of the study protocol.
20. Pregnant or lactating women.

Investigational product, dosage and mode of administration

Celgene Corporation will supply lenalidomide (CC-5013) as 5, 10, 15, 20, and 25 mg capsules to be administered orally once daily for Days 1-28 of each 28-day cycle. Lenalidomide will be packaged in open-label bottles containing enough capsules for 28 days of dosing. All subjects will be required to take a single capsule each day for Days 1-28 of each cycle.

Celgene Corporation will also supply commercial cetuximab labeled as investigational medicinal product (IMP) for administration via IV infusion on Days 1, 8, 15, and 22 of each 28-day cycle. Cetuximab will be administered at 400 mg/m^2 initial dose, then 250 mg/m^2 weekly.

Premedication for cetuximab administration is permitted as needed.

Assessments

Efficacy

- Tumor Assessment.
- Tumor Response Rate according to RECIST 1.1.
- ECOG Performance Status.

- Overall Survival.

Safety:

- Adverse event reporting.
- Concomitant medications.
- Physical examination.
- Vital signs.
- Hematology laboratory evaluations.
- Chemistry laboratory evaluations.
- Pregnancy testing.
- Thyroid function test.

Exploratory:

- Biomarker analysis.

Statistical Analysis:

Statistical Overview:

This Phase 2 study in pretreated KRAS mutant subjects with metastatic colorectal cancer consists of three parts. Phase 2a – Safety Lead-In part is to explore the dosing of lenalidomide in combination with cetuximab. Phase 2b – POC part is to explore tumor response rate of lenalidomide in combination with cetuximab and lenalidomide alone. Phase 2b – Expansion part will enroll approximately 120 subjects to further evaluate the response rate of lenalidomide in combination with cetuximab in pretreated KRAS mutant subjects with metastatic colorectal cancer.

Sample size:

During Phase 2a – Safety Lead-in part, up to approximately 18 subjects will be enrolled.

In the Phase 2b – POC part, up to approximately 82 subjects will be randomized in a 1:1 ratio between lenalidomide in combination with cetuximab and lenalidomide alone. A Simon two stage minimax design will be used to monitor subject enrollment for each randomization arm separately. In the first stage, 23 subjects will be enrolled. If ≤ 2 of the 23 subjects (< 10%) have a response in either arm, the enrollment for that arm will be stopped. If > 2 of the 23 subjects have a response in either arm, the enrollment in that arm will continue until 41 subjects are enrolled. If one arm is stopped, all new subjects will be enrolled in the remaining arm. At the final analysis, the regimen will be concluded with more than 10% true response rate if ≥ 9 of 41 subjects (>21%) have a response. This design will have 90% power to conclude the true response rate is higher than 10% at one-sided 2.5% level when the true response rate is 30%.

When any arm from the Phase 2b – POC is considered positive, the study may proceed with that regimen to the Phase 2b –Expansion phase. In the Expansion phase,

approximately 120 subjects will be treated with the regimen. This sample size will allow for a two sided 95% confidence interval of (22%, 39%) when 30% response rate is observed.

Demographics, Disposition and Dosing

All analyses will be performed by study Phase and treatment group. The baseline characteristics of treated subjects will be summarized. An accounting will be made of all subjects who received investigational product(s) and, in particular, the number of subjects who died or withdrew during treatment will be specified together with the reasons for withdrawal.

Safety Analysis:

Data from all subjects who receive one or more doses of drug will be incorporated into the safety analyses. Investigational product(s) exposure will be summarized. Adverse events, vital sign measurements, clinical laboratory information, and concomitant medications will be tabulated and summarized by group and regimen. All toxicities will be summarized by relative and absolute frequency, severity grade based on the NCI CTCAE version 4.0 and relationship to treatment. Serious adverse events (SAE) will be listed separately. Safety information obtained during the Follow-up period during each segment will be incorporated into these analyses. Graphical displays will be provided where useful in the interpretation of results.

Efficacy Analysis:

All treated subjects will be included for efficacy analysis. Efficacy will be analyzed once all subjects have withdrawn from the study or completed at least 6 cycles. The hypothesis test for the objective confirmed response rate will be based on the exact binomial distribution for the Phase 2b – Expansion part. Ninety-five percent confidence intervals will be provided. A case-by-case description of all subjects who exhibited a complete or partial response during the study will be provided. A descriptive analysis of other evidence of anti-tumor activity will be provided based on clinical, radiographic, and biologic assessments of efficacy. If there are sufficient data, Kaplan-Meier estimates will be provided for duration of response, PFS, DCR, and OS.

Data listings will be provided for all relevant data collected during the studies.

Table 2: Table of Assessments

Assessments	Screening Period	Treatment Period (28 Day Treatment Cycle)		Follow-Up Period	
	-28 to -1	Every Cycle Day 1 (Pre-dose)[1]	Discontinuation from Treatment	28 Days After Last Dose (+/- 3 days)	Every 90 Days After Last Dose (+/- 14 days)
**Entry Assessments**[1]					
Informed Consent	X				
Inclusion/Exclusion Criteria	X				
Complete Medical History	X				
Prior Therapies[2]	X				
**Safety Assessments**					
Adverse Event Query	After signing ICF and until 28 days after last dose				
Physical Examination[3]	X	X	X	X	
Vital Signs[4]	X	X	X	X	
Height	X				
Weight/BSA[5]	X	X	X	X	
Hematology Labs[6]	X	X	X	X	
Serum Chemistry Labs[7]	X	X	X	X	
Thyroid Function Tests[8]	X		X		
Pregnancy Test[9]	X	X	X	X	
Concomitant Medications/ Procedures	After signing ICF and until 28 days after last dose				
**Efficacy Assessments**					
ECOG Performance Status	X	X	X		
Tumor Assessments[10]	X	X[11]	X[12]		
Response Assessments		X[13]	X[13]		
Survival[14]					X
**Exploratory Assessments**					
Blood for Biomarker		X[15]	X[15]		
Tumor for Biomarker	X[16]				
**Investigational Product**					
Dispense Investigational Product(s)[17]		X			
Drug Accountability[17]		X	X		

[1] Screening assessments completed within 14 days prior to Cycle 1 Day 1 need not be repeated at Cycle 1 Day 1. Assessments may be done up to 3 days prior to Day 1 for all other cycles. During Cycle 1, visits will occur every 7 days to assess physical examination, vital signs, haematology laboratory evaluations, and serum chemistry laboratory evaluations. Paraffin tumor blocks or approximately 25 unstained slides must be sent for KRAS mutant testing and status reported by central pathology lab prior to study enrollment.

[2] All prior radiotherapy, relevant surgeries, and chemotherapies and approximate dates of therapies must be recorded.

Safety Assessments

[3] A complete physical exam is required. The Investigator should describe/capture any abnormal findings. Occurs every 7 days during Cycle 1.

[4] Vital signs include pulse, blood pressure, and temperature. Occurs every 7 days during Cycle 1.

[5] The dose of chemotherapy does not need to be changed unless the calculated dose, according to BSA changes by ≥ 10%.

[6] Hematology: RBC count, hemoglobin, hematocrit, WBC count and differential (including ANC) and platelet count. Occurs every 7 days during Cycle 1. May be repeated more frequently if clinically indicated.

[7] Serum Chemistry: Sodium, potassium, chloride, CO_2, calcium, magnesium, phosphorus, BUN, creatinine, glucose, albumin, total protein, alkaline phosphatase, bilirubin, AST/SGOT, ALT/SGPT, LDH and uric acid. Occurs every 7 days during Cycle 1. Any or all may be repeated more frequently if clinically indicated.

[8] Thyroid Stimulating Hormone (TSH) and free T3 and T4 levels will be determined. May be repeated more frequently if clinically indicated.

[9] WCBP who participate in the study will have urine β-HCG levels assessed as a means of pregnancy testing. The subject may not receive investigational product(s) until the investigator has verified that the results of these pregnancy tests are negative (see specifics in Section 21.3 or 21.4).

Efficacy Assessments

[10] Conventional (or spiral) CT/MRI) scans are required. Subjects having received a scan during the Screening Period are not required to have a scan at Cycle 1 Day 1.

[11] Conventional (or spiral) CT/MRI scans are required every 2 cycles beginning with Cycle 3 Day 1. Scans must be done within 7 days prior to Day 1 of the cycle. To ensure comparability, baseline methods and on-study methods for tumor assessment must be performed using identical techniques. Chest X-ray may be performed only when clinically indicated.

[12] Scan should be performed at treatment discontinuation unless last scan was performed within the prior 28 days.

[13] Tumor response will be evaluated every 2 cycles beginning with Cycle 3 Day 1 and at treatment discontinuation. Response and progression will be evaluated using the RECIST 1.1

criteria. All subjects with evidence of objective tumor response (CR and PR) should have the response confirmed at the next scheduled scan (every 2 cycles following Cycle 1 Day 1).

[14] Survival data will be captured via phone contact every 90 days until death or 5 years post-discontinuation.

Exploratory Assessments

[15] Blood for biomarker assessment will be collected at Cycle 1 Day 1, Cycle 2 Day 1, at each tumor assessment, and at discontinuation.

[16] Tumor (paraffin blocks or slides) for biomarker assessment must be collected at Screening. Paraffin tumor blocks or approximately 25 unstained slides must be sent for KRAS mutation testing and results reported by central pathology laboratory prior to study enrollment.

Investigational Product

[17] Celgene Corporation will supply lenalidomide as 5, 10, 15, 20, and 25 mg capsules to be administered PO once daily for Days 1-28 of each 28-day treatment cycle. Celgene Corporation will supply commercial cetuximab labeled as IMP for administration via IV infusion on Days 1, 8, 15, and 22 of each 28-day cycle. Cetuximab will be administered at 400 mg/m^2 initial dose, then 250 mg/m^2 weekly. Premedication for cetuximab administration is permitted as needed. Lenalidomide and cetuximab combination treatment will start on Day 1.

Figure 1 Study Design Schema

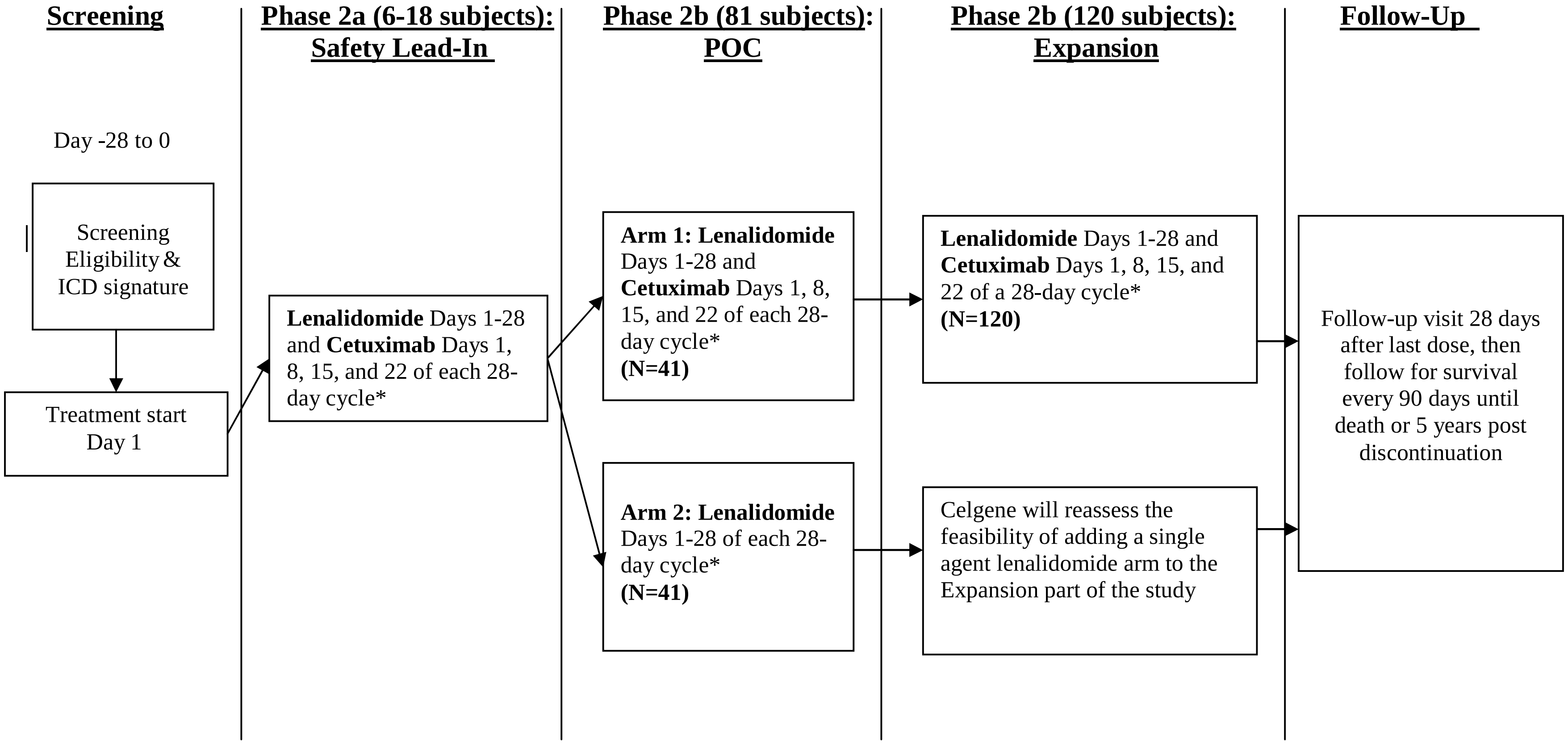

* Continuous treatment until documented tumor progression, unacceptable toxicity, death, or treatment discontinuation for any other reason

3. TABLE OF CONTENTS

TITLE PAGE 1
1. STUDY CONTACT INFORMATION 4
2. SYNOPSIS 5
3. TABLE OF CONTENTS 18
4. LIST OF ABBREVIATIONS AND DEFINITIONS OF TERMS 25
5. INTRODUCTION 28
5.1. Colorectal Cancer 28
5.2. Background and Rationale 28
5.2.1. Treatment of Colorectal Cancer 28
5.2.2. Treatment with Anti-EGFR Therapies and KRAS Gene Mutation Status 29
5.2.2.1. Impact of KRAS Mutational Status on Anti-EGFR Therapies 29
5.2.3. Lenalidomide 30
5.2.4. Lenalidomide Nonclinical Findings 31
5.2.4.1. Immunomodulatory Activity of Lenalidomide 31
5.2.4.2. Anti-angiogenic and Anti-metastatic Activity of Lenalidomide 32
5.2.5. Lenalidomide Dosing and Schedule Rationale 33
5.2.6. Study Rationale 33
6. STUDY OBJECTIVES 35
6.1. Primary 35
6.1.1. Phase 2a 35
6.1.2. Phase 2b 35
6.2. Secondary (all phases) 35
6.3. Exploratory (all phases) 35
7. STUDY ENDPOINTS 36
7.1. Primary 36
7.1.1. Phase 2a 36
7.1.2. Phase 2b 36
7.2. Secondary 36
7.3. Exploratory 36
8. OVERALL STUDY DESIGN 37

8.1. Phase 2a – Safety Lead-In 37
8.1.1. Safety Lead-In Dose Finding....37
8.1.2. Safety Lead-In Definition of Dose Limiting Toxicity....38
8.1.3. Safety Lead-In Definition of MTD....38
8.2. Phase 2b – Proof of Concept 39
8.3. Phase 2b – Expansion....39
8.4. Follow-Up Period 40
8.5. Duration of Treatment 40
9. STUDY POPULATION....41
9.1. Subject Inclusion Criteria 41
9.1.1. Disease Characteristics....41
9.1.2. ECOG 41
9.1.3. Pregnancy 41
9.1.4. General....42
9.2. Subject Exclusion Criteria 42
9.2.1. Prior Treatment....42
9.2.2. Laboratory....42
9.2.3. Other Disease State....42
9.2.4. General....43
10. DESCRIPTION OF TREATMENT....44
10.1. Study Treatment....44
10.1.1. Lenalidomide 44
10.1.2. Cetuximab....44
10.2. Treatment Assignments 44
10.3. Dose Modification or Interruption....44
10.3.1. Lenalidomide Dose Modification or Interruption....45
10.3.2. Cetuximab Dose Modification or Interruption 46
10.3.2.1. Allergic/hypersensitivity Reactions....46
10.3.2.2. Skin Toxicity 46
10.3.2.3. Additional Cetuximab Considerations....47
10.4. Prior/Concomitant Medications....47
10.4.1. Permitted Concomitant Therapy....47

10.4.2. Prohibited Concomitant Therapy....48
10.5. Treatment Compliance....48
10.6. Discontinuation from Treatment....48
11. INVESTIGATIONAL PRODUCT MATERIALS AND MANAGEMENT....49
11.1. Supplier(s)....49
11.2. Dosage Form....49
11.3. Dosage Regimen....49
11.4. Investigational Product Packaging and Labeling....49
11.5. Investigational Product Receipt and Storage....49
11.6. Record of Administration....50
11.7. Investigational Product Accountability....50
11.8. Investigational Product Handling and Disposal....50
12. ASSESSMENT OF EFFICACY....51
12.1. Assessments....51
12.2. Methods and Timing of Efficacy Assessments....51
12.2.1. Tumor Assessment....51
12.2.2. Response Assessment....51
12.2.3. ECOG Performance Status....51
12.2.4. Survival....51
13. ASSESSMENT OF SAFETY....52
13.1. Assessments....52
13.2. Methods and Timing of Safety Assessments....52
13.2.1. Adverse Event Reporting....52
13.2.2. Concomitant Medications....52
13.2.3. Physical Examinations....52
13.2.4. Vital Signs....52
13.2.5. Hematology Laboratory Assessments....52
13.2.6. Chemistry Laboratory Assessments....53
13.2.7. Pregnancy Testing....53
13.2.8. Thyroid Function Test....53
14. OTHER ASSESSMENTS....54
14.1. Biomarker Assessment....54

14.1.1. Preparation/Handling/Shipping of Tumor and Blood Samples for Biomarker Assessment 54
14.1.1.1. Plasma 54
14.1.1.2. Blood for Circulating Tumor Cells 55
14.1.1.3. Blood for Immuno Flow Cytometry 55
14.1.1.4. Paraffin Blocks or Slides 55
14.1.2. Sample Storage and Destruction 55
14.1.3. Confidentiality 55
15. STATISTICAL CONSIDERATIONS 56
15.1. Study Population Definitions 56
15.2. Efficacy Evaluation 56
15.3. Background and Demographic Characteristics 56
15.4. Investigational product(s) 57
15.5. Safety Evaluation 57
15.6. Interim Analyses 57
15.7. Sample Size and Power Considerations 57
16. QUALITY CONTROL AND QUALITY ASSURANCE 58
16.1. Monitoring 58
16.2. Audits and Inspections 58
16.3. Investigator(s) Responsibilities 58
17. REGULATORY CONSIDERATIONS 60
17.1. Institutional Review Board/Independent Ethics Committee Review and Approval 60
17.2. Protocol Amendments 60
17.3. Informed Consent 60
17.4. Subject Confidentiality 61
18. DATA HANDLING AND RECORDKEEPING 62
18.1. Data/Documents 62
18.2. Data Management 62
18.3. Retention of Records 62
19. PREMATURE DISCONTINUATION OF THE STUDY 64
19.1. Single Site 64
19.2. Study as a Whole 64

20. REFERENCES 65
21. APPENDICES 68
21.1. ECOG Performance Status Scale (Oken, 1982) 68
21.2. Adverse Event..69
21.3. Lenalidomide Pregnancy Risk Minimisation Plan for Celgene Clinical Trials (Ex-EU territories)..74
21.3.1. Lenalidomide Risks of Fetal Exposure, Pregnancy Testing Guidelines and Acceptable Birth Control Methods..75
21.3.2. Lenalidomide Education and Counselling Guidance Document.............................79
21.3.3. Lenalidomide Patient Card 82
21.3.4. Lenalidomide Information Sheet 83
21.4. Lenalidomide Pregnancy Prevention Risk Management Plan (Europe) 86
21.4.1. Lenalidomide Risks of Fetal Exposure, Pregnancy Testing Guidelines and Acceptable Birth Control Methods (Europe)...87
21.4.2. Lenalidomide Education and Counseling Guidance Document (Europe).................90
21.4.3. Lenalidomide Patient Card (Europe) 92
21.4.4. Lenalidomide Information Sheet (Europe)...93
21.5. Declaration of Helsinki...97

LIST OF TABLES

Table 1: Celgene Corporation Emergency Contact Information 4

Table 2: Table of Assessments 14

Table 3: Abbreviations and Special Terms 25

Table 4: Response Rates and KRAS Mutational Status in Subjects Treated With Anti-EGFR Containing Regimens 30

Table 5: Schedule of Lenalidomide Safety Lead-In Dosing 39

Table 6: Dose Modification Guidelines for Lenalidomide 45

Table 7: Treatment Adjustment for Cetuximab-Related Allergic/Hypersensitivity Reactions....................................46

LIST OF FIGURES

Figure 1 Study Design Schema 17
Figure 2: Lenalidomide enhances cetuximab mediated ADCC independendly of KRAS status....31
Figure 3: Treatment Adjustments for Cetuximab-Related Skin Toxicity....47

4. LIST OF ABBREVIATIONS AND DEFINITIONS OF TERMS

Table 3: Abbreviations and Special Terms

Abbreviation or Specialist Term	Explanation
AE	Adverse event
ALT (SGPT)	Alanine transaminase (serum glutamic- pyruvic transaminase)
ANC	Absolute neutrophil count
ADCC	Antibody-dependent cellular cytotoxicity
AST (SGOT)	Aspartate transaminase (serum glutamic-oxaloacetic transaminase)
bFGF	Basic fibroblast growth factor
β-HCG	Beta human chorionic gonadotropin
BUN	Blood urea nitrogen
CBC	Complete blood count
CFR	Code of Federal Regulations
CR	Complete response
CRC	Colorectal cancer
CRF	Case report form
CT	Computerized axial tomography
CTCs	Circulating Tumor Cells
CTCAE	Common terminology criteria for adverse events
DCF	Data clarification form
DCR	Disease control rate
DLT	Dose Limiting Toxicity
ECOG	Eastern Cooperative Oncology Group
EGFR	Epidermal growth factor receptor
EMEA	European medicines agency
FDA	Food and Drug Administration
FGF	Fibroblast growth factor
GCP	Good Clinical Practice
GM-CSF	Granulocyte Macrophage-Colony Stimulating Factor
HCG	Human chorionic gonadatropin
HRQOL	Health-Related Quality of Life

Table 3: Abbreviations and Special Terms (Continued)

Abbreviation or Specialist Term	Explanation
ICF	Informed consent
ICH	International Conference on Harmonization
IEC	Independent Ethics Committee
IFN	Interferon
IgG	Immunoglobulin G
IMiDs	Immunomodulatory Drugs
IND	Investigational New Drug
IRB	Institutional Review Board
ITT	Intent to treat
IV	Intravenously
KRAS	V-Ki-ras2 Kirsten rat sarcoma viral oncogene homolog
LDH	Lactate dehydrogenase
LPS	Lipopolysaccharide
MM	Multiple Myeloma
MRI	Magnetic resonance imaging
MTD	Maximum Tolerated Dose
NCCN	National Comprehensive Cancer Network
NCI	National Cancer Institute
NHL	Non-Hodgkin lymphoma
NK	Natural killer
OS	Overall survival
PBMC	Peripheral blood mononuclear cell
PD	Progressive disease
PDGFR	P-derived growth factor receptor
PET	Positron emission tomography
PGF	Placental growth factor
PFS	Progression-free survival
PO	Orally
POC	Proof of concept

Table 3: Abbreviations and Special Terms (Continued)

Abbreviation or Specialist Term	Explanation
PR	Partial response
RBC	Red blood cell
RECIST	Response Evaluation Criteria in Solid Tumors
SAE	Serious adverse event
SCID	Severe combined immunodeficiency
SD	Stable disease
SOP	Standard Operating Procedure
TNF	Tumor necrosis factor
TPP	Therapeutic Product Programme
TSH	Thyroid stimulation factor
ULN	Upper limit of normal
VEGF	Vascular endothelial growth factor
VEGFR	Vascular endothelial growth factor receptor
WBC	White blood cells
WCBP	Women of child bearing potential

5. INTRODUCTION

5.1. Colorectal Cancer

Colorectal cancer (CRC) is the fourth most common cancer in men and the third in women. Worldwide, nearly 1.2 million cases of colorectal cancer were expected to occur in 2007. The highest incidence rates are found in Japan, North America, parts of Europe, New Zealand, and Australia. Rates are low in Africa and South-East Asia. Rates are substantially higher in men than in women. More than 300,000 new patients are diagnosed each year with CRC in the European Union and approximately 150,000 new cases of colorectal cancer were diagnosed in the United States in 2008. Colorectal cancer is the second cause of cancer death both in the United States and in the European Union. About 630,000 deaths from colorectal cancer were expected to occur in 2007 worldwide, accounting for 8% of all cancer deaths (Global Cancer Facts and Figures, 2007; National Comprehensive Cancer Network Guidelines, 2008). Mortality from colon cancer has decreased slightly over the past 30 years; however, a need remains for more effective treatments.

5.2. Background and Rationale

5.2.1. Treatment of Colorectal Cancer

The therapy of colon cancer depends on the stage at the time of diagnosis. For stage I and II colon cancers, surgery with wide resection margins is the only therapy needed, although some stage II patients receive chemotherapy. For stage III disease, adjuvant 5-fluorouracil (5-FU) + leucovorin (LV) or 5-FU + LV + oxaliplatin (FOLFOX) for 6 months is standard and has been shown to improve survival (Carethers, 2008).

Treatment in patients with metastatic CRC (mCRC) focuses on changing the chemotherapy regimens after progression of adjuvant therapy. First-line patients, depending on their adjuvant treatment, usually get treated either with FOLFOX +/- bevacizumab or 5-FU + LV + irinotecan (FOLFIRI) +/- bevacizumab, and to a lesser degree with capecitabine + oxaliplatin (XELOX) +/- bevacizumab or capecitabine + irinotecan (XELIRI) +/- bevacizumab. Until a few years ago, treatment options for patients with mCRC were limited to 5-FU, with overall response rates (ORRs) of 10%-15% and a median overall survival (OS) of 10 months. The addition of irinotecan or oxaliplatin to 5-FU + LV has increased the median OS to 16 months and the ORR to approximately 50%. With the introduction of bevacizumab into the combination regimens in first-line, median OS was further increased to above 20 months (Wolpin, 2008).

Once first-line patients progress, the first line treatment administered will determine the second-line treatment to be used. If the patient was treated with FOLFIRI +/- bevacizumab as first-line, they will usually be treated with FOLFOX +/- bevacizumab; if the patient was treated with FOLFOX +/- bevacizumab as first-line, they will usually be treated with FOLFIRI +/- bevacizumab and in some cases with FOLFIRI +/- cetuximab. Cetuximab containing regimens are used only in KRAS wild-type patients in the EU. In some cases, second-line patients will also be treated with capecitabine +/- bevacizumab.

Once these patients progress, third-line treatment in the EU will depend mostly on the KRAS status of the patient. Treatment of KRAS wild-type patients usually consists in third-line of either FOLFIRI +/- cetuximab or irinotecan +/- cetuximab, and in a small number of patients with cetuximab or panitumumab monotherapy. Third-line therapy for KRAS mutant patients is very limited and depends on patient characteristics and physician's preference. The only therapeutic option in alternative to best supportive care (BSC) is the enrollment in clinical trials (Peeters, 2009).

5.2.2. Treatment with Anti-EGFR Therapies and KRAS Gene Mutation Status

The epidermal growth factor receptor (EGFR) is a transmembrane glycoprotein that interacts with signaling pathways affecting cellular growth, proliferation, and programmed cell death and is expressed in malignancies of multiple tissues, including those of the colon, lung, breast, and head and neck. In colorectal cancer, EGFR expression on the tumor cell surface has been shown in up to 80% of tumors (Messa, 1998; Porebska, 2000) and tumors that express EGFR carry a poorer prognosis (Mayer, 1993). Antibodies directed against the extracellular domain of EGFR have been developed to inhibit the function of this transmembrane receptor. Thus far, the anti-EGFR monoclonal antibodies, cetuximab (chimeric IgG1 monoclonal antibody) and panitumumab (fully human IgG2 monoclonal antibody), have shown efficacy in colorectal cancer.

5.2.2.1. Impact of KRAS Mutational Status on Anti-EGFR Therapies

RAS proteins are membrane-localized G proteins that function as signal switches that link the activation of EGFR to downstream effectors involved in cell differentiation and proliferation. Mutations in the genes encoding the RAS proteins usually lead to constitutive activation of the downstream signaling pathways and are implicated in the development of colorectal cancer. For example, KRAS gene mutations are found in approximately 40% of CRC patients with at least one mutation in exon 2 (Karapetis, 2008).

The impact of KRAS mutational status on the treatment of mCRC with anti-EGFR therapies has been analyzed through retrospective analyses of several mCRC clinical studies. Table 4 summarizes the results of these analyses in which KRAS mutational status was studied in patients treated with anti-EGFR therapies. Ninety-five percent confidence intervals are calculated based on exact binomial distribution. The difference appears evident that KRAS mutational status differentiates tumor response to anti-EGFR containing regimens since lower limits of the response rates observed in the KRAS wild type group are much higher than the upper limits in the KRAS mutant group. As the accuracy of response rate assessment may be limited due to individual study size, multiple studies are pooled in a meta analysis. In tumors harboring KRAS mutations, the 95% confidence interval of response rate ranges from 0 % to 2 %, indicating practically and absence of anti-tumor activity.

Table 4: Response Rates and KRAS Mutational Status in Subjects Treated With Anti-EGFR Containing Regimens

Study	Treatment	Number of patients (MT:WT)	RR in Mutant	95% CI–LL	95% CI-UP	RR in Wild type	95% CI-LL	95% CI-UP
De Roock, 2008	Cetuximab +/- irinotecan	113 (46:67)	0	0%	6%	27 (40%)	29%	53%
Finocchiaro, 2007	Cetuximab +/- irinotecan	81 (32:49)	2 (6%)	1%	21%	13 (27%)	15%	41%
Khambata-Ford, 2007	Cetuximab	80 (30:50)	0	0%	10%	5 (10%)	3%	22%
Lievre, 2008	Cetuximab +/- chemo	89 (24:65)	0	0%	12%	26 (40%)	28%	53%
Karapetis, 2008	Cetuximab	198 (81:117)	1 (1%)	0%	7%	13 (11%)	6%	18%
Hecht, 2008	Panitumumab	171 (77:94)	0	0%	4%	8 (9%)	4%	16%
Freeman, 2008	Panitumumab	59 (21:38)	0	0%	13%	6 (16%)	6%	31%
Amado, 2008	Panitumumab	208 (84:124)	0	0%	4%	21 (17%)	11%	25%
Pooled		999 (395:604)	3 (1%)	0%	2%	119 (20%)	17%	24%

In the European Union, the indication of cetuximab and panitumumab has been restricted to patients with tumors not harboring activating KRAS mutations.

5.2.3. Lenalidomide

Lenalidomide modifies or regulates the functioning of the immune system. It may affect the immune system in several ways leading to protective anti-tumor immune responses. These include costimulation of T cells and NK cells, leading to the enhanced potential for antibody-dependent cellular cytotoxicity (ADCC), and inhibition of the expansion and function of T regulatory cells (Galustian, 2008; Bartlett, 2004). Although lenalidomide's precise mechanism of action is currently under investigation, it offers promise for their anti-cancer properties in particular in combination with therapeutic antibodies.

Lenalidomide is an analog of thalidomide with the chemical name, alpha-(3-aminophthalimido) glutarimide (Mitsiades, 2004). However, whereas lenalidomide is able to enhance ADCC in vitro, an effect of thalidomide is has not been observed even at high concentrations. Furthermore, lenalidomide retains anti-angiogenic activity in vitro.

Combination and single agent studies with lenalidomide have demonstrated activity and tolerability in solid tumors during Phase 1 and Phase 2 studies. Lenalidomide has been tolerated up to 35 mg in solid tumor patients when administered during the first 21 days of 28-day cycles (Tohnya, 2006).

5.2.4. Lenalidomide Nonclinical Findings

Lenalidomide has been assessed for pharmacological activity in a variety of preclinical models and has shown immunomodulatory and anti-angiogenic activities in vitro and in vivo.

5.2.4.1. Immunomodulatory Activity of Lenalidomide

Lenalidomide enhances NK cell and monocyte mediated antibody-dependent cellular cytotoxicity of rituximab against a variety of hematological cell lines in vitro, including, NHL and B-CLL (Wu, 2008). Lenalidomide also enhances NK cell mediated lysis of cetuximab and trastuzumab coated colorectal and breast cancer cells respectively (Wu, 2008). This effect is mediated by enhanced granzyme B expression. The ability of lenalidomide to enhance cetuximab mediated ADCC of CRC cells is not affected by the KRAS mutational status. Thus, KRAS wild type and KRAS mutant CRC cells are equally sensitive to enhancement of ADCC by lenalidomide (Wu, 2008). This is as expected since NK cells recognize the surface bound antibody and are able to kill tumor cells independently of EGFR pathway activation. Because panitumumab, an IgG2a EGFR mAb does not effectively interact with FCγ receptors on the NK cell surface it is unable to initiate ADCC and as expected lenalidomide has no effect since its activity is reliant on the augmentation of NK cell signaling downstream of FCγR.

Figure 2: Lenalidomide enhances cetuximab mediated ADCC independendly of KRAS status

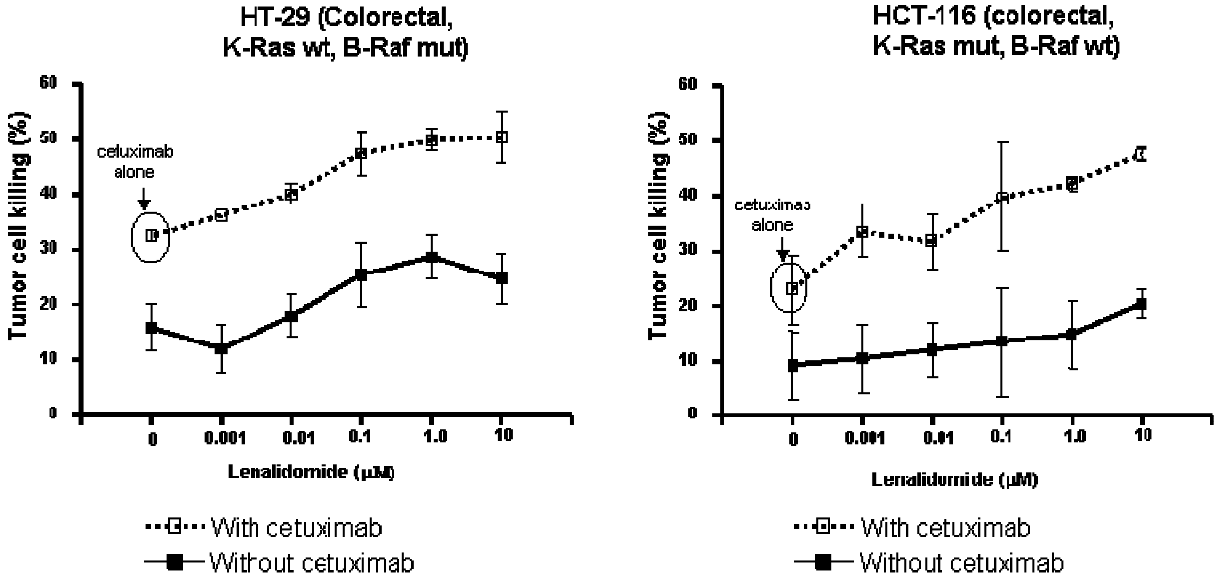

(Wu, 2008)

Further evidence for the ability of lenalidomide to enhance ADCC was shown when lenalidomide synergized with rituximab to promote animal survival in a Raji (Non-Hodgkin's

Lymphoma, NHL) SCID mouse model (Hernandez-Ilizaliturri, 2005). The augmentation of antitumor activity that occurred when lenalidomide was used in combination with rituximab was the result of enhanced innate immunity mediated by IL-2-stimulated NK cell expansion, and activation and trafficking of the NK cells into the tumor bed promoting ADCC (Reddy, 2008).

Lenalidomide is a T cell co-stimulator and enhances the proliferation and production of IL-2 and IFN-γ by T cells in response to αCD3 stimulation. In a PBMC/tumor cell co-culture model T cell co-stimulation leads to enhanced NK cell-mediated lysis of various tumor cells, including prostate and ovarian cancer cells (Zhu, 2008). Enhanced NK cell function is reliant on T cell derived cytokines. These results suggest that tumor specific immune responses can be enhanced by lenalidomide.

Lenalidomide also appears capable of repairing F-actin polymerization and signaling at the immunological synapse between T cells and B-Chronic Lymphocytic Leukemia (CLL) cells, providing further evidence for the ability of lenalidomide to enhance productive immunological interactions with tumor cells. Similar immune synapse formation defects were observed in Follicular Lymphoma (FL) and Diffuse Large B-Cell Lymphoma (DLBCL) cells, although synapse formation between T cells and solid tumor cells has yet to be investigated (Ramsay, 2008).

Furthermore, lenalidomide has been shown to enhance CD8+ T cell effector function in an in vitro dendritic cell (DC)/CD8+ T cell coculture system. Specifically, lenalidomide enhanced virus-specific CD8+ T cell cytokine production and cytotoxic activity in cells from healthy donors as well as cells from subjects chronically co-infected with HIV and cytomegalovirus (Haslett, 2003). This data further supports the ability of lenalidomide to augment immune responses.

Enhancement of vaccine efficacy may be in part due to the ability of lenalidomide to inhibit the IL-2-mediated expansion of T regulatory cells (Tregs) and also their ability to suppress the proliferation of autologous anti-CD3 stimulated T cells (Galustian, 2008). The ability to inhibit Treg cell function may help enable tumor-specific immune responses in patients with cancer.

Evidence for the ability of lenalidomide to enhance immune activity was seen in patients with solid tumors treated with lenalidomide. In this study, enhanced serum GM-CSF, TNF-α and IL-12 levels were associated with the elevation of sIL-2Rα and also a shift towards a circulating T cell “activated/memory” phenotype (Bartlett, 2004).

Evidence for immune enhancing activity in patients is indicated by data showing that lenalidomide treatment of patients with multiple myeloma (MM) enhances the immunological response to the pneumoccocal vaccine prevnar, in particular when given in advance of prevnar administration. This data indicates a potential for lenalidomide to enhance vaccine efficacy in cancer patients (Noonan, 2008).

5.2.4.2. Anti-angiogenic and Anti-metastatic Activity of Lenalidomide

In a human tissue culture model of angiogenesis, lenalidomide inhibited the formation of microvessels emanating from an arterial explant in a dose-dependent manner (Lu, 2008). Additionally, Lenalidomide inhibits Endothelial cell migration in response to a variety of growth factors, including VEGF and bFGF. This effect is associated with the inhibition of VEGF and bFGF induced phosphorylation of Akt (Dredge, 2005).

Lenalidomide is anti-angiogenic in the CMT-93 colorectal tumor model (Dredge, 2002). Orally administered lenalidomide was found to be anti-angiogenic in the rat mesenteric window model of in vivo angiogenesis (Dredge, 2005). Lenalidomide has also been shown to be anti-metastatic in the B16-F10 melanoma and CT26 colorectal cancer models (Lu, 2008; Wai, 2008).

5.2.5. Lenalidomide Dosing and Schedule Rationale

The current plan is to have a Safety Lead-In part of the study with lenalidomide in combination with cetuximab to establish the MTD with the combination. The stating dose of lenalidomide for the Safety Lead-In part is proposed to be 25 mg daily due to the clinical data with lenalidomide in other indications. Currently lenalidomide is approved as a treatment for MDS at a dose of 10 mg daily and for treatment of MM at a dose of 25 mg once each day for days 1-21 of a 28 day cycle. While myelosuppression is the major dose-limiting toxicity in patients with hematologic disorders who are taking lenalidomide treatment, evidence exists that lenalidomide tolerability may be improved for patients without hematological malignancies or compromised bone marrow. For example, in a dose escalation study entitled, “Phase I study of lenalidomide, a novel thalidomide analog, in patients with refractory metastatic cancer,” Tohnya et al enrolled 45 patients with refractory, metastatic solid tumors. Patients were enrolled to receive single agent lenalidomide 5 to 40 mg administered on days 1-21 of each 28-day treatment cycle. Lenalidomide was well-tolerated up to 35 mg. Within the study, the following grade 3 toxicities were seen: diarrhea (1), hypotension (1), pulmonary effusions (1), and thrombosis (2). Grade 4 adverse events included 2 patients with neutropenia, one patient with arrhythmia, and a single patient with hemolysis (Tohnya, 2006). It was suggested that lenalidomide may have a lower incidence and severity of myelosuppression in solid tumors compared to myelosuppression observed in patients with hematologic malignancies or compromised bone marrow. Lenalidomide studies performed in prostate cancer patients have found that both as single agent and in combination with other agents including docetaxel, lenalidomide is well-tolerated at doses of at least 25 mg or higher in some cases (Petrylak, 2009; Sanborn, 2008).

Lenalidomide is proposed to be administered continuously on Days 1-28 of each 28-day cycle to potentially maximize the combined ADCC effects of the two drug combination.

The Phase 2b – Proof of Concept (POC) will use the combination dose established from the Safety Lead-In part and lenalidomide 25 mg as a single agent dose. The Phase 2b – Expansion part will use the combination dose established from the Safety Lead-In part.

5.2.6. Study Rationale

Colorectal cancer therapy has developed extensively over the last 10 years. Patients were initially being treated with single agent 5-FU only, and now they have access to multiple lines of therapy which significantly increased overall survival through the introduction of 5-FU based chemotherapeutic regimens plus the introduction of novel treatment modalities such as anti-angiogenesis and EGFR inhibition.

Even though an increase in overall survival has been achieved through the use of these novel therapies, there is still a high unmet clinical need in patients that progress through the different lines of therapy and become resistant to the available drugs. The recent understanding of the impact of gene point mutations affecting the EGFR signaling pathways helped to define

subgroups of patient populations that lack proven treatment alternatives. One such population consists of patients that harbor point mutations on the KRAS gene which results in the constitutive activation of the EGFR signaling pathway driving the cell into mitosis.

Lenalidomide has the potential for clinical activity in solid tumors due to a variety of mechanistic properties that may lead to cancer growth inhibition including, immune modulation, anti-angiogenesis and effects within the tumor microenvironment. Evidence that lenalidomide is a potent immunomodulator with anti-angiogenic activity provides the non-clinical rationale for evaluating lenalidomide in colorectal cancer. Based on preclinical and in vitro studies highlighted above, Celgene Corporation expects that lenalidomide in combination with cetuximab will enhance T cell and NK cell activation leading to enhanced NK cell-mediated ADCC of colorectal tumor cells, regardless of KRAS mutational status and subsequent clinical benefit for CRC patients.

6. STUDY OBJECTIVES

6.1. Primary

6.1.1. Phase 2a

- To determine the Maximal Tolerated Dose (MTD) of lenalidomide in combination with cetuximab in subjects with KRAS mutant metastatic CRC.

6.1.2. Phase 2b

- To determine the response rate in subjects with KRAS mutant metastatic CRC.

6.2. Secondary (all phases)

- To evaluate the safety and tolerability in subjects with KRAS mutant metastatic CRC.
- To assess the clinical efficacy in subjects with KRAS mutant metastatic CRC.

6.3. Exploratory (all phases)

- Analysis of biomarkers as measures for validation of clinical efficacy and toxicity.

7. STUDY ENDPOINTS

7.1. Primary

7.1.1. Phase 2a

- Maximum Tolerated Dose

7.1.2. Phase 2b

- Response Rate according to Response Evaluation Criteria in Solid Tumors 1.1 (RECIST 1.1) (Eisenhauer, 2009).

7.2. Secondary

- Progression Free Survival.
- Safety / Tolerability (type, frequency, severity, and relationship of adverse events to investigational products).
- Duration of Response.
- Disease Control Rate.
- Overall Survival.

7.3. Exploratory

- Assessment of biomarkers including, but not limited to:
  - Immuno flow cytometry panel for T cells and NK cells.
  - Cytokines/immune markers.
  - Growth factors.
  - Circulating tumor cells.
  - FCgR polymorphisms.
  - EGFR copy number.

8. OVERALL STUDY DESIGN

This is a Phase 2, multicenter, open-label study of lenalidomide in combination with cetuximab in subjects with KRAS mutant metastatic CRC. The primary objective of the study is to determine the MTD in Phase 2a and response rate in Phase 2b. The secondary objectives are to evaluate the safety, tolerability, and efficacy.

Subjects will be screened within 28 days of study entry as outlined in Table 2. Up to approximately 220 subjects meeting all eligibility criteria will be enrolled into this 3-part study. Each of the three parts of this study; Phase 2a - Safety Lead-In, Phase 2b - POC, and Phase 2b - Expansion, are described below. Laboratory testing will be performed by a central laboratory for this study.

Subjects will continue on study treatment until documented disease progression per RECIST 1.1, unacceptable toxicity, death, or treatment discontinuation for any other reason. Subjects should be provided with appropriate supportive care throughout the study.

8.1. Phase 2a – Safety Lead-In

Approximately 6-18 subjects meeting all eligibility criteria will be enrolled by IVRS into the Phase 2a – Safety Lead-In part receiving treatment with lenalidomide in combination with cetuximab to establish the MTD with the combination.

8.1.1. Safety Lead-In Dose Finding

Initially 6 subjects will be enrolled and treated with a daily oral dose of lenalidomide at 25 mg administered on Days 1-28 of each 28-day cycle and intravenous (IV) infusions of cetuximab (400 mg/m^2 first infusion only, then 250 mg/m^2 subsequently) administered on Days 1, 8, 15, and 22 of each 28-day cycle.

- If < 2 of the 6 subjects experience a Dose Limiting Toxicity (DLT), then the Phase 2b – POC part will start with lenalidomide at 25 mg.
- If ≥ 2 of the 6 subjects experience a DLT, then 6 additional subjects will be enrolled at lenalidomide 20 mg.
  - If < 2 of the additional 6 subjects experience a DLT, then the Phase 2b – POC part will start with lenalidomide at 20 mg.
  - If ≥ 2 of the additional 6 subjects experience a DLT, then 6 additional subjects will be enrolled at lenalidomide 15 mg.
    - If < 2 of the additional 6 subjects experience a DLT, then the Phase 2b – POC part will start with lenalidomide at 15 mg
    - If ≥ 2 of the additional 6 subjects experience a DLT, then Celgene Corporation will discuss with the investigators and reassess the doses.

Dosing does not escalate during the safety lead-in part of the study. In addition, there will be no intra-patient dose escalation. Cycles will be repeated every 28 days. All subjects will continue on

investigational product(s) until documented tumor progression, unacceptable toxicity, death, or treatment discontinuation for any other reason.

8.1.2. Safety Lead-In Definition of Dose Limiting Toxicity

DLT is defined as one or more of the following:

- Total of ≥ 7 days of lenalidomide and/or ≥ 1 dose of cetuximab missed during the first cycle due to drug-related toxicity as outlined below:
  - Any Grade 3 or 4 non-hematological toxicity (excluding alopecia), even after optimally treated with supportive measures.
    - A rash with suspected relationship to cetuximab, treated and resolved in accordance with the guidelines will not be considered a DLT.
  - Grade 4 neutropenia or febrile neutropenia.
  - Grade 4 thrombocytopenia.

Should any subject discontinue the study prior to completing the entire first cycle for reasons other than a DLT or if ≥ 7 days of lenalidomide and/or ≥ 1 dose of cetuximab were missed during the first cycle for reasons other than a DLT, then a replacement subject will be added at that dose level.

The occurrence of one of the above drug-related toxicities will result in a clinical and/or laboratory assessment to be performed within 7 days following the initial finding to examine the subject for resolution of the toxicity. Lack of resolution of any of these toxicities according to the guidelines above or within 7 days of scheduled start of Cycle 2 Day 1 will be considered a DLT. Subjects who experience a DLT may continue on treatment at the Investigator's discretion at a lower dose of lenalidomide in accordance with Table 6.

Adverse events will be graded using the CTCAE Version 4.0 (http://ctep.cancer.gov/protocolDevelopment/electronic_applications/docs/ctcaev4.pdf).

If cetuximab is discontinued, lenalidomide may be continued until documented tumor progression, unacceptable toxicity, death, or treatment discontinuation for any other reason.

Following Cycle 1, all subjects will continue on assigned treatment until documented tumor progression, unacceptable toxicity, death, or treatment discontinuation for any other reason and be followed for toxicity and secondary endpoints.

8.1.3. Safety Lead-In Definition of MTD

The MTD of lenalidomide will be defined as the highest dose level at which no more than 1 out of 6 subjects experience a DLT during Cycle 1 of lenalidomide in combination with cetuximab.

Safety measurements and analysis will be performed at each visit as outlined in Table 2.

Table 5: Schedule of Lenalidomide Safety Lead-In Dosing

Dose Level	Lenalidomide Dose (PO) Days 1-28 of a 28-Day Cycle	Cetuximab Dose (IV) Day 1, 8, 15 & 22 of each 28-Day Cycle
**1 Initial Dose Level**	25 mg	400 mg/m^2 initial infusion only, then 250 mg/m^2 subsequently
2	20 mg	
3	15 mg	

8.2. Phase 2b – Proof of Concept

Once dosing has been established in the Safety Lead-in part, POC will be established with up to approximately 82 additional subjects enrolled to receive oral lenalidomide in combination with cetuximab (at the MTD determined during the Safety Lead-in part of the study) or as a single agent. Subjects meeting all eligibility criteria will be enrolled into one of the following two treatment arms and randomized 1:1 ratio by IVRS:

- Arm 1 (up to 41 subjects): Daily oral lenalidomide (dose determined from Safety Lead-in part) on Days 1-28 plus IV infusions of cetuximab (400 mg/m^2 initial infusion only, then 250 mg/m^2 subsequently) administered on Days 1, 8, 15, and 22 of each 28-day cycle.
- Arm 2 (up to 41 subjects): Daily oral lenalidomide 25 mg on Days 1-28 of each 28-day cycle.

Study treatment will continue until documented tumor progression per RECIST 1.1, unacceptable toxicity, death, or treatment discontinuation for any other reason.

The study may proceed to Phase 2b – Expansion part if the response rate from either arm in the Phase 2b – POC part is deemed significantly higher than 10%.

Conventional (or spiral) CT/MRI imaging is required. To ensure comparability, baseline methods and on-study methods for tumor assessment must be performed using identical techniques.

Study treatment will continue until documented tumor progression per RECIST 1.1, unacceptable toxicity, death, or treatment discontinuation for any other reason.

8.3. Phase 2b – Expansion

Subjects meeting all eligibility criteria will be enrolled by IVRS to take oral lenalidomide (dose determined from Safety Lead-in part) on Days 1-28 plus IV infusions of cetuximab (400 mg/m^2 initial infusion, then 250 mg/m^2 subsequently) administered on Days 1, 8, 15, and 22 of each 28-day cycle. Celgene Corporation will reassess the feasibility of adding a single agent lenalidomide arm to the Phase 2b – Expansion part of the study depending on results from the Phase 2b – POC part.

Study treatment will continue until documented tumor progression, unacceptable toxicity, death, or treatment discontinuation for any other reason.

8.4. Follow-Up Period

All study subjects who discontinue from the study for any reason other than withdrawal of consent will enter the Follow-up period that includes one follow-up visit 28 days after last dose and subsequent telephone contacts every 90 days until death or 5 years post-discontinuation.

8.5. Duration of Treatment

Subjects will be screened within 28 days of study treatment initiation. Study treatment will continue until subjects experience disease progression, unacceptable toxicity or treatment discontinuation for any other reason.

9. STUDY POPULATION

9.1. Subject Inclusion Criteria

Subjects must meet all of the following inclusion criteria to be eligible for enrollment into the study:

9.1.1. Disease Characteristics

1. Histologically or cytologically confirmed metastatic colorectal adenocarcinoma.
2. At least one unidimensionally measurable lesion.
3. Documented KRAS mutant tumor (paraffin tumor blocks or approximately 25 unstained slides must be sent for KRAS mutation testing and results reported by central pathology laboratory prior to study enrollment).
4. Imaging confirmed (CT/MRI) disease progression within 42 days of previous treatment.
5. Disease progression on oxaliplatin- AND irinotecan-containing regimens in the metastatic setting, with at least one of these regimens containing bevacizumab.

9.1.2. ECOG

6. Eastern Cooperative Oncology Group performance status of ≤ 1.

9.1.3. Pregnancy

7. Women of childbearing potential must undergo pregnancy testing based on the frequency outlined in Section 21.3 or 21.4 and pregnancy results must be negative.
8. Unless practicing complete abstinence from heterosexual intercourse, sexually active WCBP must agree to use adequate contraceptive methods as specified in Section 21.3 or 21.4.
9. Males (including those who have had a vasectomy) must use barrier contraception (condom) when engaging in sexual activity with WCBP as specified in Section 21.3 or 21.4.
10. Males must agree not to donate semen or sperm during the duration specified in Section 21.3 or 21.4.
11. All subjects must:
    - Understand that the investigational product could have a potential teratogenic risk.
    - Agree to abstain from donating blood while taking investigational product and following discontinuation of investigational product (see Section 21.3 or 21.4).
    - Agree not to share study medication with another person.
    - Be counseled about pregnancy precautions and risks of fetal exposure (see Section 21.3 or 21.4).

9.1.4. General

12. Understand and voluntarily sign an ICF.
13. Age ≥ 18 years at the time of signing the ICF.
14. Able to adhere to the study visit schedule and other protocol requirements.

9.2. Subject Exclusion Criteria

Subjects must not have met any of the following exclusion criteria to be eligible for enrollment into the study:

9.2.1. Prior Treatment

1. Use of chemotherapy, hormonal therapy, immunotherapy, or any other anticancer or experimental therapy ≤ 28 days prior to Cycle 1 Day 1.
2. Radiotherapy to ≥ 30% of the bone marrow.
3. Surgery ≤ 28 days prior to Cycle 1 Day 1 (minimally invasive procedures for the purpose of diagnosis or staging of the disease are permitted).
4. Prior therapy with cetuximab.
5. Prior therapy with panitumumab.
6. Prior therapy with pomalidomide (CC-4047), lenalidomide, or thalidomide.

9.2.2. Laboratory

7. Absolute neutrophil count (ANC) < 1.5 x 10^9/L.
8. Platelet count < 100 x 10^9/L.
9. Creatinine Clearance < 50 mL/min (by Cockroft- Gault).
10. Bilirubin > 1.5 x ULN (> 2.0 x ULN in the presence of Gilbert's Syndrome).
11. Serum AST or ALT > 3.0 x ULN (> 5 x ULN in the presence of liver metastases).
12. Hemoglobin < 9 g/dL.

9.2.3. Other Disease State

13. Untreated, symptomatic brain metastases (brain imaging not required).
14. Venous thromboembolism ≤ 6 months prior to Cycle 1 Day 1.
15. Current congestive heart failure (New York Heart Association class II-IV).
16. Myocardial Infarction (MI) ≤ 12 months prior to Cycle 1 Day 1.
17. Uncontrolled hypertension.
18. Prior malignancies within 5 years, with the exception of treated basal cell/squamous cell carcinoma of the skin or "in-situ" carcinoma of the cervix or breast.

9.2.4. General

19. Any condition (psychological, familial, sociological or geographical), including the presence of laboratory abnormalities, which would place the subject at unacceptable risk if he/she were to participate in the study, confound the ability to interpret data from the study, or not allow the subject to comply with the requirements of the study protocol.
20. Pregnant or lactating women.

10. DESCRIPTION OF TREATMENT

10.1. Study Treatment

10.1.1. Lenalidomide

Celgene Corporation will supply lenalidomide as 5, 10, 15, 20, and 25 mg capsules to be administered PO once daily for Days 1-28 of each 28-day treatment cycle. Study drug will be packaged in bottles containing enough capsules for 28 days of dosing. All subjects will be required to take a single capsule each day for Days 1-28 of each cycle. Lenalidomide should be taken at approximately the same time each day.

10.1.2. Cetuximab

Celgene Corporation will supply commercial cetuximab labeled as IMP for administration via IV infusion on Days 1, 8, 15, and 22 of each 28-day cycle. Cetuximab will be administered at 400 mg/m^2 initial dose, then 250 mg/m^2 weekly.

10.2. Treatment Assignments

Subjects meeting all eligibility criteria will be enrolled into the study via IVRS. Dose levels will be determined according to Section 8. Treatment with investigational products will continue in 28-day cycles until disease progression, unacceptable toxicity or treatment discontinuation for any other reason.

10.3. Dose Modification or Interruption

Dose modifications of lenalidomide and cetuximab are not permitted during Cycle 1 of the Phase 2a – Safety Lead-In part, unless a subject experiences a DLT, however permitted anytime after Cycle 1 during Phase 2a – Safety Lead-In, anytime during Phase 2b –POC and Phase 2b – Expansion.

For toxicities consistent with cetuximab administration, discontinuation, dose interruptions and/or reductions see Section 10.3.2 and the approved product labeling. If toxicities are believed to be attributed to lenalidomide administration, please see Table 6 for dose modification guidelines. Doses of any investigational product cannot be re-escalated once reduced. Any investigational product (lenalidomide and/or cetuximab) held > 14 days must be discontinued. If cetuximab is discontinued per protocol and for reasons other than disease progression, lenalidomide may be continued until documented tumor progression, unacceptable toxicity, death, or treatment discontinuation for any other reason.

10.3.1. Lenalidomide Dose Modification or Interruption

Table 6: Dose Modification Guidelines for Lenalidomide

Toxicity	Grade	Dose Modification
Rash	3	Hold lenalidomide until resolution to ≤ Grade 2; at first occurrence resume at same dose of lenalidomide; at second occurrence resume lenalidomide at 5 mg reduction in dose. Further dose reductions are permitted to a minimum of 5 mg.<br>If rash is desquamating, discontinue lenalidomide.
	4	Discontinue lenalidomide.
Sinus bradycardia, atrial fibrillation or other cardiac arrhythmias	2	Hold lenalidomide until resolution to ≤ Grade 1, resume lenalidomide at 5 mg reduction in dose. Additional dose reductions are permitted to a minimum of 5 mg.
	3 / 4	Discontinue lenalidomide.
Neutropenia	3 / 4	Hold lenalidomide, CBC weekly until resolution to ≤ Grade 2, resume lenalidomide at 5 mg reduction in dose. Additional dose reductions are permitted to a minimum of 5 mg.
Thrombocytopenia	3 / 4	Hold lenalidomide, CBC weekly until resolution to ≤ Grade 2, resume lenalidomide at 5 mg reduction in dose. Additional dose reductions are permitted to a minimum of 5 mg.
Venous thrombosis or embolism	3	Continue lenalidomide and anticoagulate.
	4	Discontinue lenalidomide and anticoagulate.
Allergic reaction or hypersensitivity	2 / 3	Hold lenalidomide until resolution to ≤ Grade 1; resume lenalidomide at 5 mg reduction in dose. Additional dose reductions are permitted to a minimum of 5 mg.
	4	Discontinue lenalidomide.
Other non-hematological toxicity assessed as lenalidomide-related	3 / 4	Hold until resolution to ≤ Grade 2; resume lenalidomide at 5 mg reduction in dose. Additional dose reductions are permitted to a minimum of 5 mg.

10.3.2. Cetuximab Dose Modification or Interruption

10.3.2.1. Allergic/hypersensitivity Reactions

The investigator must treat all cases of allergic/hypersensitivity reaction with the best available medical measures. Based on previous experience with cetuximab allergic/hypersensitivity reactions, the treatment guidelines given in Table 7.

Table 7: Treatment Adjustment for Cetuximab-Related Allergic/Hypersensitivity Reactions

CTC Grade Allergic/hypersensitivity reaction	Treatment
Grade 1<br>Transient rash, drug fever <38°C	Decrease the cetuximab infusion rate by 50% and monitor closely for any worsening<br>The total infusion time for cetuximab at the weekly dose should not exceed 240 minutes
Grade 2<br>Urticaria, drug fever ≥38°C and/or asymptomatic bronchospasm	Stop cetuximab infusion<br>Administer bronchodilators, oxygen, etc. as medically indicated<br>Resume infusion at 50% of previous rate once allergic/hypersensitivity reaction has resolved or decreased to grade 1 in severity, and monitor closely for any worsening
Grade 3 or Grade 4<br>Grade 3: Symptomatic bronchospasm, requiring parenteral medication, with or without urticaria; hypersensitivity-related edema, angioedema<br><br>Grade 4: Anaphylaxis	Stop cetuximab infusion immediately and disconnect infusion tubing from the subject<br>Administer epinephrine, bronchodilators, antihistamines, glucocorticoids, intravenous fluids, vasopressor agents, oxygen, etc., as medically indicated<br><br>Subjects have to be withdrawn immediately from treatment and must not receive any further cetuximab treatment

Resumption of treatment following allergic/hypersensitivity reactions:

Once the cetuximab infusion rate has been decreased due to an allergic/hypersensitivity reaction, it must remain decreased for all subsequent infusions. If the subject has a second allergic/hypersensitivity reaction on the slower infusion rate, the infusion should be stopped and the subject should not receive any further cetuximab treatment. If a subject experiences a Grade 3 or 4 allergic/hypersensitivity reaction at any time, cetuximab must be discontinued.

10.3.2.2. Skin Toxicity

If a subject experiences Grade 3 skin toxicity, cetuximab therapy may be delayed for up to 14 days without changing the dose level (see Figure 3). The investigator should also consider concomitant treatment with topical and oral antibiotics; topical corticosteroids are not

recommended. If the toxicity resolves to Grade 2 or less by the following treatment period, treatment may be resumed. If Grade 3 skin toxicity occurs for a second and third time, cetuximab therapy may again be delayed for up to 14 days with concomitant dose reductions to 200 mg/m² and then 150 mg/m². Cetuximab dose reductions are permanent. Subjects must discontinue cetuximab if more than 2 consecutive infusions are withheld or Grade 3 skin toxicity occurs for a fourth time despite appropriate dose reduction.

Figure 3: Treatment Adjustments for Cetuximab-Related Skin Toxicity

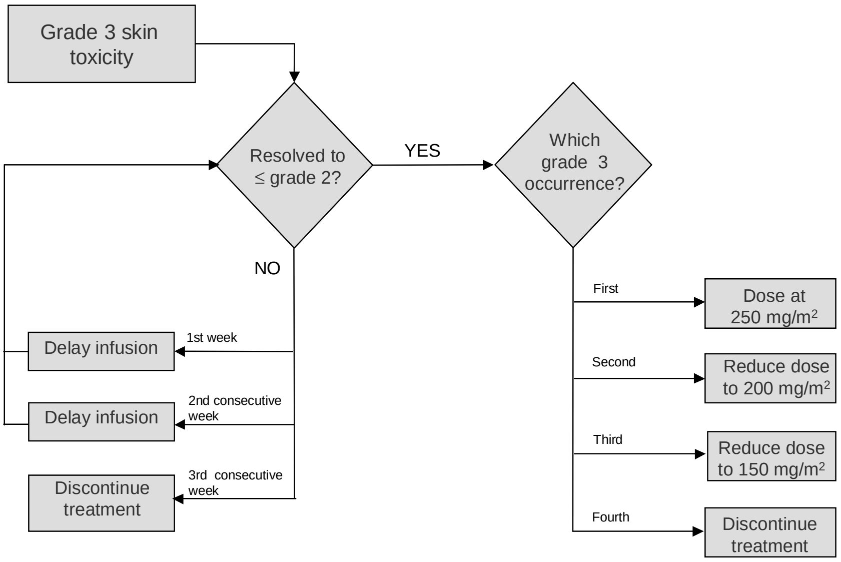

10.3.2.3. Additional Cetuximab Considerations

In addition to omissions and discontinuation for the above reasons, cetuximab may have to be delayed or discontinued if a subject develops an intercurrent illness (e.g. an infection). If in the opinion of the investigator, this warrants interruption of cetuximab therapy, the intercurrent illness must resolve within a time frame such that no more than 2 consecutive infusions are withheld. After the interruption of treatment, the patient will continue with cetuximab at the assigned dose level. The higher initial dose is not repeated.

If therapy must be withheld for > 14 days, the study treatment will be discontinued.

10.4. Prior/Concomitant Medications

All medications (prescription and non-prescription), treatments and therapies taken at the time of study start through 28 days after the last dose of investigational product(s), must be recorded on the appropriate page of the CRF.

10.4.1. Permitted Concomitant Therapy

- Pre-medication for investigational products.

- Full supportive care is permitted when appropriate, including transfusions of blood and blood products, antibiotics, or antiemetics.
- The use of growth factors is permitted according to ASCO guidelines.
- Histamine-receptor antagonists or proton-pump inhibitor therapy is permitted.
- Anticoagulants are permitted.
- Narcotic and non-narcotic analgesics are permitted.

10.4.2. Prohibited Concomitant Therapy

- Treatment with anti-cancer agents is not permitted (except investigational products as per protocol).
- Radiation therapy is not permitted.
- Use of any other experimental drug or therapy is not permitted.

10.5. Treatment Compliance

Study personnel will be instructed to complete pill counts of unused medication at Day 1 of each cycle and at study discontinuation (see appropriate handling instructions in the Pharmacy Manual). Compliance is defined as taking ≥ 70% of the assigned study medication dose during any cycle for reasons other than toxicity or treatment hold in accordance with the protocol. Lack of compliance may lead to discontinuation from the study at the discretion of the Investigator and/or sponsor. Any investigational product (lenalidomide and/or cetuximab) held > 14 days must be discontinued.

10.6. Discontinuation from Treatment

The following events are considered sufficient reasons for discontinuing a subject from investigational product(s):

- Adverse event(s) (AEs) that, in the judgment of the Investigator, may cause severe or permanent harm or which rule out continuation of investigational product(s).
- Disease progression
- Subject withdraws consent
- Subject lost to follow-up
- Death
- Protocol violation

The reason for discontinuation should be recorded in the CRF and in the subject's medical records. An IVRS call is required when discontinuing any investigational product.

11. INVESTIGATIONAL PRODUCT MATERIALS AND MANAGEMENT

11.1. Supplier(s)

Celgene Corporation will supply lenalidomide and commercial cetuximab labeled as IMP.

11.2. Dosage Form

Celgene Corporation will supply lenalidomide as 5, 10, 15, 20, and 25 mg capsules for oral administration.

Celgene Corporation will supply commercial cetuximab labeled as IMP for IV infusion.

11.3. Dosage Regimen

Lenalidomide will be administered PO once daily on Days 1-28 of each 28-day treatment cycle. Lenalidomide should be taken at approximately the same time each day.

Cetuximab will be administered via IV infusion on Days 1, 8, 15 and 22 of each 28-day cycle. The initial dose of cetuximab will be administered at 400 mg/m^2, then 250 mg/m^2 weekly thereafter.

11.4. Investigational Product Packaging and Labeling

Celgene Corporation will supply lenalidomide investigational product packaged in open-label bottles containing enough supply for a 28-day cycle. Each subject will take one capsule per day orally.

Celgene Corporation will supply commercial cetuximab labeled as IMP.

The investigational product(s) label will bear Celgene's name, address, and telephone number, the protocol number, EudraCT number (where required), product name, dosage form and strength, quantity of investigational product per container, lot number, expiration date, medication identification/kit number, dosing instructions, storage conditions, and required caution statements and/or regulatory statements as applicable. Additional information may be included on the label as applicable per local regulations.

Subjects requiring dose reduction within a treatment cycle must return to the study site and return the empty bottle or any unused product and a new bottle will be dispensed.

11.5. Investigational Product Receipt and Storage

The Investigator(s) is responsible for taking an inventory of each shipment of investigational product(s) received, and comparing it with the accompanying investigational product(s) shipping order form. The Investigator(s) will verify the accuracy of the information on the form and call the IVRS to register the study medication received at the site.

At the study site, all investigational product(s) will be stored in a locked, safe area to prevent unauthorized access.

The investigational product should be stored as directed on the product label.

11.6. Record of Administration

Accurate recording of all investigational product(s) administration (including dispensing and dosing) will be kept.

11.7. Investigational Product Accountability

The investigator(s) or designee(s) is responsible for accounting for all investigational product(s) issued to, and returned by, the subject during the course of the study.

11.8. Investigational Product Handling and Disposal

Celgene will instruct the Investigator(s) on drug handling and the return or destruction of unused investigational product(s) per local standard practice. If any investigational product(s) is lost or damaged, its disposition should be documented. Celgene Corporation will provide instructions for the return of investigational product at the end of the study.

12. ASSESSMENT OF EFFICACY

12.1. Assessments

- Tumor assessment.
- Response assessment (RECIST 1.1).
- ECOG performance status.
- Overall survival.

12.2. Methods and Timing of Efficacy Assessments

The efficacy assessments will be conducted at timed intervals during the study as specified in Table 2.

12.2.1. Tumor Assessment

Tumor assessment conventional (or spiral) CT, or MRI will be performed at screening, every 2 cycles beginning with Cycle 3 Day 1, and at treatment discontinuation. Subjects who have received screening scans within 28 days of Cycle 1 Day 1 do not need to have an additional scan done. Discontinuation from study scans do not need to be done if last scan was performed within the prior 28 days. During the study, scans must be done up to 7 days prior to Day 1 of the cycle. To ensure comparability, baseline methods and on-study methods for tumor assessment must be performed using identical techniques. X-rays will be performed only when clinically indicated.

12.2.2. Response Assessment

Tumor response will be evaluated every 2 cycles beginning with Cycle 3 Day 1 and at treatment discontinuation. Response and progression will be evaluated using the RECIST 1.1 criteria (Eisenhauer, 2009). All subjects with evidence of objective tumor response (CR and PR) should have the response confirmed with repeat assessments performed at the next scheduled scan (every 2 cycles following Day 1 Cycle 1).

12.2.3. ECOG Performance Status

ECOG Performance Status will be evaluated during screening, every cycle Day 1 (pre-dose), and at treatment discontinuation.

12.2.4. Survival

All subjects will be followed for survival every 90 days after discontinuation via phone contact until death or 5 years post discontinuation.

13. ASSESSMENT OF SAFETY

13.1. Assessments

- Adverse event reporting.
- Concomitant medications.
- Physical Examinations.
- Vital Signs including height and weight.
- Hematology laboratory assessments.
- Chemistry laboratory assessments.
- Pregnancy testing.
- Thyroid function test.

13.2. Methods and Timing of Safety Assessments

13.2.1. Adverse Event Reporting

All subjects will have AE assessment performed during all visits once the ICF is signed until 28 days after last dose. CTCAE Version 4.0 will be used for grading (http://ctep.cancer.gov/protocolDevelopment/electronic_applications/docs/ctcaev4.pdf).

13.2.2. Concomitant Medications

All subjects will have concomitant medication and procedure assessment performed at all visits once ICF is signed until 28 days after last dose.

13.2.3. Physical Examinations

All subjects will have a physical examination at screening, every cycle Day 1 (pre-dose), at treatment discontinuation, and 28 days after last dose. During Cycle 1, physical examination assessments will occur every 7 days.

13.2.4. Vital Signs

Vital signs (including pulse, blood pressure, and temperature) and weight will be measured at screening, every cycle Day 1 (pre-dose), at treatment discontinuation, and 28 days after last dose. Height will be measured during screening only. During Cycle 1, vital signs will be captured every 7 days.

13.2.5. Hematology Laboratory Assessments

Hematology laboratory including RBC count, hemoglobin, hematocrit, WBC count and differential (including ANC) and platelet count will be collected at screening, every cycle Day 1 (pre-dose), treatment discontinuation, and 28 days after last dose. During Cycle 1, hematology

laboratory parameters will be collected every 7 days. Hematology assessments may be repeated more frequently if clinically indicated.

13.2.6. Chemistry Laboratory Assessments

Serum chemistries including sodium, potassium, chloride, CO_2, calcium, magnesium, phosphorus, BUN, creatinine, glucose, albumin, total protein, alkaline phosphatase, bilirubin, AST/SGOT, ALT/SGPT, LDH and uric acid will be collected at screening, every cycle Day 1 (pre-dose), treatment discontinuation, and 28 days after last dose. During Cycle 1, chemistry laboratory parameters will be collected every 7 days. Chemistry assessments may be repeated more frequently if clinically indicated.

13.2.7. Pregnancy Testing

All WCBP who participate in the study will have urine β-HCG levels assessed as a means of pregnancy testing. The subject may not receive investigational product(s) until the investigator has verified that the results of these pregnancy tests are negative (see specifics in Section 21.3 or 21.4).

13.2.8. Thyroid Function Test

Subjects will have free T3, free T4 and total TSH levels analyzed at screening and at treatment discontinuation. Further analysis of free T3, free T4, and total TSH levels will be performed as clinically indicated.

14. OTHER ASSESSMENTS

14.1. Biomarker Assessment

In addition to testing the safety and effectiveness of the investigational product(s), lenalidomide and cetuximab in this study, an exploratory analysis of biomarkers is planned. Celgene Corporation may attempt to utilize existing and/or develop new biomarker assays designed to identify biological and molecular markers which correlate with evidence of the clinical efficacy and/or safety profile of lenalidomide and cetuximab. These analyses may help identify subjects most likely to benefit from lenalidomide and cetuximab.

Biomarker assessment includes, but is not limited to, immuno flow cytometry panel for T cells and NK cells; assessment of cytokines/immune markers (including, but not limited to, IL-6, TGF, IL-8, IL-10, IL-12, IFN-γ, and GM-CSF); assessment of growth factors (including, but not limit to, vascular endothelial growth factor [VEGF], soluble VEGF-receptor 2 [sVEGFR-2], phosphorylated VEGFR-2, activated platelet-derived growth factor receptor [PDGFR] and placental growth factor [PGF]); and assessment of circulating tumor cells. Additionally, FCγR, KRAS, and EGFR testing will also be conducted using paraffin tumor.

Biomarkers are objectively measured and evaluated indicators of normal biological processes, pathogenic processes, or pharmacologic responses to a therapeutic intervention. In oncology, there is particular interest in the molecular changes underlying the oncogenic processes that may identify cancer subtypes, stage disease, assess the amount of tumor growth or predict disease progression, metastasis and responses to lenalidomide and cetuximab. These investigations may be useful in developing markers to identify disease subtypes, guide therapy and/or predict disease progression.

14.1.1. Preparation/Handling/Shipping of Tumor and Blood Samples for Biomarker Assessment

Please refer to the central laboratory manual for detailed instructions on sample handling, labeling and shipment.

14.1.1.1. Plasma

1. A blood sample will be drawn at Cycle 1 Day 1 (pre-dose), Cycle 2 Day 1, at each tumor assessment, and at treatment discontinuation.
2. The blood will be centrifuged to separate plasma. As much of the plasma as possible is removed with a pipette without disturbing the blood cells and transferred into three vials. Immediately place all 3 tubes vials in a –70°C freezer.
3. The plasma should be frozen within 60 minutes of collection from the subject. The samples should be shipped on dry ice to central laboratory.

14.1.1.2. Blood for Circulating Tumor Cells

1. A blood sample will be drawn at Cycle 1 Day 1 (pre-dose), Cycle 2 Day 1, at each tumor assessment, and at treatment discontinuation.
2. Sample is to be maintained at room temperature and mailed overnight to Celgene Corporation for processing.

14.1.1.3. Blood for Immuno Flow Cytometry

1. A blood sample will be drawn at Cycle 1 Day 1 (pre-dose), Cycle 2 Day 1, at each tumor assessment, and at treatment discontinuation.
2. Sample is to be maintained at room temperature and mailed overnight to central laboratory for processing.

14.1.1.4. Paraffin Blocks or Slides

Paraffin tumor blocks or approximately 25 unstained slides must be sent for KRAS mutation testing and results reported by central pathology laboratory prior to study enrollment. All paraffin tumor blocks will be shipped back to the clinical site once the slides have been cut.

14.1.2. Sample Storage and Destruction

These blood and tumor samples and any other components from the cells may be stored for up to 5 years after the end of the study to research scientific questions related to cancer and/or lenalidomide and cetuximab. Celgene Corporation will be the exclusive owner of any data, discoveries or derivative materials from the sample. If a commercial product is developed based on this research project, Celgene Corporation will own the commercial product and the subject will have no commercial rights to such a product and no rights to the data, information, discoveries, or derivative materials gained or produced from the sample.

14.1.3. Confidentiality

To maximize confidentiality, all biomarker samples and the information associated with the samples will be coded to prevent the exposure of subject’s information and identity. Since these evaluations are neither expected to benefit the subject directly nor to alter their treatment course, the results will not be placed in their medical record and will not be made available to members of their family, their personal physician, or other third parties except as specified in the informed consent.

15. STATISTICAL CONSIDERATIONS

This Phase 2 study in pretreated KRAS mutant subjects with metastatic colorectal cancer consists of three parts. Phase 2a – Safety Lead-In part is to explore the dosing of lenalidomide in combination with cetuximab. Phase 2b – POC part is to explore tumor response rate of lenalidomide in combination with cetuximab and lenalidomide alone. Phase 2b – Expansion part will enroll approximately 120 subjects into the recommended regimen from the POC study and further evaluate the response rate of a lenalidomide containing regimen in pretreated KRAS mutant subjects with metastatic colorectal cancer.

15.1. Study Population Definitions

The population for the study CC-5013-COLO-001 will consist of subjects with histologically confirmed KRAS mutant metastatic CRC measurable by RECIST 1.1 who have failed both oxaliplatin and irinotecan-containing regimens, with at least one of the regimens containing bevacizumab. Study population definitions are as follows:

- Intent-to-Treat (ITT) Population – All subjects who take at least one dose of study medication.
- Safety Population – All subjects who take at least one dose of study medication, which is the same as ITT population for this study.
- Efficacy Evaluable (EE) Population – All ITT subjects who meet eligibility criteria, complete at least one treatment cycle of investigational product(s), and have baseline and at least one post-baseline efficacy assessment.

15.2. Efficacy Evaluation

All treated subjects will be included for efficacy analysis. Efficacy will be analyzed once all subjects have withdrawn from the study or completed at lease 6 cycles. The hypothesis test for the objective confirmed response rate will be based on the exact binomial distribution Phase 2b – Expansion part. Ninety-five percent confidence intervals will be provided. A descriptive analysis of other evidence of anti-tumor activity will be provided based on clinical, radiographic, and biologic assessments of efficacy. If there are sufficient data, Kaplan-Meier estimates will be provided for duration of response, PFS, DCR, and OS.

Data listings will be provided for all relevant data collected during the studies.

15.3. Background and Demographic Characteristics

All analyses will be performed by study Phase and treatment group. The baseline characteristics of treated subjects will be summarized. An accounting will be made of all subjects who received investigational product(s) and, in particular, the number of subjects who died or withdrew during treatment will be specified together with the reasons for withdrawal.

15.4. Investigational product(s)

Descriptive summaries of investigational product(s) exposure will be provided by study Phase and treatment group.

15.5. Safety Evaluation

Data from all subjects who receive one or more doses of drug will be incorporated into the safety analyses. Investigational product(s) exposure will be summarized. Adverse events, vital sign measurements, clinical laboratory information, and concomitant medications will be tabulated and summarized by group and regimen. All toxicities will be summarized by relative and absolute frequency, severity grade based on the CTCAE version 4.0 and relationship to treatment. Serious adverse events (SAE) will be listed separately. Safety information obtained during the Follow-up period during each segment will be incorporated into these analyses. Graphical displays will be provided where useful in the interpretation of results.

15.6. Interim Analyses

There is no plan for interim analysis. However, Phase 2b – POC data will be analyzed and used to recommend the Phase 2b – Expansion part. The primary efficacy analysis will be based on Phase 2b – Expansion phase data alone.

15.7. Sample Size and Power Considerations

During Phase 2a – Safety Lead-in part, up to approximately 18 subjects will be enrolled.

In the Phase 2b – POC part, up to approximately 82 subjects will be randomized in a 1:1 ratio between lenalidomide in combination with cetuximab and lenalidomide alone. A Simon two stage minimax design will be used to monitor subject enrollment for each randomization arm separately. In the first stage, 23 subjects will be enrolled. If ≤ 2 of the 23 subjects (< 10%) have a response in either arm, the enrollment for that arm will be stopped. If > 2 of the 23 subjects have a response in either arm, the enrollment in that arm will continue until 41 subjects are enrolled. If one arm is stopped, all new subjects will be enrolled in the remaining arm. At the final analysis, the regimen will be concluded with more than 10% true response rate if ≥ 9 of 41 subjects (>21%) have a response. This design will have 90% power to conclude the true response rate is higher than 10% at one-sided 2.5% level when the true response rate is 30%.

When any arm from the Phase 2b – POC is considered positive, the study may proceed with that regimen to the Phase 2b –Expansion phase. In the Expansion phase, approximately 120 subjects will be treated with the regimen. This sample size will allow for a two sided 95% confidence interval of (22%, 39%) when 30% response rate is observed.

16. QUALITY CONTROL AND QUALITY ASSURANCE

16.1. Monitoring

Celgene Corporation ensures that appropriate monitoring procedures are performed before, during and after the study. Before the study is initiated at a site visit or at an investigator meeting, all aspects of the study are reviewed with the investigator(s) and the staff. Prior to enrolling subjects into the study, a Celgene Corporation representative will review the protocol, CRFs, procedures for obtaining informed consent, record keeping, and reporting of AEs with the Investigator(s). Monitoring will include on-site visits with the Investigator(s) and his/her staff as well as any appropriate communications by mail, fax, or telephone. At each monitoring visit, the facilities, investigational product(s) storage area, CRFs, subject's source documents, and all other study documentation will be inspected/reviewed by the Celgene Corporation representative for adherence to the protocol and good clinical practice.

At each site visit, the monitor will review CRFs for completion and accuracy. Accuracy will be checked by performing source data verification that is a direct comparison of the entries made onto the CRF against the appropriate source documentation. Any resulting discrepancies will be reviewed with the Investigator(s) and/or his/her staff. Any necessary corrections will be made directly to the CRFs or via source data clarification forms by the Investigator(s) and/or his/her staff. Monitoring procedures require that informed consents, adherence to inclusion/exclusion criteria and documentation of SAEs and the proper recording be verified. Additional monitoring activities may be outlined in a study-specific monitoring plan.

16.2. Audits and Inspections

In addition to the routine monitoring procedures, a Good Clinical Practice Quality Assurance unit exists within Celgene Corporation. From time to time, representatives of this unit will conduct audits of clinical research activities in accordance with Celgene Corporation SOPs to evaluate compliance with Good Clinical Practice guidelines and regulations.

The Investigator(s) is required to permit direct access to the facilities where the study took place, source documents, CRFs and applicable supporting records of subject participation for audits and inspections by IRB/IECs, regulatory authorities (e.g. FDA, EMEA, TPP) and company authorized representatives. The Investigator(s) should make every effort to be available for the audits and/or inspections. If the Investigator(s) is contacted by any regulatory authority regarding an inspection, he/she should contact Celgene Corporation immediately.

16.3. Investigator(s) Responsibilities

Investigator responsibilities are set out in the ICH guideline for Good Clinical Practice and in the US Code of Federal Regulations. Celgene Corporation or a representative will contact and select all principal investigators or co-investigators who in turn will select their staff. The investigator must give the monitor access to relevant records to confirm the above.

The Investigator(s) is responsible for keeping a record of all subjects who sign an Informed Consent Form and are screened for entry into the study. For those subjects who fail screening,

the reason(s) for exclusion must be recorded in the subject's source documents and on the Screening Log provided by Celgene Corporation.

No procedure/assessment/measurement/test other than those outlined here, or in the schedule of study assessments, is to be performed without the prior written approval of Celgene Corporation, or unless deemed by the investigator(s) as necessary for the subject's medical care. Investigator(s) and/or authorized designee(s) must enter study data onto CRFs supplied by Celgene Corporation. The data on the CRF will be recorded in an anonymous manner to protect the subject's identity by using a unique identifier that will prevent personal identifiable information.

The Investigator(s), or a designated member of the Investigators' staff, must be available at some time during monitoring visits to review data and resolve any queries and to allow direct access to the subject's records (e.g., medical records, office charts, hospital charts, and study related charts) for source data verification. The CRFs must be completed as soon as possible after the subject's visit but no later than prior to each monitoring visit and be made available to the Celgene Corporation representative(s) so that the accuracy and completeness may be checked.

17. REGULATORY CONSIDERATIONS

17.1. Institutional Review Board/Independent Ethics Committee Review and Approval

The protocol for this study has been designed in accordance with the general ethical principles outlined in the Declaration of Helsinki (see Section 21.5). The review of this protocol by the IRB/IEC and the performance of all aspects of the study, including the methods used for obtaining informed consent, must also be in accordance with principles enunciated in the declaration, as well as ICH Guidelines, Title 21 of the Code of Federal Regulations (CFR), Part 50 Protection of Human Subjects and Part 56 Institutional Review Boards. Before implementing this study, the protocol, the proposed informed consent form and other information to subjects, must be reviewed by a properly constituted Institutional Review Board/Independent Ethics Committee (IRB/IEC). A signed and dated statement that the protocol and informed consent have been approved by the IRB/IEC must be given to Celgene Corporation before the study initiation. The names and occupations of the chairman and the members of the IRB/IEC must be supplied to Celgene Corporation.

The Investigator(s) will be responsible for preparing documents for submission to the relevant IRB/IEC and obtaining written approval for this study. The approval will be obtained prior to the initiation of the study.

A copy of the IRB/IEC approval for the protocol and the Informed Consent is to be provided to Celgene Corporation. The approval for both the protocol and informed consent must specify the date of approval, protocol number and version, or amendment number.

The Investigator(s) is responsible for notifying the IRB/IEC of any serious deviations from the protocol, or anything else that may involve added risk to subjects.

Any advertisements used to recruit subjects for the study must be reviewed and approved by Celgene Corporation and the IRB/IEC prior to use.

17.2. Protocol Amendments

Any amendment to this protocol that seems appropriate, as the study progresses (e.g. affects safety or efficacy) will be agreed upon between the coordinating and/or principal investigator(s) and the Celgene Corporation study physician. Amendments will be submitted to the IRB/IEC for written approval before the implementation of the amended version. The written signed approval from the IRB/IEC should refer specifically to the investigator(s) and to the protocol number and title and mention any amendment numbers that are applicable. Amendments that are administrative in nature do not require IRB/IEC approval but will be submitted to the IRB/IEC for information purposes.

17.3. Informed Consent

The Investigator(s) must obtain informed consent of a subject or his/her designee prior to any study related procedures as per Good Clinical Practices (GCP) as set forth in 21 CFR Parts 50 and 56 and ICH guidelines.

Documentation that informed consent occurred prior to the subject's entry into the study and of the informed consent process should be recorded in the subject's source documents. The original consent form, signed and dated by the subject and by the person consenting the subject prior to the subject's entry into the study, must be maintained in the Investigator's study files and a copy given to the subject. In addition, if a protocol is amended and it impacts on the content of the informed consent, the informed consent must be revised. Subjects participating in the study when the amended protocol is implemented must be re-consented with the revised version of the informed consent. The revised consent form, signed and dated by the subject and by the person consenting the subject, must be maintained in the Investigator's study files and a copy given to the subject.

17.4. Subject Confidentiality

Celgene Corporation affirms the subject's right to protection against invasion of privacy. In compliance with United States federal regulations, Celgene Corporation requires the Investigator(s) to permit Celgene Corporation's representatives and, when necessary, representatives of the FDA or other regulatory authorities to review and/or copy any medical records relevant to the study in accordance with local laws.

Should direct access to medical records require a waiver or authorization separate from the subject's statement of informed consent, it is the responsibility of the Investigator(s) to obtain such permission in writing from the appropriate individual.

18. DATA HANDLING AND RECORDKEEPING

18.1. Data/Documents

The investigator(s) must ensure that the records and documents pertaining to the conduct of the study and the distribution of the investigational product(s), that is, copies of CRFs and source documents, original documents, data, and records (e.g., hospital records; clinical and office charts; laboratory notes; memoranda; subject's diaries or evaluation checklists; pharmacy dispensing records; recorded data from automated instruments; copies or transcriptions certified after verification as being accurate copies; microfiches; photographic negatives, microfilm, or magnetic media; x-rays; subject files) and records kept at the pharmacy, at the laboratories, and at medico-technical departments involved in the clinical study are complete, accurate, and filed and retained.

18.2. Data Management

Data will be entered into the clinical database. These data will be electronically verified through use of on-line checks during data entry, and through programmed edit checks specified by the clinical team. Discrepancies in the data will be brought to the attention of the clinical team, and investigational site personnel, if necessary, in the form of a Data Clarification Form (DCF). Resolutions to these issues will be reflected in the database. An audit trail within the system will track all changes made to the data. A quality control audit will be performed.

18.3. Retention of Records

The investigator(s) must maintain records of all study documents and supporting information relating to the conduct of the study. This documentation includes, but is not limited to, protocols, case report forms, advertising for subject participation, adverse event reports, subject source data, correspondence with health authorities and IRBs/IECs, informed consent forms, investigator(s) curricula vitae, monitor visit logs, laboratory reference ranges, laboratory certification or quality control procedures and laboratory director curriculum vitae. Subject files and other source data must be kept for the maximum period of time permitted by the hospital, institution or private practice specified below. The study monitor must be consulted if the investigator(s) wishes to assign the study files to someone else, remove them to another location or is unable to retain them for a specified period.

For studies conducted in the United States under a US IND, the investigator(s) must retain the study records for a minimum of 2 years after a marketing application for the indication is approved, or for 2 years after the IND is withdrawn. If no application is filed, or if the application is not approved for the indication, the records are to be retained for two years after the investigation (i.e., the IND) is discontinued, and FDA is notified of that fact. For IND studies conducted outside the US, the investigator(s), must retain study records for the time period described above or according to local laws or requirements, whichever is longer. The monitor will inform the investigator(s) of the dates for retention. All study documents should be made available if required by relevant health authorities. For studies not conducted under the US IND, the investigator(s) records must be retained until at least 2 years after the last approval of a

marketing application in an ICH region and until there are no pending or contemplated marketing applications in an ICH region or at least 2 years have elapsed since the formal discontinuation of clinical development of the investigational product. These documents should be retained for a longer period if required by other applicable regulatory requirements.

19. PREMATURE DISCONTINUATION OF THE STUDY

19.1. Single Site

The responsible clinical Investigator as well as Celgene Corporation have the right to discontinue a single site at any time during the study for reasonable medical or administrative reasons. Possible reasons for termination of the study could be, but are not limited to:

- Unsatisfactory enrollment with respect to quantity or quality
- Inaccurate or incomplete data collection
- Falsification of records
- Failure to adhere to the study protocol

19.2. Study as a Whole

Celgene Corporation reserves the right to terminate this clinical study at any time for reasonable medical or administrative reasons.

Any possible premature discontinuation would have to be documented adequately with reasons being stated, and information would be issued according to local requirements (e.g., IRB/EC, regulatory authorities, etc.).

20. REFERENCES

Amado RG, et al. Wild-type KRAS is required for panitumumab efficacy in patients with metastatic colorectal cancer. J Clin Oncol. 2008;26(10):1626-34.

Bartlett JB, et al. Phase I study to determine the safety, tolerability and immunostimulatory activity of thalidomide analogue CC-5013 in patients with metastatic melanoma and other advanced cancers. British J Cancer 2004;90:944-961.

Bartlett J, et al. The evoloution of thalidomide and its IMiD derivatives as anticancer agents. Nature Reviews Cancer 2004 Apr; 4:314-322.

Carethers JM. Systemic treatment of advanced colorectal cancer: Tailoring therapy to the tumor. Therapeutic Advances in Gastroenterology. 2008; 1(1):33-42.

De Roock W, et al. KRAS wind-type state predicts survival and is associated to early radiological response in metastatic colorectal cancer treated with cetuximab. Annals of Oncology 2008 Nov 12;19:508-515.

Dredge K, et al. Orally administered lenalidomide (cc-5013) is anit-angiogenic in vivo and inhibits endothelial cell migration and Akt phosphorylation in vitro. Microvasc Res 2005 Jan 2; 69(1-2):56-63.

Dredge K, et al. Novel thalidomide analogues display anti-angiogenic activity independently and immunomodulatory effects. Br J Cancer 2002; 87:1166-1172.

Eisenhauer EA, et al. New response evaluation criteria in solid tumours: revised RECIST guideline (version 1.1). Eur J Cancer. 2009 Jan;45(2):228-47.

Finocchiaro G, et al. EGFR, HER2 and Kras as predictive factors for cetuximab sensitivity in colorectal cancer. J Clin Oncol. 2007;25(18S): Abstract 4021.

Freeman DJ, et al. Association of K-ras mutational status and clinical outcomes in patients with metastatic colorectal cancer receiving panitumumab alone. Clin Colorectal Cancer. 2008;7(3):184-90.

Galustian C, et al. The anti-cancer agents lenalidomide and CC-4047 inhibit the proliferation and function of T regulatory cells. Cancer Immunol Immunother 2008 (in press).

Global Cancer Facts and Figures, 2007. Retrieved from: http://www.cancer.org/downloads/STT/Global_Cancer_Facts_and_Figures_2007_rev.pdf.

Haslett P, et al. Thalidomide and a thalidomide analogue drug costimulate virus-specific CD8+ T cells in vitro. The Journal of Infectious Diseases 2003;187:946-55.

Hecht JR, et al. Panitumumab efficacy in patients with Metastatic colorectal cancer with low or undetectable level of EGFR: Final efficacy and KRAS analysis. Presented at: the ASCO: 2008 Gastrointestinal symposium, January 25-27 2008; Orlando, FL.

Hernandez-Ilizaliturri F, et al. Immunomodulatory drug CC-5013 or CC-4047 and rituximab enhance antitumor activity in a severe combined immunodeficient mouse lymphoma model. Clin Cancer Res 2005 Aug 15; 11(6): 5984-5992.

Karapetis CS, et al. K-ras mutations and benefit from cetuximab in advanced colorectal cancer. N Engl J Med. 2008;359(17):1757-65.

Khambata-Ford S, et al. Expression of epiregulin and amphiregulin and K-ras mutation status predict disease control in metastatic colorectal cancer patients treated with cetuximab. J Clin Oncol 2007;25:3230-7.

Lievre A, et al. KRAS mutations as an independent prognostic factor in patients with advanced colorectal cancer treated with cetuximab. J Clin Oncol 2008;26:374-79.

Lu L, et al. The anti-cancer drug lenalidomide inhibits angiogenesis and metastasis via multiple inhibitory effects on endothelial cell function in normoxic and hypoxic conditions. Microvasc Res 2008 Sep 4. [Epub ahead of print].

Mayer A, et al. The prognostic significance of proliferating cell nuclear antigen, epidermal growth factor receptor, and mdr gene expression in colorectal cancer. Cancer 1993;71(8): 2454–2460.

Messa C, et al. EGF, TGF-alpha, and EGF-R in human colorectal adenocarcinoma. Acta Oncol 1998;37(3): 285–289.

Mitsiades S, Mitsiades N. CC-5013 Celgene. Current Opinion in Investigational Drugs 2004;5(6):635-647.

National Comprehensive Cancer Network (NCCN) Guidelines, 2008. Retrieved from: URL:http://www.nccn.org/professionals/physician_gls/PDF/colon.pdf.

Noonan K, et al. The immunomodulatory role of lenalidomide on Prevnar responses in patients with relapsed multiple myeloma: a comprehensive analysis of the immune response. American Society of Hematology 2008 Dec 6-9; Abstract #2772.

Oken, MM, et al. Toxicity And Response Criteria Of The Eastern Cooperative Oncology Group. Am J Clin Oncol. 1982;5:649-655.

Peeters M, et al. Anti-epidermal growth factor receptor monotherapy in the treatment of metastatic colorectal cancer: where are we today? Oncologist. 2009;14(1):29-39

Petrylak DP, et al. A phase 1 open-label study using lanalidomide and docetaxel in castration-resistant prostate cancer. J Clin Oncol. 2009;27(15S): Abstract 5156.

Porebska I, et al. Expression of the tyrosine kinase activity growth factor receptors (EGFR, ERB B2, ERB B3) in colorectal adenocarcinomas and adenomas. Tumour Biol 2000; 21(2):105–115.

Ramsay A, et al. Chronic lymphocytic leukemia T cells show impaired immunological synapse formation that can be reversed with an immunomodulating drug. J Clin Invest 2008; 118:2427-2437.

Reddy N, et al. Immunomodulatory drugs stimulate natural killer-cell function, alter cytokine production by dendritic cells, and inhibit angiogenesis enhancing the anti-tumour activity of rituximab in vivo. Br J Haematol 2007; 140:36-45.

Sanborn SL, et al. Phase I trial of docetaxel given every 3 weeks and daily lenalidomide in patients with advanced solid tumors. Invest New Drugs. 2008 Nov 15.

Tohnya TM et al. Phase I study of lenalidomide, a novel thalidomide analog, in patients with refractory metastatic cancer. J Clin Oncol 2006; 24:13038.

Wai M, et al. Thalidomide, lenalidomide and pomalidomide reduce the metastatic capability of cancer cells in vivo and in vitro. American Association for Cancer Research (AACR) Annual Meeting 2008 Apr 12-16; Abstract 1173.

Wolpin BM, et al. Systemic treatment of colorectal cancer. Gastroenterology. 2008;134(5):1296-310.

Wu L, et al. Lenalidomide enhances natural killer cell and monocyte-mediated antibody-dependent cellular cytotoxicity of rituximab-treated CD20 tumor cells. Clin Cancer Res 2008 Jul 15;14(14): 4650.

Wu L, et al. Effect of lenalidomide and pomalidomide combined with IgG1-isotype antibodies on antibody-dependent cellular cytotoxicity (ADCC) via cytokine signaling and effector cell granzyme B and FasL expression. American Society of Clinical Oncology (ASCO) Annual Meeting 2008 May30-Jun 3; Abstract #3058.

Zhu, et al. Immunomodulatory drugs Revlimid((R)) (lenalidomide) and CC-4047 induce apoptosis of both hematological and solid tumor cells through NK cell activation. Cancer Immunol Immunother 2008:57:1849-59.

21. APPENDICES

21.1. ECOG Performance Status Scale (Oken, 1982)

Score	Description
0	Fully active, able to carry on all pre-disease performance without restriction.
1	Restricted in physically strenuous activity but ambulatory and able to carry out work of a light or sedentary nature, e.g., light housework, office work.
2	Ambulatory and capable of all self-care but unable to carry out any work activities. Up and about more than 50% of waking hours.
3	Capable of only limited self-care, confined to bed or chair more than 50% of waking hours.
4	Completely disabled. Cannot carry on any self-care. Totally confined to bed or chair.
5	Dead.

21.2. Adverse Event

An adverse event (AE) is any noxious, unintended, or untoward medical occurrence occurring at any dose that may appear or worsen in a subject during the course of a study. It may be a new intercurrent illness, a worsening concomitant illness, an injury, or any concomitant impairment of the subject's health, including laboratory test values (as specified by the criteria below), regardless of etiology. Any medical condition that was present prior to study treatment and that remains unchanged or improved should not be recorded as an AE. If there is a worsening of that medical condition, this should be considered an AE. A diagnosis or syndrome should be recorded on the AE page of the Case Report Form rather than the individual signs or symptoms of the diagnosis or syndrome.

All AEs will be recorded by the Investigator(s) from the time of signing ICF to 28 days after the last dose. AEs will be recorded on the AE page of the CRF and in the subject's source documents.

Abnormal laboratory values defined as adverse events

An abnormal laboratory value is considered to be an AE if the laboratory abnormality is characterized by any of the following:

- Results in discontinuation from the study.
- Requires treatment, modification/interruption of investigational product(s) dose, or any other therapeutic intervention.
- Is judged by the Investigator(s) to be of significant clinical importance.

If a laboratory abnormality is one component of a diagnosis or syndrome, then only the diagnosis or syndrome should be recorded on the AE page of the CRF. If the abnormality was not a part of a diagnosis or syndrome, then the laboratory abnormality should be recorded as the AE.

Serious adverse event

A serious adverse event (SAE) is any AE which:

- Results in death
- Is life-threatening (i.e., in the opinion of the Investigator(s) the subject is at immediate risk of death from the AE)
- Requires inpatient hospitalization or prolongation of existing hospitalization
- Results in persistent or significant disability/incapacity (a substantial disruption of the subject's ability to conduct normal life functions)
- Is a congenital anomaly/birth defect
- Constitutes an important medical event

Important medical events are defined as those occurrences that may not be immediately life threatening or result in death, hospitalization, or disability, but may jeopardize the subject or require medical or surgical intervention to prevent one of the other outcomes listed above.

Medical and scientific judgment should be exercised in deciding whether such an AE should be considered serious.

Events not considered to be SAEs are hospitalizations which: were planned before entry into the clinical study; are for elective treatment of a condition unrelated to the studied indication or its treatment; occur on an emergency outpatient basis and do not result in admission (unless fulfilling other criteria above); are part of the normal treatment or monitoring of the studied indication and are not associated with any deterioration in condition.

If an AE is considered serious, both the AE pages of the CRF and the SAE Report Form must be completed.

For each SAE, the Investigator(s) will provide information on severity, start and stop dates, relationship to investigational product(s), action taken regarding investigational product(s), and outcome.

Classification of severity

For both AEs and SAEs, the investigator(s) must assess the severity of the event. The severity of AEs will be graded based upon the subject’s symptoms according to CTCAE, Version 4.0 (http://ctep.cancer.gov/protocolDevelopment/electronic_applications/docs/ctcaev4.pdf). AEs that are not defined in the NCI CTCAE should be evaluated for severity according to the following scale:

Grade 1 = Mild

Grade 2 = Moderate

Grade 3 = Severe

Grade 4 = Life threatening

Grade 5 = Death

Classification of Relationship/Causality of adverse events (SAE/AE) to investigational product(s)

The Investigator(s) must determine the relationship between the administration of investigational product(s) and the occurrence of an AE/SAE as Not Suspected or Suspected as defined below:

Not suspected: The temporal relationship of the adverse event to investigational product(s) administration makes **a causal relationship unlikely or remote**, or other medications, therapeutic interventions, or underlying conditions provide a sufficient explanation for the observed event

Suspected: The temporal relationship of the adverse event to investigational product(s) administration makes **a causal relationship possible,** and other medications, therapeutic interventions, or underlying conditions do not provide a sufficient explanation for the observed event.

Monitoring and reporting of adverse events

All subjects will be monitored for AEs during the study. Assessments may include monitoring of any or all of the following parameters: the subject's clinical symptoms; laboratory, pathological, radiological, or surgical findings; physical examination findings; or other appropriate tests and procedures.

Immediate reporting of serious adverse events

Any AE that meets the criterion for an SAE requires the completion of an SAE Report Form in addition to being recorded on the AE pages of the CRF. The Investigator(s) is required to ensure that the data on these forms is accurate and consistent. This applies to all SAEs, regardless of relationship to investigational product(s), that occur during the study, those made known to the Investigator(s) within 28 days after a subject's last dose of investigational product(s), and those made known to the investigator(s) at anytime that are suspected of being related to investigational product(s).

The SAE must be reported immediately (i.e., within 24 hours of the Investigators' knowledge of the event) to the Celgene Corporation Drug Safety by facsimile. An initial written report (prepared by the Investigator(s) using the SAE Report Form provided by Celgene Corporation) is to be faxed to Celgene Corporation Drug Safety (see below for contact information).

The SAE report should provide a detailed description of the SAE and include copies of hospital records and other relevant documents. If a subject has died and an autopsy has been performed, copies of the autopsy report and death certificate are to be sent to Celgene Corporation as soon as these become available. Any follow-up data will be detailed in a subsequent SAE Report Form, and sent to Celgene Corporation.

The Investigator(s) is responsible for informing the Institutional Review Board/Ethics Committee (IRB/IEC) of the SAE and providing them with all relevant initial and follow-up information about the event. The Investigator(s) must keep copies of all SAE information, including correspondence with Celgene Corporation and the IRB/IEC on file. All SAEs that have not resolved upon discontinuation of the subject's participation in the study must be followed until either the event resolves completely, stabilizes/resolves with sequelae, or returns to baseline (if a baseline value is available).

Pregnancies

Pregnancies and suspected pregnancies (including a positive pregnancy test regardless of age or disease state) of a female subject or the female partner of a male subject occurring while the subject is on investigational product(s), or within 28 days of the subject's last dose of investigational product(s), are considered immediately reportable events. Investigational product(s) is to be discontinued immediately and the subject instructed to return any unused portion of the investigational product(s) to the investigator(s). The pregnancy, suspected pregnancy, or positive pregnancy test must be reported to Celgene Corporation Drug Safety by facsimile using the SAE Report Form.

The female should be referred to an obstetrician-gynecologist, preferably one experienced in reproductive toxicity, for further evaluation and counseling.

The Investigator(s) will follow the female subject until completion of the pregnancy, and must notify Celgene Corporation Drug Safety of the outcome of the pregnancy as a follow-up to the initial SAE report.

If the outcome of the pregnancy meets the criteria for immediate classification as an SAE (i.e., spontaneous or therapeutic abortion [any congenital anomaly detected in an aborted fetus is to be documented], stillbirth, neonatal death, or congenital anomaly [including that in an aborted fetus]), the Investigator(s) should follow the procedures for reporting SAEs (i.e., report the event to Celgene Corporation Drug Safety by facsimile within 24 hours of the Investigator's knowledge of the event).

In the case of a live "normal" birth, Celgene Corporation Drug Safety should be notified by facsimile within 24 hours of the Investigator's knowledge of the event.

All neonatal deaths that occur within 28 days of birth should be reported, without regard to causality, as SAEs. In addition, any infant death after 28 days that the Investigator(s) suspects is related to the in utero exposure to the investigational product(s) should also be reported to Celgene Corporation Drug Safety by facsimile within 24 hours of the Investigators' knowledge of the event.

If the female is found not to be pregnant, any determination regarding the subject's continued participation in the study will be determined by the Investigator(s) and the Celgene Corporation Medical Monitor.

Expedited Reporting of Adverse Events

For the purpose of regulatory reporting, Celgene Corporation Drug Safety will determine the expectedness of events suspected of being related to lenalidomide based on the Investigator Brochure.

For countries within the European Economic Area (EEA), Celgene Corporation will report in an expedited manner to Regulatory Authorities and Ethics Committees concerned, SUSARs in accordance with Directive 2001/20/EC and the Detailed Guidance on collection, verification and presentation of adverse reaction reports arising from clinical trials on medicinal products for human use (ENTR/CT3) and also in accordance with country-specific requirements.

For the purpose of regulatory reporting in the EEA, Celgene Corporation Drug Safety will determine the expectedness of events suspected of being related to the other investigational medicinal product (IMP), cetuximab, based on the SmPC for this product.

Celgene Corporation shall notify the Investigator of the following information:

Any AE associated with the use of investigational product(s) in this study or in other studies that is both serious and unexpected, i.e., suspected unexpected serious adverse reaction (SUSAR)

Any finding from tests in laboratory animals that suggests a significant risk for human subjects including reports of mutagenicity, teratogenicity, or carcinogenicity.

Where required by local legislation, the Investigator shall notify his/her IRB/IEC promptly of these new serious and unexpected AE(s) or significant risks to subjects.

The Investigator must keep copies of all pertinent safety information, including correspondence with Celgene Corporation and the IRB/IEC, on file (see Section 18.3 for records retention information).

Celgene Corporation Drug Safety Contact Information:

For Local Drug Safety Affiliate Office contact information, please refer to the Serious Adverse Event Report Form Completion Guidelines.

21.3. Lenalidomide Pregnancy Risk Minimisation Plan for Celgene Clinical Trials (Ex-EU territories)

Section 21.3 applies to all patients receiving lenalidomide therapy. The following Pregnancy Risk Minimisation Plan documents are included in this section:

1. Lenalidomide Risks of Fetal Exposure, Pregnancy Testing Guidelines and Acceptable Birth Control Methods (Section 21.3.1);
2. Lenalidomide Education and Counselling Guidance Document (Section 21.3.2);
3. Lenalidomide Patient Card (Section 21.3.3) and,
4. Lenalidomide Information Sheet (Section 21.3.4).

1. The Lenalidomide Risks of Fetal Exposure, Pregnancy Testing Guidelines and Acceptable Birth Control Methods document (Section 21.3.1) provides the following information:
   - Risks to the foetus associated with lenalidomide exposure
   - Definition of Woman of Childbearing Potential
   - Pregnancy testing requirements for patients receiving Lenalidomide who are women of childbearing potential
   - Acceptable birth control methods for both woman of childbearing potential and male patients receiving Lenalidomide in the study
   - Requirements for counselling of all study patients receiving Lenalidomide about pregnancy precautions and the potential risks of foetal exposure to lenalidomide
2. The Lenalidomide Education and Counselling Guidance Document (Section 21.3.2) must be completed and signed by either a trained counsellor or the Investigator at the participating clinical centre prior to each dispensing of lenalidomide study treatment. A copy of this document must be maintained in the patient records.
3. The Lenalidomide Patient Card (Section 21.3.3) will be completed by the Investigator for each patient receiving lenalidomide study therapy prior to the start of lenalidomide study treatment. For women of childbearing potential, all study-related pregnancy tests will be recorded on this patient card.

The Lenalidomide Information Sheet (Section 21.3.4) will be given to each patient receiving lenalidomide study therapy. The patient must read this document prior to starting lenalidomide study treatment.

21.3.1. Lenalidomide Risks of Fetal Exposure, Pregnancy Testing Guidelines and Acceptable Birth Control Methods

Risks Associated with Pregnancy

Lenalidomide is structurally related to thalidomide. Thalidomide is a known human teratogenic active substance that causes severe life-threatening birth defects. An embryofoetal development study in animals indicates that lenalidomide produced malformations in the offspring of female monkeys who received the drug during pregnancy. The teratogenic effect of lenalidomide in humans cannot be ruled out. Therefore, a risk minimization plan to prevent pregnancy must be observed.

Criteria for woman of childbearing potential (WCBP)

This protocol defines a woman of childbearing potential as a sexually mature woman who: 1) has not undergone a hysterectomy or bilateral oophorectomy or 2) has not been naturally postmenopausal (amenorrhea following cancer therapy does not rule out childbearing potential) for at least 24 consecutive months (i.e., has had menses at any time in the preceding 24 consecutive months).

Counselling

For a woman of childbearing potential, lenalidomide is contraindicated unless all of the following are met (i.e., all women of childbearing potential must be counselled concerning the following risks and requirements prior to the start of lenalidomide study therapy):

- She understands the potential teratogenic risk to the unborn child
- She understands the need for effective contraception, without interruption, 4 weeks before starting study treatment, throughout the entire duration of study treatment, dose interruption and 28 days after the end of study treatment
- She should be capable of complying with effective contraceptive measures
- She is informed and understands the potential consequences of pregnancy and the need to notify her study doctor immediately if there is a risk of pregnancy
- She understands the need to commence the study treatment as soon as lenalidomide is dispensed following a negative pregnancy test
- She understands the need and accepts to undergo pregnancy testing based on the frequency outlined in this protocol (Table 2).
- She acknowledges that she understands the hazards and necessary precautions associated with the use of lenalidomide

The investigator must ensure that for women of childbearing potential:

- Complies with the conditions for pregnancy risk minimization, including confirmation that she has an adequate level of understanding
- Acknowledge the aforementioned requirements

For a woman NOT of childbearing potential, lenalidomide is contraindicated unless all of the following are met (i.e., all women NOT of childbearing potential must be counselled concerning the following risks and requirements prior to the start of lenalidomide study therapy):

- She acknowledges that she understands the hazards and necessary precautions associated with the use of lenalidomide

Traces of lenalidomide have been found in semen. Male patients taking lenalidomide must meet the following conditions (i.e., all males must be counselled concerning the following risks and requirements prior to the start of lenalidomide study therapy):

- Understand the potential teratogenic risk if engaged in sexual activity with a woman of childbearing potential
- Understand the need for the use of a condom even if he has had a vasectomy, if engaged in sexual activity with a woman of childbearing potential.

Contraception

Women of childbearing potential (WCBP) enrolled in this protocol must agree to use two reliable forms of contraception simultaneously or to practice complete abstinence from heterosexual intercourse during the following time periods related to this study: 1) for at least 28 days before starting investigational product; 2) while participating in the study; 3) dose interruptions; and 4) for at least 28 days after study treatment discontinuation.

The two methods of reliable contraception must include one highly effective method and one additional effective (barrier) method. WCBP must be referred to a qualified provider of contraceptive methods if needed. The following are examples of highly effective and additional effective methods of contraception:

- Highly effective methods:
  - Intrauterine device (IUD)
  - Hormonal (birth control pills, injections, implants)
  - Tubal ligation
  - Partner’s vasectomy
- Additional effective methods:
  - Male condom
  - Diaphragm
  - Cervical Cap

Implants and levonorgestrel-releasing intrauterine systems are associated with an increased risk of infection at the time of insertion and irregular vaginal bleeding. Prophylactic antibiotics should be considered particularly in patients with neutropenia.

Pregnancy testing

Medically supervised pregnancy tests with a minimum sensitivity of 25 mIU/mL must be performed for women of childbearing potential, including women of childbearing potential who commit to complete abstinence, as outlined below.

Before starting investigational product

Female Patients:

WCBP must have two negative pregnancy tests (sensitivity of at least 25 mIU/mL) prior to starting lenalidomide. The first pregnancy test must be performed within 10-14 days prior to the start of lenalidomide and the second pregnancy test must be performed within 24 hours prior to the start of lenalidomide. The patient may not receive investigational product until the Investigator has verified that the results of these pregnancy tests are negative.

Male Patients:

Must practice complete abstinence or agree to use a condom during sexual contact with women of childbearing potential while participating in the study, during dose interruptions and for at least 28 days following investigational product discontinuation, even if he has undergone a successful vasectomy.

During study participation and for 28 days following investigational product discontinuation

Female Patients:

- WCBP with regular or no menstrual cycles must agree to have pregnancy tests weekly for the first 28 days of study participation and then every 28 days while on study, at study discontinuation, and at day 28 following investigational product discontinuation. If menstrual cycles are irregular, the pregnancy testing must occur weekly for the first 28 days and then every 14 days while on study, at study discontinuation, and at days 14 and 28 following investigational product discontinuation.
- At each visit, the Investigator must confirm with the WCBP that she is continuing to use two reliable methods of birth control at each visit.
- Counselling about pregnancy precautions and the potential risks of foetal exposure must be conducted at a minimum of every 28 days.
- If pregnancy or a positive pregnancy test does occur in a study patient, lenalidomide must be immediately discontinued.
- Pregnancy testing and counselling must be performed if a patient misses her period or if her pregnancy test or her menstrual bleeding is abnormal. Investigational product treatment must be discontinued during this evaluation.
- Women must agree to abstain from breastfeeding during study participation and for at least 28 days after investigational product discontinuation.

Male Patients:

- Counselling about the requirement for condom use during sexual contact with women of childbearing potential and the potential risks of foetal exposure must be conducted at a minimum of every 28 days.
- If pregnancy or a positive pregnancy test does occur in the partner of a male study patient during study participation, the investigator must be notified immediately.

Additional precautions

- Patients should be instructed never to give this medicinal product to another person and to return any unused capsules to the Investigator at the end of treatment.
- Female patients should not donate blood during therapy and for at least 28 days following discontinuation of lenalidomide.
- Male patients should not donate blood, semen or sperm during therapy or for at least 28 days following discontinuation of lenalidomide.

Only enough lenalidomide for one cycle of therapy may be dispensed with each cycle of therapy.

21.3.2. Lenalidomide Education and Counselling Guidance Document

Protocol Number: ____

Patient Name (Print): __________________ DOB: _____/_____/_____ (mm/dd/yyyy)

Female:

If female, check one:

- ❑ WCBP (Woman of childbearing potential): sexually mature female who: 1) has not undergone a hysterectomy (the surgical removal of the uterus) or bilateral oophorectomy (the surgical removal of both ovaries) or 2) has not been naturally postmenopausal (amenorrhea following cancer therapy does not rule out childbearing potential) for at least 24 consecutive months (i.e., has had menses at any time during the preceding 24 consecutive months)
- ❑ NOT WCBP

Male:

To be completed prior to each dispensing of lenalidomide.

<u>Do Not Dispense lenalidomide if:</u>

- The patient is pregnant.
- No pregnancy tests were conducted for a WCBP.
- The patient states she/he did not use TWO reliable methods of birth control (unless practicing complete abstinence of heterosexual intercourse) [at least 28 days prior, during, dose interruption and 28 days after discontinuation of lenalidomide].

<u>WCBP:</u>

1. I verified that the required pregnancy tests performed are negative.
2. I counselled WCBP regarding the following:
   - Potential foetal harm: If lenalidomide is taken during pregnancy, it may cause birth defects or death to any unborn baby. Females are advised to avoid pregnancy while taking lenalidomide. The teratogenic potential of lenalidomide in humans cannot be ruled out. WCBP must agree not to become pregnant while taking lenalidomide.
   - Using TWO reliable methods of birth control at the same time or complete abstinence from heterosexual intercourse [at least 28 days prior, during, dose interruption and 28 days after discontinuation of lenalidomide].
   - Continuation of TWO reliable methods of birth control or complete abstinence if therapy is interrupted.
   - That even if she has amenorrhea she must comply with advice on contraception

- Use of one highly effective method and one additional method of birth control AT THE SAME TIME. The following are examples of highly effective and additional effective methods of contraception:
  - Highly effective methods:
    - Intrauterine device (IUD)
    - Hormonal (birth control pills, injections, implants)
    - Tubal ligation
    - Partner's vasectomy
  - Additional effective methods:
    - Male condom
    - Diaphragm
    - Cervical Cap
- Pregnancy tests before and during treatment, even if the patient agrees not to have reproductive heterosexual intercourse. Two pregnancy tests will be performed prior to receiving investigational product, one within 10-14 days and the second within 24 hours of the start of lenalidomide.
- Frequency of pregnancy tests to be done:
  - Every week during the first 28 days of this study and a pregnancy test every 28 days during the patient's participation in this study if menstrual cycles are regular or every 14 days if cycles are irregular.
  - If the patient missed a period or has unusual menstrual bleeding.
  - When the patient is discontinued from the study and at day 28 after investigational product discontinuation if menstrual cycles are regular. If menstrual cycles are irregular, pregnancy tests will be done at discontinuation from the study and at days 14 and 28 after investigational product discontinuation.
- Stop taking lenalidomide immediately in the event of becoming pregnant and to call their study doctor as soon as possible.
- NEVER share lenalidomide with anyone else.
- Do not donate blood while taking lenalidomide and for 28 days after stopping lenalidomide.
- Do not breastfeed a baby while participating in this study and for at least 28 days after investigational product discontinuation.
- Do not break, chew, or open lenalidomide capsules.
- Return unused lenalidomide to the investigator.

3. Provide Lenalidomide Information Sheet to the patient.

WOMEN NOT OF CHILDBEARING POTENTIAL (NATURAL MENOPAUSE FOR AT LEAST 24 CONSECUTIVE MONTHS, A HYSTERECTOMY, OR BILATERAL OOPHORECTOMY):

1. I counselled the woman NOT of child bearing potential regarding the following:
   - Potential foetal harm (Refer to item #2 in WCBP)
   - NEVER share lenalidomide with anyone else.
   - Do not donate blood while taking lenalidomide and for 28 days after stopping lenalidomide.
   - Do not break, chew, or open lenalidomide capsules
   - Return used lenalidomide capsules to the Investigator.
2. Provide Lenalidomide Information Sheet to the patient.

MALE:

1. I counselled the Male patient regarding the following:
   - Potential foetal harm (Refer to item #2 in WCBP).
   - To engage in complete abstinence or use a condom when engaging in sexual intercourse (including those who have had a vasectomy) with a woman of childbearing potential, while taking lenalidomide, during dose interruptions and for 28 days after stopping lenalidomide.
   - Males should notify their study doctor when their female partner becomes pregnant and female partners of males taking lenalidomide should be advised to call their healthcare provider immediately if they get pregnant
   - NEVER share lenalidomide with anyone else.
   - Do not donate blood, semen or sperm while taking lenalidomide and for 28 days after stopping lenalidomide.
   - Do not break, chew, or open lenalidomide capsules.
   - Return unused lenalidomide capsules to the investigator.

2. Provide Lenalidomide Information Sheet to the patient.

Investigator/Counsellor Name (Print): ____________________
(circle applicable)

Investigator/Counsellor Signature: ________________________ Date: _____/_____/_____
(circle applicable)

**Maintain a copy of the Education and Counselling Guidance Document in the patient records. **

21.3.3. Lenalidomide Patient Card

Protocol Number:____

Subject study ID (Print): ___________________ DOB: _____/_____/_____ (mm/dd/yyyy)

Investigator to complete each section.

1. Status of Patient (pick one)

- Woman NOT of childbearing potential* ☐

(*no monitoring of pregnancy tests required. Retain card in records)

- Male ☐

- Woman of child bearing potential ☐

2. Counselling regarding the potential teratogenicity of lenalidomide and the need to avoid pregnancy has been provided before first dose dispensed.

Investigator's Signature

Date

3. For Woman of Childbearing potential

Date of Visit	Date of NEGATIVE pregnancy test	Confirmed no risk of pregnancy	Date lenalidomide prescribed	Investigator's signature

21.3.4. Lenalidomide Information Sheet

FOR PATIENTS ENROLLED IN CLINICAL RESEARCH STUDIES

Please read this Lenalidomide Information Sheet before you start taking lenalidomide and each time you get a new supply, since there may be new information. This Lenalidomide Information Sheet does not take the place of an informed consent to participate in clinical research or talking to your study doctor or healthcare provider about your medical condition or your treatment.

What is the most important information I should know about lenalidomide?

1. **Lenalidomide may cause birth defects (deformed babies) or death of an unborn baby.** Lenalidomide is similar to the medicine thalidomide. It is known that thalidomide causes life-threatening birth defects. Lenalidomide has not been tested in pregnant women but may also cause birth defects. Preliminary findings from a monkey study appear to indicate that lenalidomide caused birth defects in the offspring of female monkeys who received the drug during pregnancy**.**

If you are a woman who is able to become pregnant:

- Do not take lenalidomide if you are pregnant or plan to become pregnant
  - for 28 days before starting lenalidomide
  - while taking lenalidomide
  - during dose interruptions of lenalidomide
  - for 28 days after stopping lenalidomide
- Stop taking lenalidomide if you become pregnant during lenalidomide treatment
- Do not breastfeed while taking lenalidomide
- You must have pregnancy testing done at the following times:
  - within 10 – 14 days and again 24 hours prior to the first dose of lenalidomide
  - weekly for the first 28 days
  - every 28 days after the first month or every 14 days if you have irregular menstrual periods
  - if you miss your period or have unusual menstrual bleeding
  - 28 days after the last dose of lenalidomide (14 and 28 days after the last dose if menstrual periods are irregular)
- You must practice complete abstinence or use two reliable, separate forms of effective birth control at the same time:
  - for 28 days before starting lenalidomide

  - while taking lenalidomide
  - during dose interruptions of lenalidomide
  - and for 28 days after stopping lenalidomide

- The study doctor will be able to advise you where to get additional advice on contraception.
- If you suspect you are pregnant at any time during the study, you must stop lenalidomide immediately and immediately inform your study doctor. The study doctor will report all cases of pregnancy to Celgene Corporation.
- In order to ensure that an unborn baby is not exposed to lenalidomide, your study doctor will complete a Patient Card documenting that you have been informed of the requirement for you NOT to become pregnant during treatment with lenalidomide and for 28 days after stopping treatment.

If you are a woman not of childbearing potential:

In order to ensure that an unborn baby is not exposed to lenalidomide, your study doctor will complete a Patient Card documenting that you are not able to become pregnant.

If you are a male:

Lenalidomide is detected in trace quantities in human semen. The risk to the foetus in women of child bearing potential whose male partner is receiving lenalidomide is unknown at this time.

- Male patients must practice complete abstinence or must use a condom during sexual intercourse with a pregnant woman or a woman who that can become pregnant (including those who have had a vasectomy),:
  - While you are taking lenalidomide
  - During dose interruptions of lenalidomide
  - For 28 days after you stop taking lenalidomide
- **Male patients should not donate sperm or semen** while taking lenalidomide and for 28 days after stopping lenalidomide.
- **If you suspect that your partner is pregnant any time during the study, you must immediately inform your study doctor. The study doctor will report all cases of pregnancy to Celgene Corporation.**

Lenalidomide restrictions in sharing lenalidomide and donating blood:

- Do not share lenalidomide with other people. It must be kept out of the reach of children and should never be given to any other person.
- Do not give blood while you take lenalidomide and for 28 days after stopping lenalidomide.

- Do not break, chew, or open lenalidomide capsules.
- You will get no more than a 28-day supply of lenalidomide at one time.
- Return unused lenalidomide capsules to your study doctor.

Additional information is provided in the informed consent form and you can ask your study doctor for more information.

21.4. Lenalidomide Pregnancy Prevention Risk Management Plan (Europe)

Section 21.4 applies to all patients randomized to receive lenalidomide. The following pregnancy prevention Risk Management Plan documents are included in this section) Lenalidomide Risks of Fetal Exposure, Pregnancy Testing Guidelines and Acceptable Birth Control Methods (Section 21.4.1); 2) Lenalidomide Education and Counseling Guidance Document (Section 21.4.2); 3) Lenalidomide Patient Card (Section 21.4.3) and 4) Lenalidomide Information Sheet (Section 21.4.4).

The Lenalidomide Risks of Fetal Exposure, Pregnancy Testing Guidelines and Acceptable Birth Control Methods document (Section 21.4.1) provides the following information:

- Risks to the fetus associated with lenalidomide exposure
- Definition of Woman of Childbearing Potential
- Pregnancy testing requirements for patients receiving lenalidomide who are women of childbearing potential
- Acceptable birth control methods for both woman of childbearing potential and male patients receiving lenalidomide
- Requirements for counseling of all study patients receiving lenalidomide about pregnancy precautions and the potential risks of fetal exposure to lenalidomide

The Lenalidomide Education and Counseling Guidance Document (Section 21.4.2) must be completed and signed by a trained counselor at the participating clinical center prior to the first dispensing of lenalidomide. A copy of this document must be maintained in the patient records.

The Lenalidomide Patient Card (Section 21.4.3) will be completed by the Investigator for each patient at the clinical site randomized to receive lenalidomide prior to the start of treatment. For women of childbearing potential, all study-related pregnancy tests will be recorded on this patient card.

The Lenalidomide Information Sheet (Section 21.4.4) will be given to each patient randomized to receive lenalidomide. The patient must read this document prior to starting lenalidomide and when the patient receives a new supply of lenalidomide for each new cycle of treatment.

21.4.1. Lenalidomide Risks of Fetal Exposure, Pregnancy Testing Guidelines and Acceptable Birth Control Methods (Europe)

Pregnancy warning

Lenalidomide is related to thalidomide. Thalidomide is known to cause severe life-threatening human birth defects. Preliminary findings from a monkey study appear to indicate that lenalidomide caused birth defects in the offspring of female monkeys who received the drug during pregnancy. If lenalidomide is taken during pregnancy, it may cause birth defects or death to any unborn baby. Women must not become pregnant while taking lenalidomide. If you are female, you agree not to become pregnant while taking lenalidomide.

You must NEVER share lenalidomide with someone else.

The conditions of the Pregnancy Prevention Program must be fulfilled for all patients unless there is reliable evidence that the patient does not have childbearing potential.

Criteria for women of childbearing potential (WCBP)

A woman of childbearing potential is a sexually mature woman who: 1) has not undergone a hysterectomy or bilateral oophorectomy or 2) has not been naturally postmenopausal (amenorrhea following cancer therapy does not rule out childbearing potential) for at least 24 consecutive months (i.e., has had menses at any time in the preceding 24 consecutive months).

Counseling

For a woman of childbearing potential, lenalidomide is contraindicated unless all of the following are met (i.e., all women of childbearing potential must be counseled concerning the following risks and requirements prior to the start of study therapy):

- She understands the potential teratogenic risk to the unborn child
- She understands the need for effective contraception, without interruption, 4 weeks before starting treatment, throughout the entire duration of treatment, and 4 weeks after the end of treatment
- Even if a woman of childbearing potential has amenorrhea she must follow all the advice on effective contraception
- She should be capable of complying with effective contraceptive measures
- She is informed and understands the potential consequences of pregnancy and the need to rapidly consult if there is a risk of pregnancy
- She understands the need to commence the treatment as soon as lenalidomide is dispensed following a negative pregnancy test
- She understands the need and accepts to undergo pregnancy testing every 4 weeks except in case of confirmed tubal sterilisation
- She acknowledges that she understands the hazards and necessary precautions associated with the use of lenalidomide

For a woman NOT of childbearing potential, lenalidomide is contraindicated unless all of the following are met (i.e., all women NOT of childbearing potential must be counseled concerning the following risks and requirements prior to the start of study therapy):

- She understands the potential teratogenic risk to the unborn child
- She acknowledges that she understands the hazards and necessary precautions associated with the use of lenalidomide

For male patients taking lenalidomide, clinical data is available demontrating the presence of lenalidomide in human semen. Male patients taking lenalidomide must meet the following conditions (i.e., all males must be counseled concerning the following risks and requirements prior to the start of study therapy):

- Understand the potential teratogenic risk if engaged in sexual activity with a woman of childbearing potential
- Understand the need for the use of a condom if engaged in sexual activity with a woman of childbearing potential

The investigator must ensure that for women of childbearing potential:

- The patient complies with the conditions for Pregnancy Prevention Programme, including confirmation that she has an adequate level of understanding
- The patient has acknowledged the aforementioned conditions

Contraception

Women of childbearing potential must use one effective method of contraception for 4 weeks before starting lenalidomide, while taking lenalidomide, and until 4 weeks after discontinuation of lenalidomide and even in case of dose interruption unless the patient commits to absolute and continuous abstinence confirmed on a monthly basis. If not established on effective contraception, the patient must be referred to an appropriately trained health care professional for contraceptive advice in order that contraception can be initiated.

The following can be considered to be examples of suitable methods of contraception:

- Implant
- Levonorgestrel-releasing intrauterine system (IUS)
- Medroxyprogesterone acetate depot
- Tubal sterilisation
- Sexual intercourse with a vasectomised male partner only; vasectomy must be confirmed by two negative semen analyses
- Ovulation inhibitory progesterone-only pills (i.e., desogestrel)

Implants and levonorgestrel-releasing intrauterine systems are associated with an increased risk of infection at the time of insertion and irregular vaginal bleeding. Prophylactic antibiotics should be considered particularly in patients with neutropenia.

Copper-releasing intrauterine devices are generally not recommended due to the potential risks of infection at the time of insertion and menstrual blood loss which may compromise patients with neutropenia or thrombocytopenia.

Pregnancy testing

According to local practice, medically supervised pregnancy tests with a minimum sensitivity of 25 mIU/mL must be performed for women of childbearing potential, including women of childbearing potential who commit to abstinence, as outlined below.

Prior to starting treatment

Two medically supervised pregnancy tests should be performed during the protocol screening period. The first pregnancy test must be performed within 10-14 days prior to the start of lenalidomide and the second pregnancy test must be performed within 24 hours prior to the start of lenalidomide.

Follow-up and end of treatment

A medically supervised pregnancy test should be repeated every 4 weeks, including 4 weeks after the end of treatment, except in the case of confirmed tubal sterilisation. These pregnancy tests should be performed on the day of the study visit or in the 3 days prior to the visit to the investigator.

Men

It is known that lenalidomide is present in semen. Therefore all male patients should use condoms throughout treatment duration, during dose interruption and for 1 week after cessation of treatment if their partner is of childbearing potential.

Additional precautions

Ideally, pregnancy testing, issuing and dispensing lenalidomide should occur on the same day. Dispensing of lenalidomide should occur at the day of the study visit.

Patients should be instructed never to give this medicinal product to another person and to return any unused capsules to their pharmacist at the end of treatment.

Patients should not donate blood or semen during therapy or for 1 week following discontinuation of lenalidomide.

21.4.2. Lenalidomide Education and Counseling Guidance Document (Europe)

Protocol Number:____

Patient Name (Print): __________________ DOB: _____/_____/_____ (mm/dd/yyyy)

To be completed at start of therapy:

Counseling for Women of Childbearing Potential

Prior to the first dispense of lenalidomide, I have counseled the patient on the following:

- The potential teratogenic risk to the unborn child
- The need for effective contraception (implant, levonorgestrel-releasing intrauterine system (IUS), medroxyprogesterone acetate depot, tubal sterilisation, sexual intercourse with a vasectomised male only: vasectomy must be confirmed by two negative semen analyses, ovulatory inhibitory progesterone only pill- desogestrel) 4 weeks before starting treatment, during treatment interruption, throughout the entire duration of treatment and for 4 weeks after the end of treatment or absolute and continued abstinence
- The need to undergo pregnancy testing at 4 weekly intervals unless confirmed tubal sterilisation
- That even if she has amenorrhoea she must comply with advice on contraception
- The potential consequences of pregnancy and the need to stop treatment and consult rapidly if there is a risk of pregnancy
- The hazards and necessary precautions associated with use of lenalidomide
- Not to share medication
- To return unused capsules to Investigator/study staff
- Not to donate blood whilst taking lenalidomide or for one week after stopping

Counseling for Women NOT of Child Bearing Potential

Prior to the first dispense of lenalidomide, I have counseled the patient on the following:

- not to share medication
- to return unused capsules to Investigator/study staff
- not to donate blood whilst taking lenalidomide or for one week after stopping

Counseling for men

Prior to the first dispense of lenalidomide, I have counseled the patient on the following;

- The need to use condoms throughout treatment duration, during dose interruption, and for one week after cessation of treatment if partner is of childbearing potential.
- Not to share medication
- To return unused capsules to Investigator/study staff
- Not to donate blood or semen whilst taking lenalidomide or for one week after stopping

Investigator's Name (Print): ____________________

Investigator's Signature: ________________________ Date: _____/_____/_____

**Maintain a copy of the Education and Counseling Guidance Document in the patient records.

21.4.3. Lenalidomide Patient Card (Europe)

Protocol Number:____

Patient Name (Print): ___________________ DOB: _____/_____/_____ (mm/dd/yyyy)

Investigator to complete each section.

1. Status of Patient (tick one)

- Woman NOT of childbearing potential* ☐

(*no monitoring of pregnancy tests required. Retain card in records)

- Male ☐
- Woman of child bearing potential ☐

2. Counseling regarding the potential teratogenicity of lenalidomide and the need to avoid pregnancy has been provided before first dose dispensed.

Investigator's Signature

Date

3. For Woman of Childbearing potential

Date of Visit	Date of NEGATIVE pregnancy test	Confirmed no risk of pregnancy	Date lenalidomide prescribed	Investigator's signature

21.4.4. Lenalidomide Information Sheet (Europe)

FOR PATIENTS ENROLLED IN CLINICAL RESEARCH STUDIES

Please read this Lenalidomide Information Sheet before you start taking lenalidomide and each time you get a new supply, since there may be new information. This Lenalidomide Information Sheet does not take the place of an informed consent to participate in clinical research or talking to your study doctor or healthcare provider about your medical condition or your treatment.

Information for Women Patients of Child Bearing Potential

- Lenalidomide may be harmful to the unborn child.
- In order to ensure that an unborn baby is not exposed to lenalidomide, you doctor will complete a Patient Card documenting that you have been informed of the requirement for you (or your partner) NOT to become pregnant during treatment with lenalidomide and for one month after finishing lenalidomide.
- You should never share lenalidomide with anyone else
- You should always return any unused capsules to the Investigator/study staff
- You should not donate blood during treatment or for one week after treatment finishes
- The most common, serious side effects of lenalidomide are a reduction in the number of blood cells that fight infection and also the blood cells that help the blood to clot. For this reason your doctor will arrange for you to have blood tests weekly for at least the first 8 weeks of treatment. Lenalidomide may also cause thrombosis (blood clots in the veins). Therefore you must tell your doctor immediately if you experience:
  - any fever, chills, sore throat, cough, mouth ulcers or any other symptoms of infection
  - any bleeding or bruising in the absence of injury
  - any chest or leg pain
  - any shortness of breath.
- If you experience any side effects whilst taking lenalidomide you should tell your doctor

Pregnancy Prevention Program

- Do not take lenalidomide if you are pregnant or think you may be pregnant or are planning to become pregnant, as **lenalidomide may be harmful to an unborn child**
- If you are able to become pregnant, you must follow all the necessary measures to prevent you becoming pregnant and ensuring you are not pregnant during treatment. Before starting the treatment, you should ask your doctor if you are able to become pregnant, even if you think this is unlikely.
- If you are able to become pregnant, you will have pregnancy tests under the supervision of your doctor before treatment. These will be repeated every 4 weeks during treatment and 4 weeks after the treatment has finished unless it is confirmed that you have had a tubal sterilisation)
- If you are able to become pregnant you must use effective methods of contraception for 4 weeks before starting treatment, during treatment, and until 4 weeks after stopping treatment. Your doctor will advise you on appropriate methods of contraception, as some types of contraception are not recommended with lenalidomide. It is essential therefore that you discuss this with your doctor
- Your doctor will be able to advise you where to get advice on contraception
- If you suspect you are pregnant at any time whilst taking lenalidomide or in the 4 weeks after stopping, you must stop lenalidomide immediately and immediately inform your doctor.

Information for Women Patients Not of Child Bearing Potential

- **Lenalidomide may be harmful to the unborn child.**
- In order to ensure that an unborn baby is not exposed to lenalidomide, you doctor will complete a Patient Card documenting that you are not able to become pregnant
- You should never share lenalidomide with anyone else
- You should always return any unused capsules to the Investigator/study staff
- You should not donate blood during treatment or for one week after treatment finishes
- The most common, serious side effects of lenalidomide are a reduction in the number of blood cells that fight infection and also the blood cells that help the blood to clot. For this reason your doctor will arrange for you to have blood tests weekly for at least the first 8 weeks of treatment. Lenalidomide may also cause thrombosis (blood clots in the veins). Therefore you must tell your doctor immediately if you experience:
  - any fever, chills, sore throat, cough, mouth ulcers or any other symptoms of infection
  - any bleeding or bruising in the absence of injury
  - any chest or leg pain
  - any shortness of breath.
- If you experience any side effects whilst taking lenalidomide you should tell your doctor

Information for male patients

- **Lenalidomide may be harmful to the unborn child.**
- In order to ensure that an unborn baby is not exposed to lenalidomide, you doctor will complete a Patient Card documenting that you have been informed of the requirement for your partner NOT to become pregnant during treatment with lenalidomide and for one month after you finish lenalidomide.
- You should never share lenalidomide with anyone else
- You should always return any unused capsules to the Investigator/study staff
- You should not donate blood or semen during treatment or for one week after treatment finishes
- The most common, serious side effects of lenalidomide are a reduction in the number of blood cells that fight infection and also the blood cells that help the blood to clot. For this reason your doctor will arrange for you to have blood tests weekly for at least the first 8 weeks of treatment. Lenalidomide may also cause thrombosis (blood clots in the veins). Therefore you must tell your doctor immediately if you experience:
  - any fever, chills, sore throat, cough, mouth ulcers or any other symptoms of infection
  - any bleeding or bruising in the absence of injury
  - any chest or leg pain
  - any shortness of breath.
- If you experience any side effects whilst taking lenalidomide you should tell your doctor
- It is known that lenalidomide passes into human semen. If your partner is able to become pregnant, and she doesn’t use effective contraception, you must use condoms, during treatment, during dose interruptions and 1 week after the end of treatment even if you have had a vasectomy.
- If your partner does become pregnant whilst you are taking lenalidomide, you should inform your treating doctor immediately.

21.5. Declaration of Helsinki

The Declaration of Helsinki can be found at: http://www.wma.net/e/policy/b3.htm.
